# Targeting human CALR‐mutated MPN progenitors with a neoepitope‐directed monoclonal antibody

**DOI:** 10.15252/embr.202152904

**Published:** 2022-02-14

**Authors:** Denis Tvorogov, Chloe A L Thompson‐Peach, Johannes Foßelteder, Mara Dottore, Frank Stomski, Suraiya A Onnesha, Kelly Lim, Paul A B Moretti, Stuart M Pitson, David M Ross, Andreas Reinisch, Daniel Thomas, Angel F Lopez

**Affiliations:** ^1^ Centre for Cancer Biology SA Pathology and University of South Australia Adelaide SA Australia; ^2^ Cancer Program Precision Medicine Theme South Australian Health and Medical Research Institute (SAHMRI) University of Adelaide Adelaide SA Australia; ^3^ Discipline of Medicine Adelaide Medical School The University of Adelaide Adelaide SA Australia; ^4^ Department of Internal Medicine Division of Haematology Medical University of Graz Graz Austria; ^5^ Department of Haematology Flinders University and Medical Centre Adelaide SA Australia; ^6^ Department of Blood Group Serology and Transfusion Medicine Medical University of Graz Graz Austria

**Keywords:** calreticulin, monoclonal antibody, myelofibrosis, myeloproliferative neoplasm, stem cell progenitor, Cancer, Immunology, Signal Transduction

## Abstract

Calreticulin (CALR) is recurrently mutated in myelofibrosis via a frameshift that removes an endoplasmic reticulum retention signal, creating a neoepitope potentially targetable by immunotherapeutic approaches. We developed a specific rat monoclonal IgG2α antibody, 4D7, directed against the common sequence encoded by both insertion and deletion mutations. 4D7 selectively bound to cells co‐expressing mutant CALR and thrombopoietin receptor (TpoR) and blocked JAK‐STAT signalling, TPO‐independent proliferation and megakaryocyte differentiation of mutant CALR myelofibrosis progenitors by disrupting the binding of CALR dimers to TpoR. Importantly, 4D7 inhibited proliferation of patient samples with both insertion and deletion CALR mutations but not JAK2 V617F and prolonged survival in xenografted bone marrow models of mutant CALR‐dependent myeloproliferation. Together, our data demonstrate a novel therapeutic approach to target a problematic disease driven by a recurrent somatic mutation that would normally be considered undruggable.

## Graphical abstract

CALR is recurrently mutated in myelofibrosis, creating a surface‐exposed neoepitope that activates the thrombopoietin receptor. A monoclonal antibody (4D7) with mutation‐specific efficacy in CALR mutant progenitors disrupts the receptor‐CALR complex and inhibits proliferation.

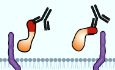

## Introduction

Myeloproliferative neoplasms (MPNs) are haematological stem‐cell‐derived proliferation disorders, commonly characterized by an expansion of the myeloid lineage of cells and constitutive activation of the signalling pathways involved in haematopoiesis. MPNs can be categorized into three main subtypes: primary myelofibrosis (PMF), polycythaemia vera (PV) and essential thrombocytopaenia (ET), with both ET and PV having the potential to deteriorate to PMF (Tefferi & Pardanani, 2015). Common mutually exclusive driver mutations within Janus Kinase 2 (*JAK2*), Calreticulin (*CALR*) and myeloproliferative leukaemia virus oncogene (*MPL*) are often identified in MPN patients. While *JAK2* mutations are exclusive to PV, the mutational status of these genes is not specifically associated with a particular MPN (Tefferi & Vainchenker, 2011).

Primary myelofibrosis is the most severe Philadelphia‐negative MPN and is characterized by marrow fibrosis and chronic inflammatory symptoms with a 5‐year survival of less than 50% (Baade *et al*, 2019). Although rare, PMF affects both young and older adults and can evolve into acute leukaemia in > 15% of cases (Passamonti *et al*, 2012). Mutations within *CALR*, the second most common genetic aberration associated with PMF, are observed in 70% of non‐JAK2^V617F^ and non‐MPL cases (Klampfl *et al*, 2013; Nangalia *et al*, 2013) and are found in 20–30% of ET (Tefferi *et al*, 2014). Importantly, patients with *CALR* mutations do not effectively respond to JAK inhibitor therapy and no *CALR*‐specific therapy has been developed (Ross *et al*, 2021).

Virtually, all *CALR* mutations identified in PMF are small insertions or deletions clustered within exon 9. The two most common mutations identified include a 52 bp deletion (type 1) or a 5 bp insertion (type 2) (Klampfl *et al*, 2013; Nangalia *et al*, 2013). These frameshift mutations lead to a neoepitope peptide sequence which is thought to directly or indirectly activate the thrombopoietin receptor (TpoR) by a poorly defined mechanism that is dependent on glycan‐binding sites, N‐terminal chaperone domain and the novel C‐terminal tail of the mutant protein (Araki *et al*, 2016; Chachoua *et al*, 2016; Elf *et al*, 2016; Marty *et al*, 2016). All somatic *CALR* mutations observed in MPNs result in a +1 frameshift, leading to a common altered peptide sequence with loss of the negative‐charged C‐terminal calcium‐binding domain, gain of a lysine/arginine‐rich segment followed by a stop codon and loss of the KDEL sequence that constitutes an endoplasmic reticulum retention signal (Klampfl *et al*, 2013; Nangalia *et al*, 2013). Recently, it has been demonstrated that without this localization signal, mutant CALR protein is passively secreted from cells, and is detectable in cultured cell supernatants (Han *et al*, 2016; Liu *et al*, 2020; Masubuchi *et al*, 2020), with evidence that TpoR activation occurs after cell surface exposure, implying it may be accessible to an extracellularly acting therapeutic (How *et al*, 2019). Interestingly, recent data suggest that multimerization of mutant CALR monomers is absolutely required for mutant CALR TPO‐independent proliferation (Araki *et al*, 2019). There are subtle differences in prognosis and biochemistry between type 1 and type 2 *CALR* mutations (How *et al*, 2019), which are classified by the extent of elimination of negatively charged residues in the mutant protein compared to wild type. Ideally, a therapeutic would have activity against both type 1 and type 2 *CALR* mutations with minimal to no effect on normal haematopoiesis.

Here, we demonstrate a novel therapeutic strategy for MPNs by developing a monoclonal antibody with specificity for the mutant CALR peptide that inhibits TpoR activation through a distinct mechanism. Treatment of mutant CALR cells with the 4D7 monoclonal antibody inhibits binding of mutant CALR to the TpoR, blocking TpoR tyrosine phosphorylation and constitutive STAT and ERK phosphorylation. Biologically, 4D7 blocks TPO‐independent megakaryocyte differentiation from patients with both type 1 and type 2 frameshift mutations and prolong survival in cell‐line xenograft models of mutant CALR‐driven proliferation. Together, our data demonstrate a novel approach to target frameshift mutations in cancer that were previously considered undruggable.

## Results and Discussion

**Figure 1:**
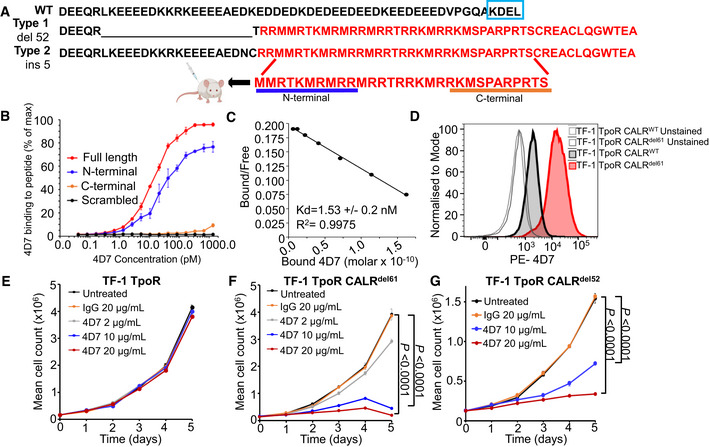
Anti‐mutant calreticulin antibody binds to cell surface and inhibits TPO‐independent proliferation Schematic showing wild‐type C‐terminal calreticulin protein sequence and neoepitope sequences for 52 bp deletion or 5 bp insertion and peptide sequence used for immunization.Diluting concentrations of 4D7 to bound full‐length 30 amino acid peptide compared to scrambled, C‐terminal and N‐terminal 11 amino acid peptides as shown in A (*n* = 3 biological replicates).Scatchard analysis showing dissociation constant of 4D7 using ^125^I‐labelled and unlabelled 4D7 bound to full‐length 30 amino acid peptide (*n* = 3 biological replicates).Histogram showing fluorescence intensity of 4D7 conjugated to phycoerythrin at a 4 µg/ml TF‐1 TpoR CALR^del61^ vs. TF‐1 TpoR CALR^WT^ compared to unstained.TF‐1 TpoR cells with endogenous wild‐type CALR cultured in the presence of TPO and 2, 10 or 20 µg/ml 4D7 anti‐mutant CALR antibody or 20 µg/ml control IgG antibody (*n* = 3 biological replicates).Proliferation curves of factor‐independent TF‐1 TpoR CALR^del61^ cells cultured with 2, 10 or 20 µg/ml 4D7 or 20 µg/ml of control IgG antibody (*n* = 3 biological replicates).Proliferation curves of factor‐independent TF‐1 TpoR CALR^del52^ cells cultured with 10 or 20 µg/ml 4D7 or 20 µg/ml of control IgG antibody (*n* = 3 biological replicates). Data information: For panels (E–G), bars represent standard error of the mean summarizing three biological replicates performed in triplicate, with a one‐way ANOVA with Bonferroni correction used to determine statistical significance.

**Figure EV1:**
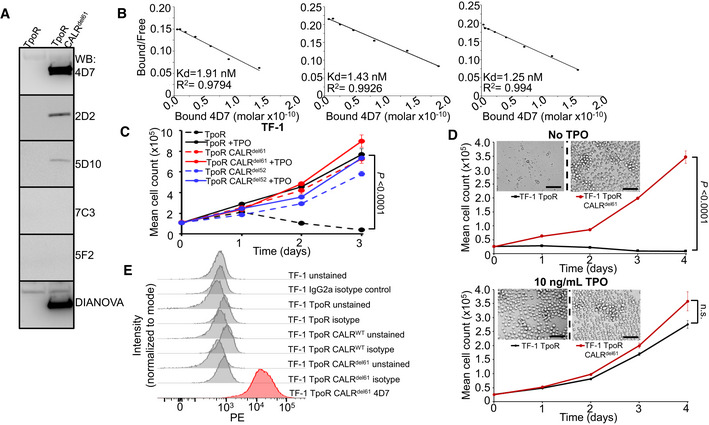
Characterization of 4D7 antibody as a potential therapeutic tool Western blot screening of various mutant CALR antibody clones produced from hybridomas in TF‐1 TpoR and TF‐1 TpoR CALR^del61^ cells. Varying levels of intensity can be observed compared to commercial Dianova monoclonal mutCALR antibody. 4D7 is able to detect mutant CALR protein in CALR^del61^ cells but not in CALR wild‐type TF‐1 cells.Biological replicate Scatchard analyses of 4D7 using ^125^I‐labelled and unlabelled 4D7 bound to full‐length peptide (*n* = 3 technical replicates).TF‐1 cells expressing TpoR and CALR^del61^ or CALR^del52^ demonstrate factor independence in absence of TPO. Cells were cultured in the presence or absence of 10 ng/ml TPO (*n* = 3 biological replicates).Paracrine CALR‐mutant protein is not sufficient to maintain TPO‐sensitive cells in culture. TF‐1 TpoR and TF‐1 TpoR CALR^del61^ cells were seeded at the same density and cultured in the presence or absence of TPO. Cells were separated by semi‐permeable membrane in a horizontal co‐culture system. Cell populations on either side of the membrane were counted every 24 h over 4 days in triplicate and representative images were taken on day 4. Exogenous CALR secreted by TF‐1 TpoR CALR^del61^ was unable to assist growth of factor‐dependent TF‐1 TpoR cells. Scale bar indicates 100 µm (*n* = 3 biological replicates).Histogram overlays showing fluorescence intensity of unstained and PE‐conjugated IgG2a isotype control in TF‐1, TF‐1 TpoR, TF‐1 TpoR CALR^WT^ and TF‐1 TpoR CALR^del61^ compared to 4D7 conjugated to PE. Source data are available online for this figure.

Both type 1 and type 2 *CALR* mutations in myelofibrosis result in a loss of the endoplasmic reticulum retention signal KDEL producing aberrant localization of mutant protein (Klampfl *et al*, 2013; Nangalia *et al*, 2013; How *et al*, 2019) and the acquisition of a 31 amino‐acid‐long sequence towards the C‐terminus (Fig 1A). We reasoned that this neoepitope would be an ideal target for precision medicine approaches and set out to develop a panel of rat monoclonal antibodies to a chemically synthesized peptide corresponding to the C‐terminal mutant CALR neoepitope sequence (Fig 1A). Of three hybridoma screens, several antibodies showed positive binding to the peptide and to the CALR‐mutant protein in immunoblotting with clone 4D7 showing the strongest reactivity (Fig EV1A). By using peptides covering different regions of the immunizing neopeptide, we mapped the binding of 4D7 to the 11 amino acids of the N‐terminus with no binding of 4D7 observed to a scrambled peptide or the C‐terminal portion of the immunogen (Fig 1B). Clone 4D7 mAb showed a dissociation constant (*K*_d_) of 1.53 nM to the full‐length peptide as measured by ^125^I‐Scatchard analysis (Figs 1C and EV1B). To investigate the specific binding of 4D7 on *CALR* mutant‐bearing cells, we generated cytokine‐independent TF‐1 TpoR CALR^del61^ and TF‐1 TpoR CALR^del52^ (Fig EV1C). Using these cells in a horizontal co‐culturing system, we found no evidence of a paracrine effect by the mutant CALR protein (Fig EV1D). Specific binding of phycoerythrin (PE)‐conjugated 4D7 to the cell surface of mutant CALR‐expressing cells, but not TF‐1 TpoR CALR^WT^‐expressing cells, was observed in comparison to unstained or isotype‐PE control (Figs 1D and EV1E).

### Monoclonal antibody 4D7 blocks TPO‐independent signalling

**Figure EV2:**
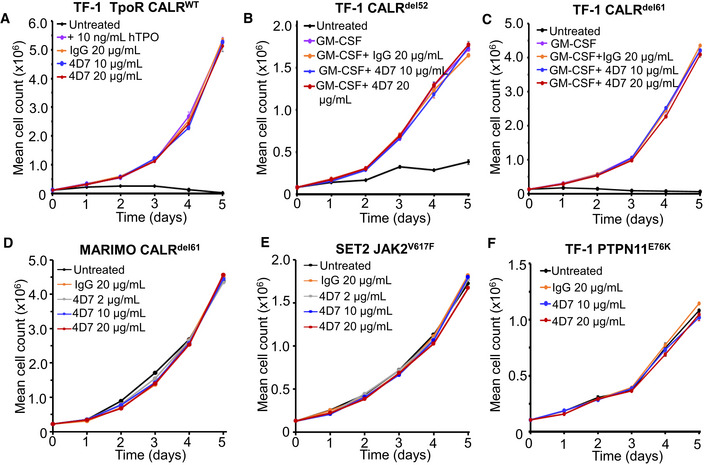
Biological specificity of 4D7 Cytokine‐dependent TF‐1 TpoR cells with an overexpression of WT CALR cultured in the absence of TPO, 10 ng/ml hTPO and 10 or 20 µg/ml 4D7 or 20 µg/ml control IgG antibody for 5 days and the number of trypan blue‐negative cells were counted every 24 h (*n* = 3 biological replicates with three technical replicates).Cytokine‐dependent TF‐1 CALR^del52^ cells lacking TpoR were cultured in the presence of 2 ng/ml GM‐CSF and 10 or 20 µg/ml 4D7 or 20 µg/ml control IgG antibody (*n* = 3 biological replicates with three technical replicates).Cytokine‐dependent TF‐1 CALR^del61^ cells lacking TpoR were cultured in the presence of 2 ng/ml GM‐CSF and 10 or 20 µg/ml 4D7 or 20 µg/ml control IgG antibody (*n* = 3 biological replicates with three technical replicates).MARIMO cells from which the CALR^del61^ mutation was originally amplified were cultured in the presence of 2, 10 or 20 µg/ml 4D7 or 20 µg/ml control IgG antibody (*n* = 3 biological replicates with three technical replicates).Cytokine‐independent SET2 cells which harbour the pathogenic JAK2^V617F^ mutation were cultured in the presence of 2, 10 or 20 µg/ml 4D7 or 20 µg/ml control IgG antibody (*n* = 3 biological replicates with three technical replicates).Cytokine‐independent TF‐1 PTPN11^E76K^ cells were cultured in the presence of 10 or 20 µg/ml 4D7 or 20 µg/ml control IgG antibody (*n* = 3 biological replicates with three technical replicates). Data information: For all panels, bars represent standard error of the mean.

We then tested cytokine‐independent TF‐1 TpoR CALR^del61^ and TF‐1 TpoR CALR^del52^ cells for their ability to proliferate after treatment with increasing concentrations of 4D7 antibody or isotype control. Firstly, we noted no inhibition by 4D7 of TF‐1 cells expressing TpoR alone (with endogenous wild‐type CALR; Fig 1E). In contrast, we observed blockade of TPO‐independent cell growth over 5 days using 2, 10 and 20 µg/ml 4D7 in cytokine‐independent cells (Fig 1F and G) with evidence of concentration‐dependent inhibition observed after 48 h. No inhibition was observed with IgG isotype control antibody at 20 µg/ml. No inhibition by 4D7 was observed in TF‐1 cells with an overexpression of wild‐type CALR (Fig EV2A). Additionally, TF‐1 cells harbouring either the *CALR* ^del52^ or *CALR* ^del61^ mutations but lacking TpoR were unaffected when treated with 4D7 (Fig EV2B and C). Interestingly, MARIMO cells also did not show inhibition with 4D7, consistent with *CALR* no longer acting as a driver mutation in this *NRAS* mutant cell line (Han *et al*, 2018) (Fig EV2D). In addition, no inhibition was observed in factor‐independent *JAK2* ^V617F^ SET2 cells (Fig EV2E) or factor‐independent TF‐1 expressing the *PTPN11* ^E76K^ mutation (Fig EV2F) indicating a mutation‐specific effect.

**Figure 2:**
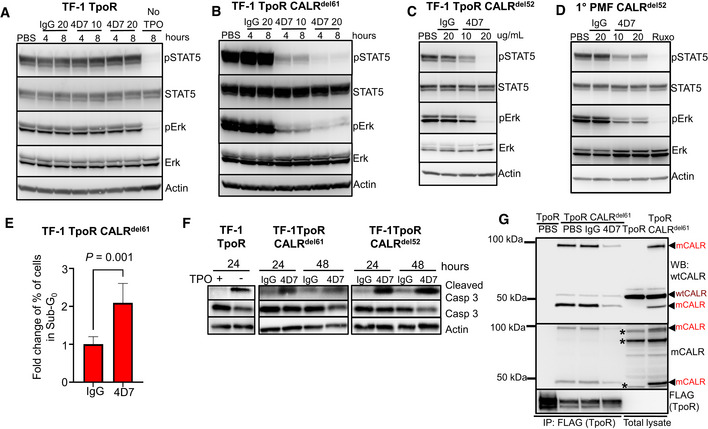
4D7 disrupts interaction with TpoR and downstream signalling Cell extracts blotted for phospho‐STAT5, total STAT5, phospho‐ERK, total ERK and actin from TF‐1 TpoR cells after incubation with 10 or 20 µg/ml 4D7 or IgG for 4 or 8 h, in presence of TPO. The last line indicates TPO withdrawal.Similar experiment using TPO‐independent TF‐1 TpoR CALR^del61^ cells.Similar experiment using TPO‐independent TF‐1 TpoR CALR^del52^ cells at 8 h.Similar experiment using PBMNCs from CALR^del52^ PMF primary cells at 8 h. Additionally, cells were treated with 280 nM of ruxolitinib as a positive control.Fraction of apoptotic sub‐G_0_ population of TF‐1 TpoR CALR^del61^ cells after 48 h of 4D7 or IgG treatment (*n* = 3 biological replicates, bars represent standard deviation for three replicates, normalized to IgG, with a Student's unpaired *t*‐test used to determine statistical significance).Western blot showing caspase 3 cleavage occurring within 24 h of TPO withdrawal in TPO‐dependent TF‐1 TpoR cells. An increase in cleaved caspase 3 is observed after 48 h of treatment with 20 µg/ml 4D7 in TF‐1 TpoR CALR^del61^ and TF‐1 TpoR CALR^del52^ cells.Western blot of TpoR immunoprecipitation under non‐reducing conditions showing associated CALR 50 kDa monomers and 100 kDa dimers (red arrowheads) present only in TF‐1 TpoR CALR^del61^ disrupted by 8‐h treatment with 20 µg/ml 4D7 but not PBS or 20 µg/ml IgG. CALR monomers and dimers are detectable by polyclonal anti‐wild‐type CALR or anti‐mutant CALR monoclonal antibodies. Red arrowheads, detected mutant CALR protein; brown arrowheads, detected wild‐type CALR protein; asterisk, non‐specific bands. Source data are available online for this figure.

**Figure EV3:**
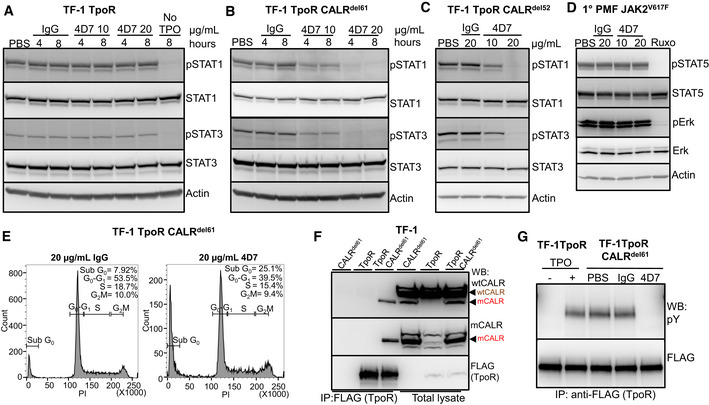
Monoclonal antibody blocks STAT1, 3 signalling and TpoR phosphorylation Cell extracts blotted for phospho‐STAT1, total STAT1, phospho‐STAT3, total STAT3 and actin from TF‐1 TpoR cells after incubation with 10 or 20 µg/ml 4D7 or IgG for 4 or 8 h as indicated.Similar experiment using TPO‐independent TF‐1 TpoR CALR^del61^ cells at 4 and 8 h.Similar experiment shown using TPO‐independent TF‐1 TpoR CALR^del52^ cells, 8 h.Similar experiment using PBMNCs from JAK2^V617F^ PMF primary cells at 8 h. Additionally, cells were treated with 280 nM of ruxolitinib as a positive control.Flow cytometry analysis for cell cycle distribution of TF‐1 TpoR CALR^del61^ cells exposed to 20 µg/ml IgG or 4D7 for 48 h. Cells were harvested and fixed and stained with propidium iodide and their DNA contents were analysed. Results from one representative experiment shown. Percentages of cells in Sub G_0_, G_1_, S and G_2_/M cycle indicated.Western blot showing specific co‐immunoprecipitation of mutant CALR^del61^ cells with TpoR anti‐FLAG antibody under reducing conditions detected by WT and mutant‐specific CALR antibodies. Red arrowheads, detected mutant CALR protein; brown arrowheads, detected wild‐type CALR protein.Western blot showing decreased TpoR phosphorylation in a TpoR immunoprecipitated after 8 h of 20 µg/ml 4D7 treatment compared to PBS or IgG control in TF‐1 TpoR CALR^del61^. Source data are available online for this figure.

To understand the mechanism of action of 4D7, we performed signalling experiments in TF‐1 cells. Firstly, we observed no inhibition of signalling in cytokine‐dependent TF‐1 cells expressing TpoR alone, which showed the expected down‐regulation of phospho‐STAT1,3,5 and phospho‐ERK only after TPO withdrawal (Figs 2A and EV3A). In contrast, we observed blockade of constitutive factor‐independent phospho‐STAT1/3/5 and phospho‐ERK after incubation with 10 or 20 µg/ml 4D7, but not with IgG control in both CALR^del61^ and CALR^del52^ cells (Figs 2B and C, EV3B and C). Importantly, this blockade was also observed in peripheral blood mononuclear cells (PBMNCs) from a primary CALR^del52^ PMF (Fig 2D) but not in JAK2^V617F^ PMF (Fig EV3D). Treatment with 4D7 resulted in a significant reduction in phospho‐signalling, whereas ruxolitinib resulted in complete suppression. This was expected as ruxolitinib is known to inhibit all JAK‐dependent signalling, lacking mutation specificity (Tvorogov *et al*, 2018). A significant increase in the sub‐G_0_ fraction was observed by 4D7 compared to IgG control (*P* = 0.001; Fig 2E) in the absence of a major effect on G_2_‐M on cell cycle analysis (Fig EV3E) consistent with induction of an apoptotic response. This was confirmed by caspase 3 cleavage that occurred within 24 h and peaked at 48 h after 4D7 treatment (Fig 2F).

### 4D7 disrupts the mutCALR/TpoR complex

**Figure EV4:**
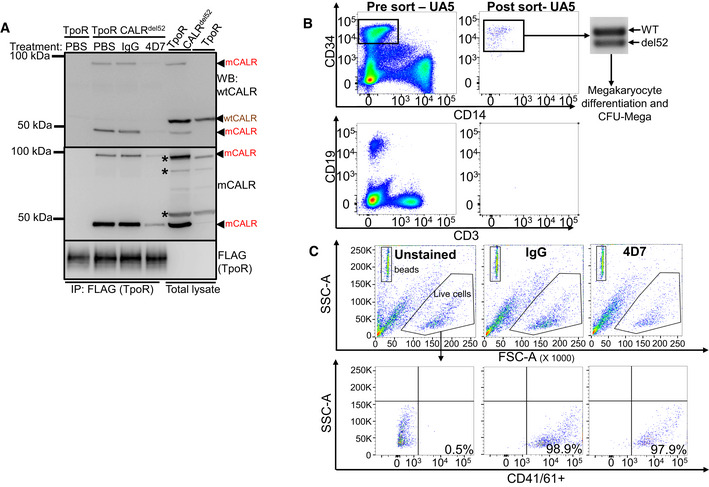
4D7 blocks CALR interaction with TpoR in CALR^del52^ cells Western blot of TpoR immunoprecipitation under non‐reducing conditions showing associated CALR 50 kDa monomers and 100 kDa dimers (red arrowheads) present only in TF‐1 TpoR CALR^del52^ disrupted by 8‐h treatment with 20 µg/ml 4D7 but not PBS or 20 µg/ml IgG. CALR monomers and dimers are detectable by polyclonal anti‐wild‐type CALR or anti‐mutant CALR monoclonal antibodies. Red arrowheads, detected mutant CALR protein; brown arrowheads, detected wild‐type CALR protein; asterisk, non‐specific bands.Peripheral blood mononuclear cells from PMF samples were thawed and stained for CD34, CD14, CD19 and CD3 prior to FACS sorting. Each population was collected and purity was verified prior to proceeding with any further analysis. PCR amplification of CALR exon 9 was carried out to confirm mutational status of CD34^+^ cells which were utilized in megakaryocyte differentiation assays and colony forming assays in the presence of 4D7 or IgG.Representative flow cytometry plots for determination of CD41^+^/61^+^ populations from liquid culture assay from one PMF patient. Beads shown in the upper left panel with high SSC‐A. Live cell population shown in hexagon gate (top panel). CD41/61^+^ population gates shown in lower panel with % CD41/61^+^ cells indicated for unstained, IgG‐ and 4D7‐treated cells over 12 days. The number of CD41/61^+^ cells‐to‐bead ratio used to enumerate effect of 4D7 on megakaryopoiesis. Source data are available online for this figure.

To investigate the mechanism of action of 4D7, we examined how it affects mutant CALR interactions with TpoR. Analysis of TF‐1 TpoR CALR^del61^ cells revealed that CALR mutants constitutively existed in complex with the TpoR (Fig EV3F) consistent with previous findings (Araki *et al*, 2019). Importantly, pre‐treatment with 4D7 diminished the presence of CALR monomers (slightly < 50 kDa) and dimers (~ 100 kDa) bound to TpoR (Fig 2G), supporting the notion that 4D7 interferes with the mutCALR/TpoR complexes at the cell surface by disrupting the constitutive association of mutCALR to TpoR. Similar results were obtained with TF‐1 TpoR CALR^del52^ cells (Fig EV4A). 4D7 also prevented TpoR activation in the presence of CALR‐mutant protein as demonstrated by its inhibition of TpoR tyrosine phosphorylation (Fig EV3G) and downstream signalling (Fig 2B–D), consistent with inhibition of receptor activation.

### Monoclonal antibody 4D7 inhibits TPO‐independent megakaryocyte formation

**Figure 3:**
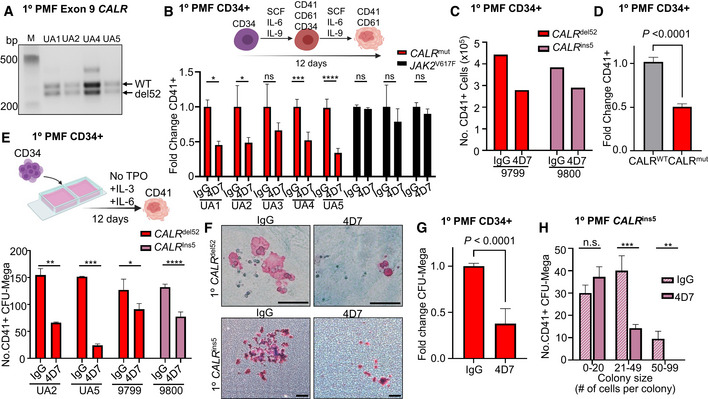
4D7 monoclonal antibody specifically inhibits primary megakaryocyte differentiation of mutated *CALR* myelofibrosis samples PCR amplification of *CALR* exon 9 from patients confirming heterozygous del52 mutation in sorted CD34^+^ cells obtained from *CALR*‐mutated myelofibrosis samples.Graph showing the decreased fold change of CD41^+^CD61^+^ megakaryocytes cultured in 4D7 compared to IgG. FACS‐sorted CD34^+^ from myelofibrosis patients with *CALR* or *JAK2* mutation was cultured over 12 days without TPO in the presence of SCF, IL‐6 and IL‐9. Black columns show *JAK2* ^V617F^ mutation‐positive samples (*n* = 4 technical replicates of samples from different patients).Number of CD41^+^ megakaryocytes derived from isolated CD34^+^ progenitors from myelofibrosis patients. Number of CD41/CD61^+^ cells counted on day 12 using trypan blue exclusion (*n* = 1 technical replicate).Summary of fold change reduction of CD41/CD61^+^ megakaryocytes by 4D7 in all tested *CALR*‐mutated patient samples compared to *CALR* wild type, normalized to IgG (*n* = 11 samples from different patients, with four technical replicates per sample).Number of CD41^+^ megakaryocyte colonies from patient samples after 4D7 treatment. CD34^+^ from patients with myelofibrosis with *CALR* ^del52^ or *CALR* ^ins5^ was plated on collagen‐based matrix in presence of 20 µg/ml 4D7 or IgG control (*n* = 3 biological replicates).Representative micrographs showing CD41^+^ colonies in pink and CD41‐ colonies in blue after treatment with 4D7 or IgG at 100× or 40× magnification. Scale bar indicates 100 µm.Summary of fold change reduction in CD41^+^ megakaryocytes in *CALR*‐mutated samples cultured MegaCult treated with 4D7 compared to IgG (*n* = 4 biological replicates).Number of megakaryocyte colony forming units grouped according to colony cell number after 4D7 treatment in a mutant *CALR* ^ins5^ sample (*n* = 3 biological replicates). Data information: Bars represent standard error of means in (D) and (G). Unpaired Student's *t*‐test used to determine statistical significance in (B, D, E, G and H). Bars represent standard deviations in (B, E and H). *P = 0.05–0.01, **P = 0.01–0.001, ***P = 0.001–0.0001, ****P < 0.0001, n.s, not significant.

Primary myelofibrosis is characterized by abnormal proliferation and morphology of clonal megakaryocytes. To examine whether 4D7 suppressed CALR‐mutant megakaryocytes, we tested its activity on purified primary CD34^+^ cells obtained from patients with *CALR*‐mutant myelofibrosis using two orthogonal assays: (i) TPO‐independent megakaryocyte differentiation in liquid culture, and (ii) TPO‐independent megakaryocyte colony formation on a collagen‐based medium. Five of eight patients had the most common type 1 (52 base pair deletion) *CALR* mutation which was heterozygous in the bulk of the CD34^+^ fraction (Fig 3A). The sorting strategy for myelofibrosis stem cells is shown in Fig EV4B and clinical details for all patients are listed in Table EV1. Strikingly, four of five mutant *CALR* MF patient samples that displayed robust TPO‐independent growth of CD41^+^CD61^+^ megakaryocyte progenitors showed inhibition by 4D7 of at least 50% (Figs 3B and EV4C). Interestingly, the patient sample exhibiting less inhibition harboured a 34 base pair deletion. No significant inhibition was observed when *JAK2* ^V617F^ was present in all three myelofibrosis samples (black bars; Fig 3B). An additional two patient samples obtained from the University of Graz also showed evidence of inhibition compared to IgG control, including a type 2 *CALR* patient (five base pair insertion; Fig 3C). Overall, a mean decrease of 55% was observed across *CALR*‐mutant samples after 4D7 treatment (*P* < 0.0001; Fig 3D). Similarly, we saw a dramatic reduction in the absolute numbers of primary TPO‐independent megakaryocyte colonies cultured on collagen (colony‐forming unit‐mega) treated with 4D7 in multiple patient samples (decrease of 62%, *P* < 0.0001; Fig 3E–G). Strikingly, the size of 4D7‐treated megakaryocyte colonies was substantially smaller as enumerated in a patient with a type 2 mutation (Fig 3H).

### Monoclonal antibody 4D7 inhibits the growth of ruxolitinib‐persistent cells

**Figure 4:**
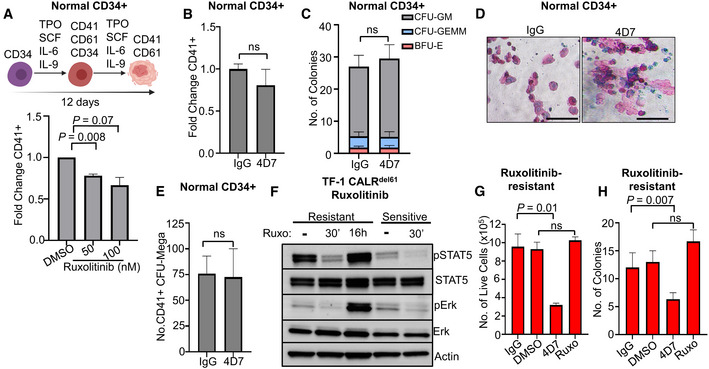
4D7 has no effect on normal progenitor cells and shows activity against ruxolitinib‐resistant cells without haematological toxicity Graph showing effect of ruxolitinib on megakaryocyte differentiation at 50 and 100 nM in healthy CD34^+^ cells (*n* = 4 different cords collected with three technical replicates).Effect of 4D7 on TPO‐dependent megakaryocyte differentiation of healthy CD34^+^ cells cultured with 20 µg/ml 4D7 or IgG control (*n* = 2 biological replicates, with two technical replicates).Total numbers of haematopoietic colonies plated in MethoCult from healthy cord blood after treatment with 4D7 or IgG. CFU‐GM, colony forming unit–granulocyte macrophage; BFU‐E, blast forming unit–erythroid; CFU‐GEMM, colony forming unit–granulocyte, erythroid, monocyte and megakaryocyte (*n* = 3 biological replicates).CD41^+^ megakaryocyte colonies from healthy CD34^+^ cells observed on collagen matrix after 4D7 treatment. Colonies were cultured in TPO, SCF, IL‐9 and IL‐3. Scale bar indicates 100 µm.Number of megakaryocyte colony forming units observed in MegaCult assay after treatment of healthy cord blood CD34^+^ cells with either IgG or 4D7 (*n* = 3 biological replicates).Western blot showing signalling in ruxolitinib‐resistant TF‐1 TpoR CALR^del61^ compared to ruxolitinib‐sensitive TF‐1 TpoR CALR^del61^ cells after treatment with 100 nM ruxolitinib for 16 h or 30 min and blotted for phospho‐STAT5, total STAT5, phospho‐ERK, total ERK and actin as indicated. Ruxolitinib‐sensitive cells were non‐viable after 16 h of treatment.Comparison of cell growth after DMSO, 20 µg/ml 4D7, IgG or 100 nM ruxolitinib treatment over 4 days of ruxolitinib‐resistant TF‐1 TpoR CALR^del61^. Cells counted using trypan blue exclusion (*n* = 3 biological replicates with three technical replicates).Number of colonies observed from cells plated in MethoCult following 72 h of treatment with DMSO, 20 µg/ml 4D7, IgG or 100 nM ruxolitinib performed in ruxolitinib‐resistant TF‐1 TpoR CALR^del61^ cells (*n* = 3 biological replicates with three technical replicates). Data information: Error bars on (A, B, C and E) indicate standard error of mean. Error bars on (G and H) represent standard deviation. All *P*‐values are unpaired Student's unpaired *t*‐test. Source data are available online for this figure.

Ongoing follow‐up of COMFORT‐1 and COMFORT‐2 studies (Harrison *et al*, 2012; Verstovsek *et al*, 2012) suggests patients with *CALR* mutations are less responsive to the JAK inhibitor ruxolitinib than patients with *JAK2* mutations, but cytopenia limits the use of higher treatment doses (Ross *et al*, 2021). We therefore tested whether an immunotherapeutic approach would have efficacy in cells with ruxolitinib “persistence/resistance” (Meyer *et al*, 2015). We first showed that ruxolitinib had inhibitory effects on megakaryocyte formation from healthy cord blood with > 30% inhibition after 12 days at 50 or 100 nM (*P* = 0.008 and 0.07, respectively; Fig 4A). In contrast, 4D7 had no effect on normal haematopoiesis. At the highest concentration, 4D7 did not inhibit physiological megakaryopoiesis in liquid culture (Fig 4B), haematopoietic granulocyte‐macrophage colony formation and erythroid colony formation (Fig 4C), or collagen‐plated megakaryocyte colony numbers (CFU‐Mega; Fig 4D and E) compared to IgG control.

To generate ruxolitinib‐resistant cells, we cultured TF‐1 TpoR CALR^del61^ cells with increasing concentrations of ruxolitinib over 4 weeks, up to 100 nM. Ruxolitinib‐resistant cells showed enhanced phospho‐STAT5 and phospho‐ERK phosphorylation in the presence of ruxolitinib but still showed some inhibition after withdrawal and re‐stimulation (Fig 4F). Importantly, 4D7 had strong inhibitory activity on cells that were resistant to ruxolitinib, in both liquid culture at 96 h (Fig 4G) and in colony formation (Fig 4H). Together, these results suggest that an immunotherapeutic approach with 4D7 may have clinical utility in *CALR*‐mutant patients who develop resistance/persistence of myelofibrosis during ruxolitinib treatment.

**Figure EV5:**
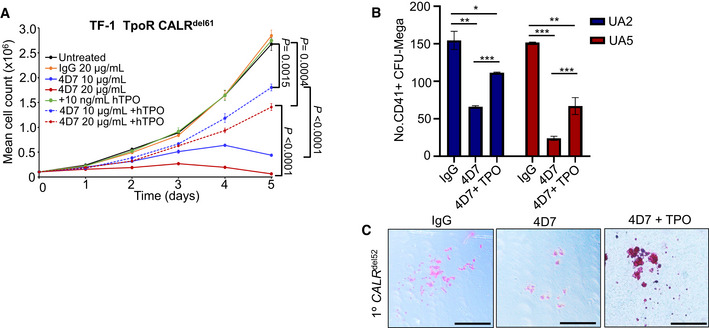
Effect of 4D7 antibody on TPO receptor biology Proliferation curves of factor‐independent TF‐1 TpoR CALR^del61^ cells cultured with 10 or 20 µg/ml 4D7 or 20 µg/ml of control IgG antibody in presence or absence of 10 ng/ml TPO (*n* = 3 biological replicates with three technical replicates).Number of CD41^+^ megakaryocyte colonies CALRdel52 patient after 4D7 treatment in the presence of TPO. Samples were seeded in a collagen‐based matrix in presence of 20 µg/ml 4D7 or IgG control with 50 ng/ml TPO (*n* = 2 patient samples with two technical replicates).Representative micrographs showing CD41^+^ colonies in pink and CD41‐ colonies in blue after treatment with IgG or 4D7 in absence or presence of 50 ng/ml TPO. Scale bar indicates 100 µm. Data information. Error bars represent standard error of the mean in (A) and standard deviation (B). Unpaired Student's *t*‐test used to determine statistical significance in (B). *P = 0.05–0.01, **P = 0.01–0.001, ***P = 0.001–0.0001.

A recent report suggests TpoR co‐expressed with mutant CALR may have a distinct conformation that is less responsive to physiological TPO compared to normal TpoR (Basso‐Valentina *et al*, 2021). We therefore performed rescue experiments with 10 ng/ml TPO in the presence of 4D7. We found in the presence of 4D7, TPO could only partially rescue the proliferation defect in both TF‐1 TpoR CALR^del61^ cell line and patient‐derived megakaryocyte colonies (Fig EV5A–C), suggesting endogenous TPO may not be sufficient to overcome 4D7 effect *in vivo*.

### The 4D7 monoclonal antibody blocks CALR‐dependent proliferation and prolongs survival in xenograft models

**Figure 5:**
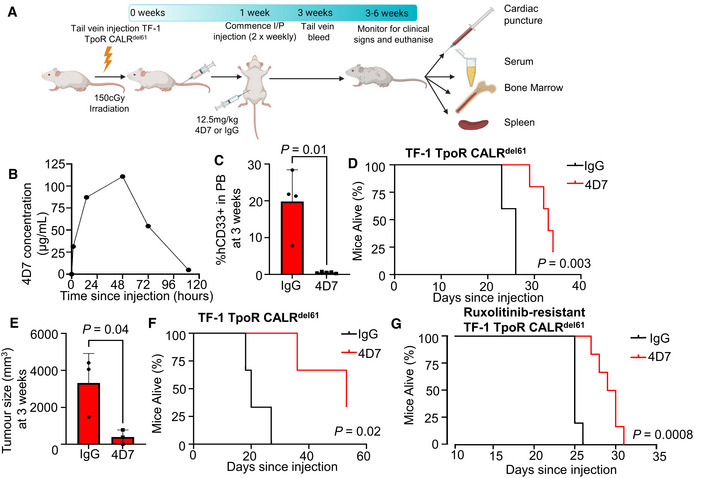
4D7 blocks mutant CALR‐dependent proliferation *in vivo* and prolongs survival Illustration showing bone marrow NSG engraftment model with TPO‐independent TF‐1 TpoR CALR^del61^ cells treated with 4D7 or IgG control twice weekly, starting 7 days after engraftment via intraperitoneal injection and final measurements taken from euthanized mice.Pharmacokinetic measurements of serum levels of 4D7 in mice after intraperitoneal injection at 0, 1, 24, 48, 72 and 110 h since administration.Percentage of TF‐1 TpoR CALR^del61^ human CD33^+^ cells measured in peripheral blood at 3 weeks post‐tail vein engraftment (*n* = 5 mice per treatment).Kaplan–Meier survival curve of bone marrow engraftment model of TF‐1 TpoR CALR^del61^ cells showing improved survival of mice treated with 4D7 compared with IgG control commencing 1 week after tail vein injection (*n* = 5 mice per treatment).Mean tumour volume at 21 days after subcutaneous injection of TF‐1 TpoR CALR^del61^ cells following treatment with 4D7 or IgG. Cells were pre‐treated with 4D7 or IgG control for 1 h prior to injection and treatment continued twice weekly at 12.5 mg/kg until euthanasia (*n* = 3 mice per treatment).Kaplan–Meier survival curve of TF‐1 TpoR CALR^del61^ chloroma mice treated with 4D7 or IgG until humane killing due to tumour diameter > 30 mm or ulceration (*n* = 3 mice per treatment).Kaplan–Meier survival curve of NSG mice engrafted with ruxolitinib‐resistant TF‐1 TpoR CALR^del61^ treated with 12.5 mg/kg 4D7 or IgG twice weekly (*n* = 5 and 6 mice for IgG and 4D7, respectively). Data information: For (C and E), a Student's unpaired *t*‐test was used to determine statistical significance. For all survival curves, the log‐rank Mantel–Cox test *P*‐value is shown.

Myeloproliferative neoplasms are fundamentally disorders of unregulated proliferation. To test whether 4D7 could block mutant *CALR*‐dependent proliferation *in vivo*, we developed two distinct cell‐line xenograft models. The first was a bone marrow engraftment model, which measures mutant *CALR*‐dependent proliferation of TF‐1 TpoR CALR^del61^ cells in the bone marrow microenvironment. The second was a chloroma model created by subcutaneous injection of TF‐1 TpoR CALR^del61^ cells into the flanks of NSG mice, which mimics extramedullary haematopoiesis. In both models, fibrosis is not an evaluable disease outcome. In the bone marrow engraftment model (Fig 5A), 4D7 treatment (12.5 mg/kg twice weekly via intraperitoneal injection starting day 7) showed an excellent pharmacokinetic profile, achieving a serum concentration of more than 100 µg/ml 48 h post‐injection (Fig 5B), lowered peripheral blood engraftment of human CD33^+^ cells at 3 weeks (0.04 vs. 19.8% CD33^+^ 4D7 vs. IgG, *P* = 0.01; Fig 5C) and significantly prolonged survival (log‐rank hazard ratio 0.24, *P* = 0.003; Fig 5D). In the chloroma model, 4D7 treatment significantly slowed tumour growth at 21 days post‐engraftment (353 vs. 3,317 mm^3^ mean tumour volume, 4D7 vs. IgG, *P* = 0.04; Fig 5E) and prolonged survival (hazard ratio 0.19, *P* = 0.02; Fig 5F). Strikingly, mutant CALR cells induced to be resistant to 100 nM ruxolitinib also showed a survival advantage after treatment with 4D7 (12.5 mg/kg twice weekly beginning at day 7) in the bone marrow engraftment model (hazard ratio 0.26, *P* = 0.0008; Fig 5G). Together, these results suggest an immunotherapeutic approach with antibody 4D7 may have clinical utility in *CALR*‐driven MPNs as well as in *CALR*‐mutant patients who develop resistance/persistence to ruxolitinib.

Primary myelofibrosis is an insidious and poorly understood disorder that encompasses features of both cancer and chronic inflammation. It is a clonal neoplasm driven by a handful of somatic mutations that activate cell signalling, presumably residing in the long‐term stem cell compartment (Wernig *et al*, 2006; Nangalia *et al*, 2013; Reinisch *et al*, 2016), which also has a constellation of cytokine‐mediated symptoms that are disproportionately severe. After the discovery of activating *JAK2* mutations in 50–60% of patients with PMF (Baxter *et al*, 2005; Kralovics *et al*, 2005), clinicians were hopeful that myelofibrosis would respond to tyrosine kinase inhibitor therapy in a similar fashion to chronic myeloid leukaemia. However, maturing data from the COMFORT studies and other trials have shown ruxolitinib also does not cause widespread regression of fibrosis or eradicate disease clones in a mutation‐specific manner (Verstovsek *et al*, 2012; Cervantes *et al*, 2013; Harrison *et al*, 2016). Exceptional cases of complete haematological or molecular remission have been reported (Masarova *et al*, 2016), but are uncommon. Typically, there is a gradual loss of response over time, with only 27% of patients remaining on first‐line ruxolitinib treatment after 5 years in the COMFORT studies, underscoring a need for alternative approaches.

Somatic mutations in exon 9 of the endoplasmic chaperone protein CALR are found in 70–80% of patients with *JAK2*‐negative PMF, accounting for ~ 30% of PMF cases overall, and also present in a similar proportion of essential thrombocythemia (Klampfl *et al*, 2013; Nangalia *et al*, 2013). In a subsequent analysis of patients from the COMFORT‐2 study, the response rate to ruxolitinib was 20% (confidence interval 5.7–43.7%) in patients with a *CALR* mutation vs. 34% (24.8–44.1%) in *CALR*‐negative patients, most of whom had a *JAK2* V617F mutation (Guglielmelli *et al*, 2016), suggesting that *CALR*‐mutant myelofibrosis may be less responsive to JAK inhibitor therapy. Although more than 50 *CALR* mutations have been described, the majority can be classified as type 1 like or type 2 like based on predicted protein structures (Eder‐Azanza *et al*, 2014). Our data suggest that both type 1 and type 2 *CALR* mutations could be amenable to an immunotherapeutic approach.

Here, we show for the first time that primary cells from patients with *CALR* mutations can be targeted with a mutation selective immunotherapy. Our data confirm the observations from other studies using human cells that mutant CALR protein is secreted and accessible on the cell surface and can drive growth factor independence, provided TpoR is co‐expressed. Data from independent laboratories have shown that mutant CALR protein requires TpoR for signalling and factor‐independent cell growth, and that the normal lectin domain of CALR is essential to bind glycosylated sites on TpoR (Elf *et al*, 2016, 2018; Pecquet *et al*, 2019). The relative contribution of autocrine vs. paracrine vs. endosomal signalling of mutant CALR protein has not been fully examined, with most studies to date performed in murine IL‐3‐dependent Ba/F3 cells. In our human TF‐1 model of mutant CALR‐induced proliferation, no evidence of a paracrine effect could be demonstrated in a horizontal co‐culturing assay (Fig EV1D). Remarkably, despite a demonstrated lack of autocrine/paracrine signalling, mutant CALR protein appears to be exquisitely sensitive to 4D7 binding in a dose‐dependent manner with concomitant inhibition of cell proliferation. Our results indicate that the CALR‐TpoR complex can be disrupted via a monoclonal antibody, and specifically we identified evidence for mutCALR bound constitutively to TpoR that could be inhibited by 4D7, thus blocking TpoR phosphorylation as well as downstream STAT and ERK signalling in primary samples. Future studies should determine the 3D structure of 4D7 bound to CALR and help test whether TPO analogues can overcome the effects that we describe (Basso‐Valentina *et al*, 2021).

Recently, it was reported that mutant CALR protein is present at high levels in the plasma of myelofibrosis patients (compared to normal individuals) (Sollazzo *et al*, 2016; Pecquet *et al*, 2018). The cell surface expression of mutant CALR is an ideal target for immunological therapies. It is present on the cell surface (Pecquet *et al*, 2018; Balligand *et al*, 2020; Masubuchi *et al*, 2020) and contains sequences not normally present in healthy mammalian cells or conserved across mammalian species. *CALR* mutations appear to be an early event in MPN ontogeny (perhaps only preceded by *TET2* in some cases). This results in it being present in all of the cells of the clone, unlike *JAK2* ^V617F^ which can be either an initiating or a secondary lesion, and found in only around half of patients with AML arising from an antecedent *JAK2* ^V617F^ MPN (Ross *et al*, 2021).

The development of a therapeutic antibody directed against mutant CALR protein has enormous clinical potential. The mouse model utilized within this study shows safety and efficacy for a rapidly proliferating tumour that is mutant CALR dependent, however, is not a relevant disease model for MPN. Future studies should investigate efficacy in relevant heterozygous mouse models (Li *et al*, 2018; Balligand *et al*, 2020; Achyutuni *et al*, 2021), including evidence for fibrosis reversal. Additionally, the antibody that we have developed against the common neoepitope C‐terminus of CALR can be used in both insertion and deletion *CALR* mutation‐positive patients and appears to have minimal or no effects on normal cells *ex vivo*. The safety and tolerability of this antibody therapy could lead to its application in the earlier stages of PMF to prevent clinical progression and the acquisition of clonal heterogeneity. In parallel, an anti‐mutant CALR antibody such as 4D7 could also be combined with other treatments with the aim of augmenting their effect, as has been successfully applied in B‐cell lymphoma using anti‐CD20 antibodies (Czuczman *et al*, 1999). Future studies should examine a similar approach in patients with essential thrombocythemia with *CALR* mutations and secondary acute myeloid leukaemia that has transformed from *CALR*‐mutant myeloproliferative disorders.

Myelofibrosis can be associated with immune defects which are compounded by the effects of JAK inhibitors like ruxolitinib. There is, therefore, an argument in favour of immunotherapeutics that can be used in the less advanced stages of myeloproliferative neoplasms. Although immunodeficiency may limit the potential of antibody therapeutics that require intact complement‐mediated cytotoxicity, this can be overcome by the addition of toxin conjugates or the use of chimeric antigen receptor T cells. Importantly, our data suggest that antibody‐mediated inhibition of cell signalling may also contribute to suppression of CALR‐mutant cell proliferation, independent of any immune‐mediated effects. Physiologically, the SIRP alpha protein is normally present as a ligand for the “don't eat me” CD47 signal protecting cells from phagocytosis (Majeti *et al*, 2009; Majeti, 2011; Liu *et al*, 2020) but inhibition of macrophage phagocytosis does not appear to be involved in the disease progression of MPN (Daitoku *et al*, 2016). It will be intriguing to test anti‐CALR monoclonal antibody efficacy in the setting of macrophage activation or CAR T‐cell therapies.

## Materials and Methods

### Cell lines

TF‐1 cells were cultured in RPMI with 10% (v/v) foetal calf serum (FCS) supplemented with 2 ng/ml of GM‐CSF and 25 mM HEPES. HEK293T cells were cultured in DMEM with 10% FCS. TF‐1 TpoR cells were cultured in RPMI with 10% FCS supplemented with 10 ng/ml of human TPO (Peprotech). MARIMO and SET2 cells were cultured in RPMI with 10% FCS.

### CALR mutation cloning

Human *CALR* (del61) with a 61 base pair deletion in *CALR* exon 9 was PCR amplified from MARIMO cDNA with oligonucleotide primers 5′‐TAT AGA ATT CGC CAC CAT GCT GCT ATC CGT GCC G‐3′ and 5′‐TAT AGA ATT CAG GCC TCA GTC CAG CCC T‐3′. The resultant PCR product was cloned into pCX4‐IRES eGFP (Neubauer *et al*, 2016) following digestion with EcoRI. Sequencing verified integrity and orientation of the cDNA. CALR^del61^ protein was expressed in TF‐1 TpoR. This cell line was designated as TF‐1 TpoR CALR^del61^ and was shown to obtain cytokine independence.

### Rat monoclonal antibody production

Rats were immunized with a CALR‐mutant peptide “MMRTKMRMRRMRRT RRKMRRKMSPARPRTS” coupled to KLH, this peptide sequence is unique to CALR mutant. Serum from the immunized rats was screened by enzyme‐linked immunoassay, to verify a strong titre to the peptide immunogen, prior to performing the hybridoma fusion. Immunized rat spleen cells were harvested and a spleen single‐cell suspension was combined with Sp2/O myeloma cell line. The cell mixture was treated with polyethylene glycol plus DMEM prior to being plated in 96‐well flat bottom plates. The expanding hybridomas were screened initially by ELISA and the positive clones were further verified by western blot for reactivity to the expressed CALR‐mutant protein before subcloning the hybridomas for antibody production.

### Lentiviral expression constructs

Human CALR and PTPN11 vectors were produced using a codon‐optimized coding sequence encoding CALR^WT^, CALR^del52^ or PTPN11^E76K^ mutations purchased as gBlocks from Integrated DNA Technologies (Iowa, USA). Mutations were cloned into pLVX‐eF1α‐IRES‐ZsGreen1 lentiviral expression construct (Takara Bio Inc., Shiga, Japan). For virus production, HEK293T cells were seeded at 3 × 10^6^ cells per 75 cm^2^ flask 40 h prior to transfection. Each flask was transfected using Lipofectamine 2000 with 13.5 µg of CALR WT or ‐mutant pLVX vectors together with 13 µg envelope plasmids, VsVg and PAX2. Viral supernatants were collected 48 h after transfection. To establish cell lines expressing various *CALR* constructs, viral supernatants were applied to RetroNectin‐coated (Takara) tissue culture plates and spun at 2,000 *g* for 2 h at 37°C. Cell lines were applied to virus and spun at 200 *g* and cultured in the presence of virus for 24 h. ZsGreen1‐positive cells were sorted using the FACS Melody (BD Biosciences).

### Antibody ^125^Iodine‐labelling and Scatchard analysis

4D7 was radio‐iodinated with^125^I (Perkin‐Elmer) using Pierce Pre‐Coated Iodination tubes (Thermo Scientific) with an estimated specific activity of 7,733 cpm/ng. For saturation‐binding assays, plates were coated overnight at 4°C with full‐length neoepitope CALR peptide (2 µg/ml) or control scrambled peptide (2 µg/ml). Wells were washed three times with phosphate‐buffered saline + 0.05% Tween20, blocked with 5% bovine serum albumin for 1 h followed by further washing.^125^I‐4D7 was added at concentrations ranging from 2 pM to 2.5 nM in triplicate and incubated at room temperature for 2 h. After washing, 1 M HCl was added for 30 min to elute bound antibody. Two hundred microliter aliquots from each well were counted on a PerkinElmer 2470 Wizard2 Automatic Gamma Counter. Dissociation constants were calculated using the EBDA and LIGAND programmes.

### Cell proliferation to assess 4D7 effect on various cell lines

In brief, cells were seeded at 5 × 10^4^/ml in standard growth media, with any necessary cytokines, and the addition of 20 µg/ml IgG or 2, 10 or 20 µg/ml 4D7. Cells were seeded in triplicate and each well was counted in triplicate every 24 h for 5 days using trypan blue exclusion.

### Primary myelofibrosis stem cell experiments

Umbilical cord blood (UCB) was collected with written consent from full‐term deliveries at the Women's Health Unit, Lyell McEwin Hospital (Adelaide, South Australia), or the Department of Obstetrics and Gynaecology, Medical University of Graz (Austria), with institutional review board approval (IRB approval: 31‐322 ex 18/19; HREC/20/WCHN/65). Samples were processed using a Ficoll‐Paque (GE Healthcare) density gradient to isolate mononuclear cells (800 *g*, room temperature, 30 min, deceleration off), followed by red cell lysis (ammonium–chloride–potassium lysing buffer) to remove remaining red blood cells. CD34 enrichment was performed by magnetic cell separation using CD34 Microbead kit (Miltenyi Biotech). Alternatively, purified CD34^+^ cells from cord blood were purchased from Lonza. CD34^+^ cells with a purity above 90% were either cryopreserved or directly cultured in serum‐free stem cell retention media (StemSpan SFEMII, Stemcell Technologies, Vancouver, BC, Canada) supplemented with human recombinant SCF, TPO, FLT3, IL‐6 (all 20 ng/ml), UM729 (1.75 µM) and StemReginin 1 (SR1, 300 nM). All cytokines and SR1 were purchased from Peprotech (Rocky Hill, NJ, USA), whereas UM729 was purchased from Stemcell Technologies.

### PMF patient samples

Patient samples were collected with informed consent from the South Australian Cancer Research Biobank (SACRB; Adelaide, Australia). Primary CD34^+^ stem/progenitor cells from peripheral blood samples from patients with JAK2^V617F^ or *CALR*‐mutant myelofibrosis were purified by flow cytometry using CD34‐APC antibody, anti‐human CD45RA Brilliant Violet 605, anti‐CD90 FITC, anti‐human CD123‐PE, anti‐human CD38 PE‐Cy7, or with CD34‐APC antibody, anti‐human CD3 FITC, anti‐human CD19‐PE (clone H1B19) and anti‐human CD14 PE‐Cy7.

### Mutational screening of patient samples

DNA was extracted from primary PMF samples using QuickExtract^TM^ (Epicentre Biotechnologies) following the manufacturers protocol. PCR reactions were set up in a final concentration of 1X PCR buffer, 1.5 mM MgCl_2_, 0.2 mM dNTPs, 0.2 µM forward and reverse primers (CALR‐F; 5′‐TAA CAA AGG TGA GGC CTG GT‐3′, CALR‐R; 5′ GCC TCT CTA CAG CTC GTC CTT‐3′), 0.5 units Taq Polymerase (Promega) and 50 ng genomic DNA in a final volume of 25 µl. Reactions were undertaken in a T100 Thermocycler (BioRad). Samples were activated/denatured at 95°C for 10 min, then were cycled at 95°C for 30 s, 58°C for 30 s and 72°C for 1 min for 40 cycles, followed by a 10‐min incubation at 72°C. Samples were visualized by electrophoresis on a 2% agarose gel containing 1 X Gel Red (Biotium) and imaged on the BioRad GelDoc XR^+^ (BioRad).

### Haematopoietic colony formation

For megakaryocyte differentiation assays, CD34^+^ cells derived from cord blood or from patient peripheral blood mononuclear fractions were plated in MegaCult (MegaCult™‐C Collagen and Medium with Lipids) with added IL‐3 and IL‐6 (10 ng/ml) in the presence or absence of 50 ng/ml TPO. Cells were plated at an initial density of 1,800 cells/well and cultured for 12 days at 37°C and 5% CO_2_. Primary PMF CD34^+^ cells were cultured in StemCell Pro supplemented with human recombinant SCF (25 ng/ml), IL‐6 and IL‐9 (10 ng/ml) to allow differentiation into megakaryocytes. CD34^+^ cord blood cells were cultured in 50 ng/ml SCF, 50 ng/ml TPO, 20 ng/ml IL‐6 and 20 ng/ml IL‐9. Both IgG and 4D7 were added at 20 µg/ml. All cytokines are from Peprotech. Culture media were replenished every 3 days and cells were assessed for megakaryocyte differentiation on day 12 using anti‐human CD41/61 PE‐Cy7 (Clone A2A9/6) and CountBright beads (Thermo Fisher) to enumerate cell numbers. CD34^+^ obtained from UCB was plated in MethoCult containing cytokines (H4434). Cells were seeded at approximately 100 cells per well and were cultured in the presence of 20 µg/ml 4D7 or control IgG antibody and scored for colony and blast forming units at 12–14 days.

### Flow cytometry assessment of 4D7 binding to the cell surface

TF‐1 cells expressing TpoR, CALR^WT^ and mutant CALR were incubated for 30 min 4D7 conjugated to phycoerythrin (PE; Abcam, #AB102918) or IgG2a PE isotype control (ThermoFisher #12‐4321‐42) at the concentrations specified after pre‐incubation with mouse serum. Cells were washed 1× before analysis on a CytoFLEX (Beckman Coulter).

### Analysis of cell cycle

Cell cycle analysis was carried out by flow cytometry. Briefly, TF‐1 TpoR and TF‐1 TpoR CALR^del61^ cells were seeded at a concentration of ~ 2 × 10^5^ per well in triplicate under standard culture conditions, with the addition of 20 µg/ml IgG or 4D7. Cells were treated for 48 h and were fixed by 70% (v/v) ethanol at 4°C overnight. Fixed cells were rinsed twice with PBS and stained with 50 µg/ml propidium iodide (PI; ThermoFisher) + 100 µg/ml RNAse A (QIAGEN). Cells were incubated for 30 min at RT in the dark and analysed on the BD FACSCantoII (BD Biosciences).

### Co‐culture of CALR‐secreting cells

In brief, TF‐1 TpoR and TF‐1 TpoR CALR^del61^ were seeded at the same density in opposing wells of a UniWells^TM^ Horizontal co‐culture plate (FujiFilm, #2501‐02FW) separated by a 0.75 µm filter. Cells were seeded in the presence or absence of 10 ng/ml TPO in triplicate and counted every day for 4 days by trypan blue exclusion. Representative images of wells were taken on day 4.

### Protein lysate preparation and Western blot analysis

For immunoblotting of total lysates, cells were lysed in NP40 buffer containing 150 mM NaCl, 50 mM Tris pH 7.6, 1% NP‐40, supplemented with protease inhibitors (Complete, Roche) and phosphatase inhibitor cocktails, and boiled for 5 min after addition of sample buffer (60 mM Tris pH 6.8, 5% glycerol, 1% SDS, 2% β‐mercaptoethanol, and 0.02% bromophenol blue) before SDS gel electrophoresis followed by western blotting (Tvorogov *et al*, 2010). Primary antibodies against pERK (#9101), pSTAT5 (#9359), ERK (#4695), actin (#4970), caspase 3 (#14220) and wild‐type CALR (#12238) were purchased from Cell Signalling. Primary antibodies against pSTAT1 (#612233), pSTAT3 (#612357), STAT1 (#610186), STAT3 (#610190), STAT5 (#610192) and anti‐phosphotyrosine 4G10 (#610012) were purchased from BD Biosciences. Mutant CALR monoclonal antibody CAL2 was purchased from Dianova (Hamburg, Germany). Immunoprecipitation of TpoR was performed in NP40 lysis buffer with additional 50 mM iodoacetamide to avoid any *de novo* disulphide bond formation post‐lysis. To reduce IgG background during immunoprecipitation, anti‐FLAG conjugated to magnetic beads (Sigma, #M8823) was used. For this same reason, anti‐FLAG‐HRP conjugated antibodies (Sigma, #A8592) were used in western blotting.

### Ruxolitinib‐resistant cells

Ruxolitinib‐resistant TF‐1 TpoR CALR^del61^ cells were established by exposing cells of untreated TF‐1 TpoR CALR^del61^ cells to increasing concentrations of ruxolitinib (Selleckchem) over a 4‐week period. Cells were initially treated with 10 nM and increased to 100 nM.

### Xenograft models of disease

All procedures were approved by the South Australian Health and Medical Research Animal Ethics Committee (Protocol SAM21‐018). *Bone marrow engraftment model:* 4‐ to 6‐week‐old NSG mice were X‐ray irradiated with 150 cGy and 5 × 10^5^ cells (TF‐1‐TpoR CALR^del61^) or 5 × 10^4^ cells (100 nM ruxolitinib‐resistant TF‐1 TpoR CALR^del61^) were intravenously injected into mice. Seven days after injection, mice were administered 12.5 mg/kg IgG or 4D7 by intraperitoneal injection twice weekly. Tail vein bleeds were taken at 3 weeks and analysed via flow cytometry to determine human leukaemia content in the peripheral blood. Leukemic cells were propidium iodide negative, mCD45.1 negative, hCD33^+^ and ZsGreen1^+^. Mice were monitored daily and euthanized once clinical symptoms were observed. At the time of euthanasia, cardiac puncture was performed to obtain both peripheral blood and serum for analysis. Additionally, bone marrow from both femurs, spine and spleens was analysed for leukaemic content as detailed above. *Chloroma model:* 4‐ to 6‐week‐old NSG mice were anaesthetized by isoflurane inhalation and shaved on the left back/flank and 1 × 10^7^ cells were injected into the mice. Cells were pre‐treated with 20 µg/ml IgG or 4D7 for 1 h prior to injection. Seven days after injection, mice were administered 12.5 mg/kg IgG or 4D7 by intraperitoneal injection twice weekly. Tumour progression was assessed through calliper measurements every 2 days. Mice were euthanized once tumours began to impede movement, show signs of ulceration or if any measurement of the tumour exceeded 30 mm (length, width or depth).

## Author contributions

**Denis Tvorogov:** Conceptualization; Formal analysis; Supervision; Funding acquisition; Validation; Investigation; Visualization; Methodology; Writing—original draft; Writing—review and editing. **Chloe A L Thompson‐Peach:** Formal analysis; Supervision; Validation; Investigation; Visualization; Methodology; Writing—original draft; Writing—review and editing. **Johannes Foßelteder:** Formal analysis; Investigation; Methodology; Writing—review and editing. **Mara Dottore:** Validation; Investigation; Methodology. **Frank Stomski:** Investigation; Methodology. **Suraiya A Onnesha:** Investigation. **Kelly Lim:** Investigation; Methodology. **Paul A Moretti:** Resources; Investigation; Methodology. **Stuart M Pitson:** Resources; Investigation; Methodology. **David M Ross:** Resources; Writing—original draft; Writing—review and editing. **Andreas Reinisch:** Resources; Investigation; Methodology; Writing—review and editing. **Daniel Thomas:** Conceptualization; Data curation; Supervision; Funding acquisition; Writing—original draft; Writing—review and editing. **Angel F Lopez:** Conceptualization; Funding acquisition; Writing—original draft; Writing—review and editing.

In addition to the CRediT author contributions listed above, the contributions in detail are:

DTv, DTh and AFL conceptualized the data. DTv, CALT‐P, MD, JF, FS, SAO, KL, AR and DTh performed experiments and analysed data. DTv, FS, PABM and SMP generated reagents. AR and DMR provided patient samples. DTh, DTv, CALT‐P, DMR and AFL wrote the manuscript.

## Supporting information



Expanded View Figures PDF

Table EV1

Source Data for Expanded View

Source Data for Figure 2

Source Data for Figure 4

## Data Availability

No large primary datasets have been generated and deposited.
