# Supplementary material for: Targeting human CALR‐mutated MPN progenitors with a neoepitope‐directed monoclonal antibody
Source: EMBO Rep. 2022 Feb 14;23(4):e52904. doi: 10.15252/embr.202152904 (PMC8982588; doi:10.15252/embr.202152904)
Supplement: Supplementary file 1 — Expanded View Figures PDF [file EMBR-23-e52904-s001.pdf]

Expanded View Figures

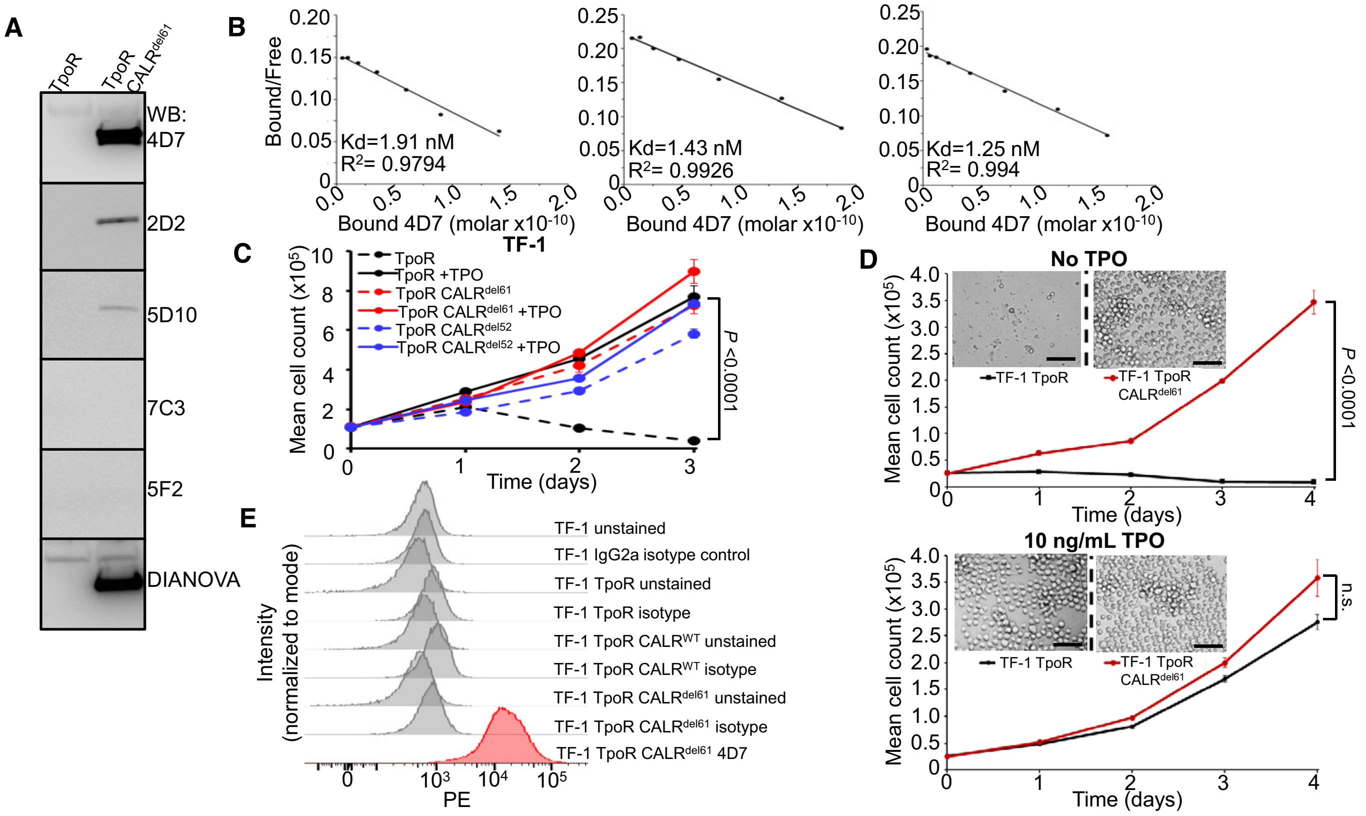

Figure EV1. Characterization of 4D7 antibody as a potential therapeutic tool.

A Western blot screening of various mutant CALR antibody clones produced from hybridomas in TF-1 TpoR and TF-1 TpoR CALR^{del61} cells. Varying levels of intensity can be observed compared to commercial Dianova monoclonal mutCALR antibody. 4D7 is able to detect mutant CALR protein in CALR^{del61} cells but not in CALR wild-type TF-1 cells.

B Biological replicate Scatchard analyses of 4D7 using ^{125}I-labelled and unlabelled 4D7 bound to full-length peptide (n = 3 technical replicates).

C TF-1 cells expressing TpoR and CALR^{del61} or CALR^{del52} demonstrate factor independence in absence of TPO. Cells were cultured in the presence or absence of 10 ng/ml TPO (n = 3 biological replicates).

D Paracrine CALR-mutant protein is not sufficient to maintain TPO-sensitive cells in culture. TF-1 TpoR and TF-1 TpoR CALR^{del61} cells were seeded at the same density and cultured in the presence or absence of TPO. Cells were separated by semi-permeable membrane in a horizontal co-culture system. Cell populations on either side of the membrane were counted every 24 h over 4 days in triplicate and representative images were taken on day 4. Exogenous CALR secreted by TF-1 TpoR CALR^{del61} was unable to assist growth of factor-dependent TF-1 TpoR cells. Scale bar indicates 100 μm (n = 3 biological replicates).

E Histogram overlays showing fluorescence intensity of unstained and PE-conjugated IgG2a isotype control in TF-1, TF-1 TpoR, TF-1 TpoR CALR^{WT} and TF-1 TpoR CALR^{del61} compared to 4D7 conjugated to PE.

Source data are available online for this figure.

© 2022 The Authors

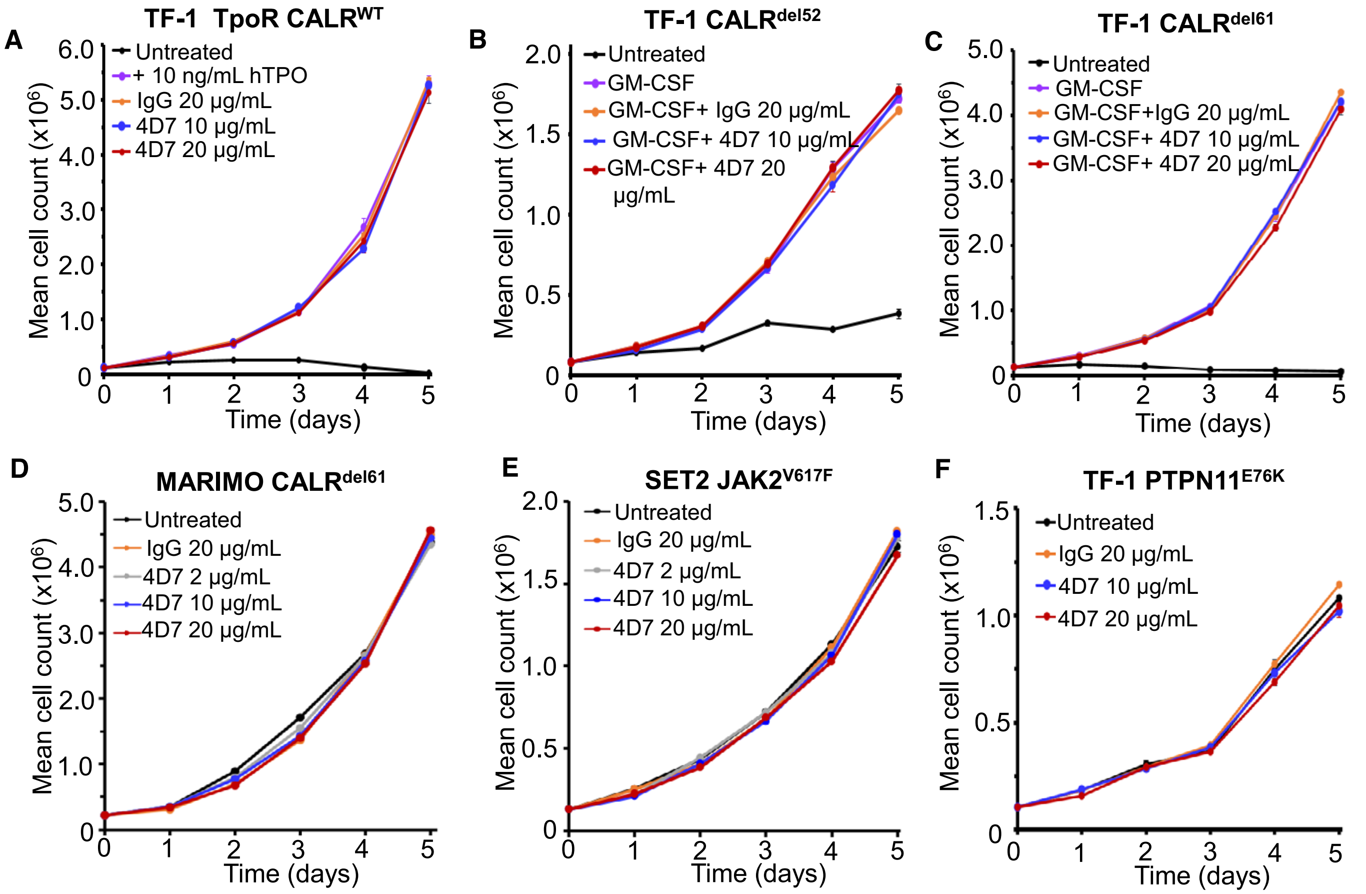

Figure EV2. Biological specificity of 4D7.

- A Cytokine-dependent TF-1 TpoR cells with an overexpression of WT CALR cultured in the absence of TPO, 10 ng/ml hTPO and 10 or 20 µg/ml 4D7 or 20 µg/ml control IgG antibody for 5 days and the number of trypan blue-negative cells were counted every 24 h (n = 3 biological replicates with three technical replicates).
- B Cytokine-dependent TF-1 CALR^{del52} cells lacking TpoR were cultured in the presence of 2 ng/ml GM-CSF and 10 or 20 µg/ml 4D7 or 20 µg/ml control IgG antibody (n = 3 biological replicates with three technical replicates).
- C Cytokine-dependent TF-1 CALR^{del61} cells lacking TpoR were cultured in the presence of 2 ng/ml GM-CSF and 10 or 20 µg/ml 4D7 or 20 µg/ml control IgG antibody (n = 3 biological replicates with three technical replicates).
- D MARIMO cells from which the CALR^{del61} mutation was originally amplified were cultured in the presence of 2, 10 or 20 µg/ml 4D7 or 20 µg/ml control IgG antibody (n = 3 biological replicates with three technical replicates).
- E Cytokine-independent SET2 cells which harbour the pathogenic JAK2^{V617F} mutation were cultured in the presence of 2, 10 or 20 µg/ml 4D7 or 20 µg/ml control IgG antibody (n = 3 biological replicates with three technical replicates).
- F Cytokine-independent TF-1 PTPN11^{E76K} cells were cultured in the presence of 10 or 20 µg/ml 4D7 or 20 µg/ml control IgG antibody (n = 3 biological replicates with three technical replicates).

Data information: For all panels, bars represent standard error of the mean.

 © 2022 The Authors

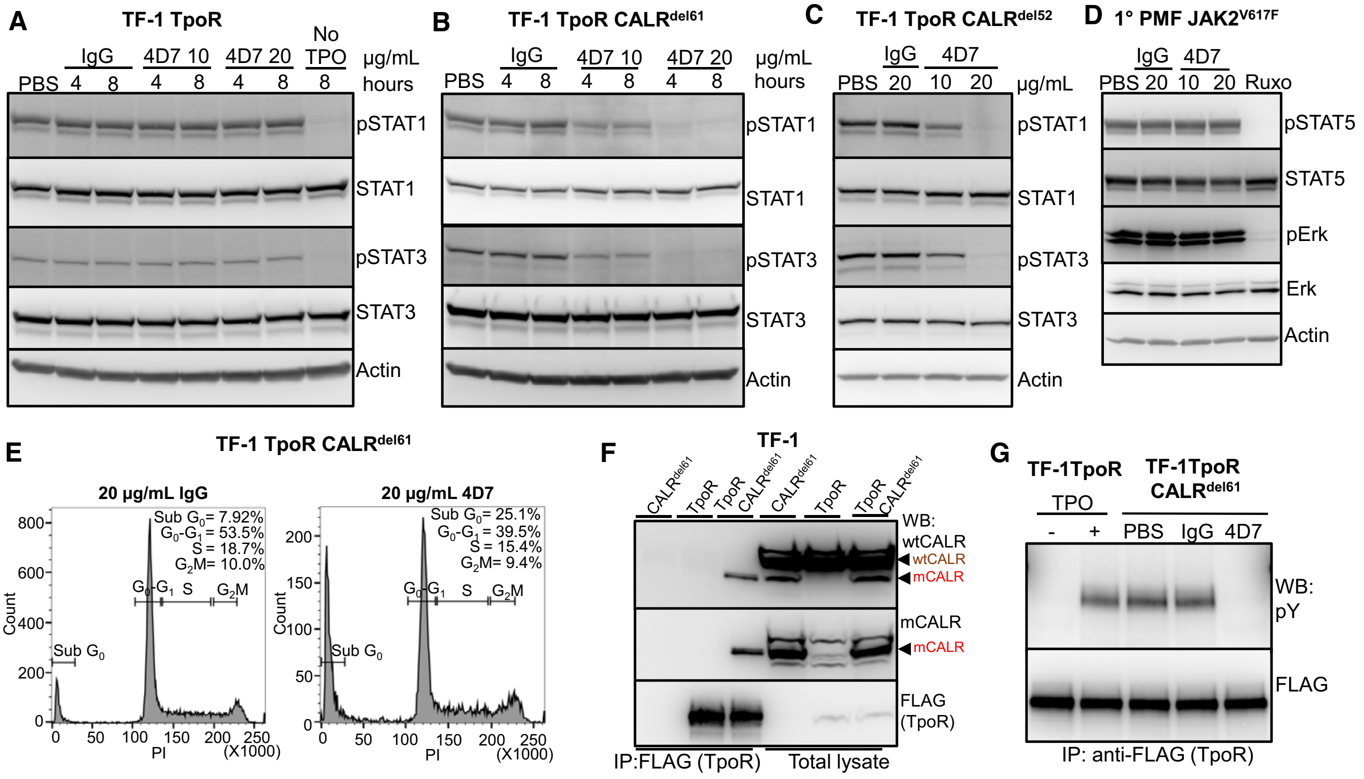

Figure EV3. Monoclonal antibody blocks STAT1, 3 signalling and TpoR phosphorylation.

- A Cell extracts blotted for phospho-STAT1, total STAT1, phospho-STAT3, total STAT3 and actin from TF-1 TpoR cells after incubation with 10 or 20 µg/ml 4D7 or IgG for 4 or 8 h as indicated.
- B Similar experiment using TPO-independent TF-1 TpoR CALR^{del61} cells at 4 and 8 h.
- C Similar experiment shown using TPO-independent TF-1 TpoR CALR^{del52} cells, 8 h.
- D Similar experiment using PBMNCs from JAK2^{V617F} PMF primary cells at 8 h. Additionally, cells were treated with 280 nM of ruxolitinib as a positive control.
- E Flow cytometry analysis for cell cycle distribution of TF-1 TpoR CALR^{del61} cells exposed to 20 µg/ml IgG or 4D7 for 48 h. Cells were harvested and fixed and stained with propidium iodide and their DNA contents were analysed. Results from one representative experiment shown. Percentages of cells in Sub G_0, G_1, S and G_2/M cycle indicated.
- F Western blot showing specific co-immunoprecipitation of mutant CALR^{del61} cells with TpoR anti-FLAG antibody under reducing conditions detected by WT and mutant-specific CALR antibodies. Red arrowheads, detected mutant CALR protein; brown arrowheads, detected wild-type CALR protein.
- G Western blot showing decreased TpoR phosphorylation in a TpoR immunoprecipitated after 8 h of 20 µg/ml 4D7 treatment compared to PBS or IgG control in TF-1 TpoR CALR^{del61}.

Source data are available online for this figure.

© 2022 The Authors

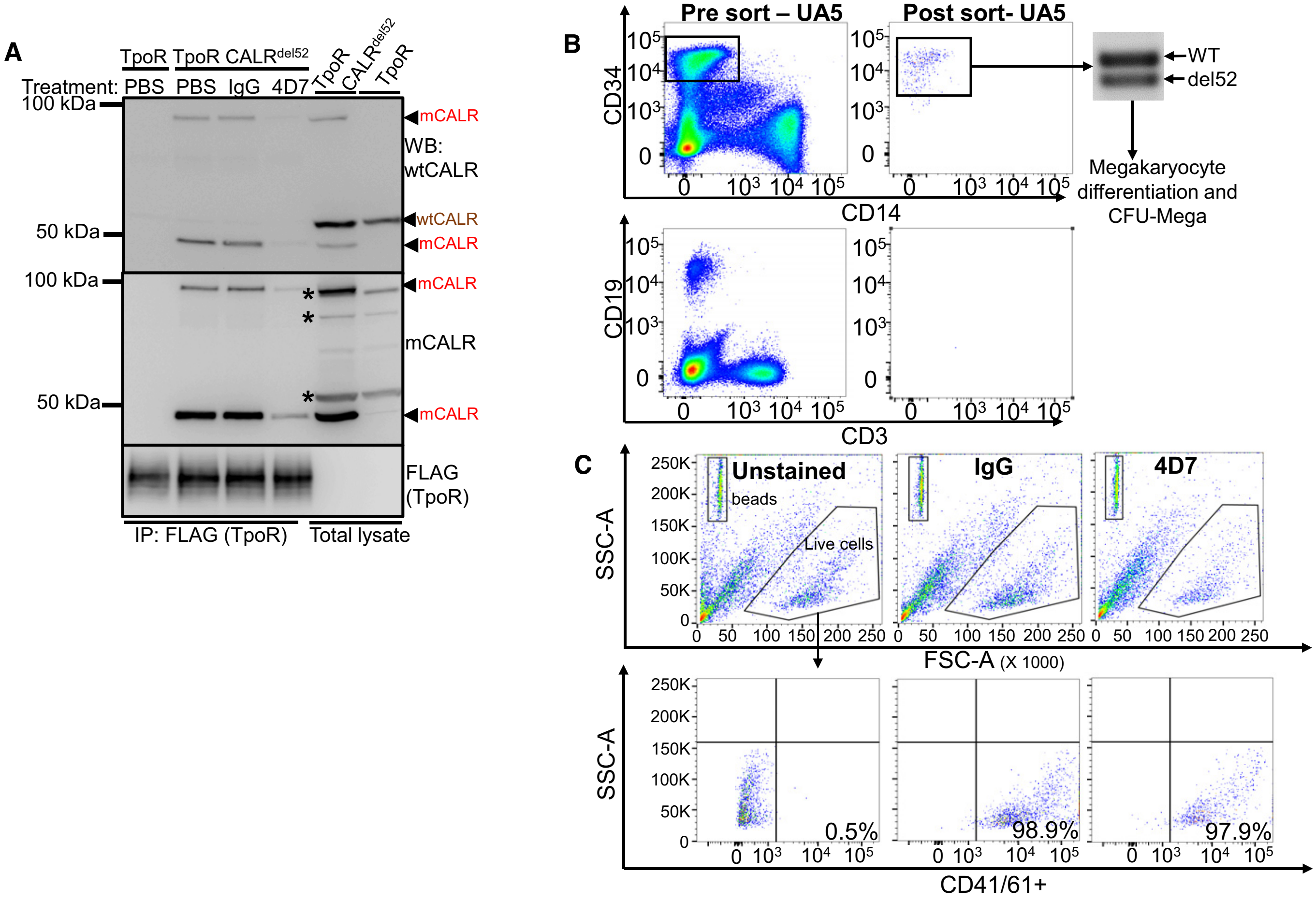

Figure EV4. 4D7 blocks CALR interaction with TpoR in CALR^{del52} cells.

A Western blot of TpoR immunoprecipitation under non-reducing conditions showing associated CALR 50 kDa monomers and 100 kDa dimers (red arrowheads) present only in TF-1 TpoR CALR^{del52} disrupted by 8-h treatment with 20 μg/ml 4D7 but not PBS or 20 μg/ml IgG. CALR monomers and dimers are detectable by polyclonal anti-wild-type CALR or anti-mutant CALR monoclonal antibodies. Red arrowheads, detected mutant CALR protein; brown arrowheads, detected wild-type CALR protein; asterisk, non-specific bands.

B Peripheral blood mononuclear cells from PMF samples were thawed and stained for CD34, CD14, CD19 and CD3 prior to FACS sorting. Each population was collected and purity was verified prior to proceeding with any further analysis. PCR amplification of CALR exon 9 was carried out to confirm mutational status of CD34^{+} cells which were utilized in megakaryocyte differentiation assays and colony forming assays in the presence of 4D7 or IgG.

C Representative flow cytometry plots for determination of CD41^{+}/61^{+} populations from liquid culture assay from one PMF patient. Beads shown in the upper left panel with high SSC-A. Live cell population shown in hexagon gate (top panel). CD41/61^{+} population gates shown in lower panel with % CD41/61^{+} cells indicated for unstained, IgG- and 4D7-treated cells over 12 days. The number of CD41/61^{+} cells-to-bead ratio used to enumerate effect of 4D7 on megakaryopoiesis.

Source data are available online for this figure.

 © 2022 The Authors

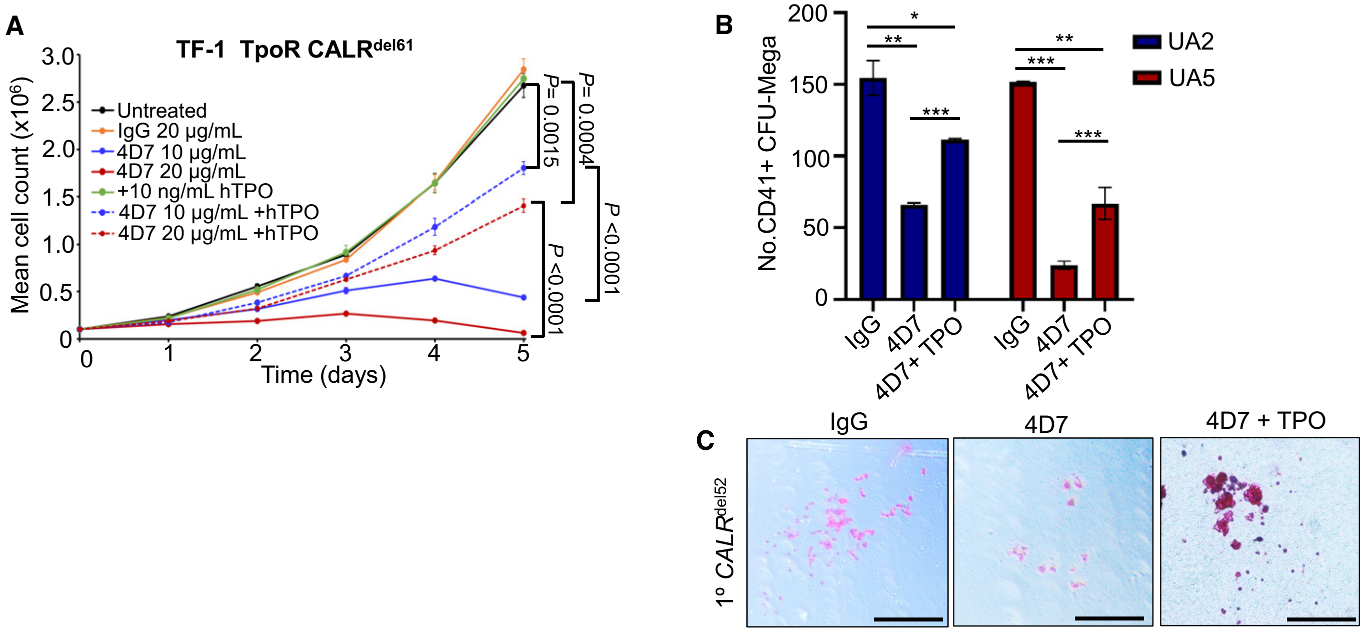

Figure EV5. Effect of 4D7 antibody on TPO receptor biology.

A Proliferation curves of factor-independent TF-1 TpoR CALR^{del61} cells cultured with 10 or 20 µg/ml 4D7 or 20 µg/ml of control IgG antibody in presence or absence of 10 ng/ml TPO (n = 3 biological replicates with three technical replicates).

B Number of CD41^{+} megakaryocyte colonies CALRdel52 patient after 4D7 treatment in the presence of TPO. Samples were seeded in a collagen-based matrix in presence of 20 µg/ml 4D7 or IgG control with 50 ng/ml TPO (n = 2 patient samples with two technical replicates).

C Representative micrographs showing CD41^{+} colonies in pink and CD41- colonies in blue after treatment with IgG or 4D7 in absence or presence of 50 ng/ml TPO. Scale bar indicates 100 µm.

Data information. Error bars represent standard error of the mean in (A) and standard deviation (B). Unpaired Student's t-test used to determine statistical significance in (B). *P = 0.05–0.01, **P = 0.01–0.001, ***P = 0.001–0.0001.
