# Supplementary material for: Targeting human CALR‐mutated MPN progenitors with a neoepitope‐directed monoclonal antibody
Source: EMBO Rep. 2022 Feb 14;23(4):e52904. doi: 10.15252/embr.202152904 (PMC8982588; doi:10.15252/embr.202152904)
Supplement: Supplementary file 2 — Table EV1 [file EMBR-23-e52904-s006.pdf]

Sample ID	Diagnosis (Dx)	Date of Dx	Age at Dx	Sex	Karyotype	Mutational Status	Spleen	Hb (g/L)	PLT ($x10^9/L$)	WCC ($x10^9/L$)	PB Blast (%)	Ruxo Therapy	Ruxo Start Date
H02668 (UA1)	PMF	16/11/2007	75.5	M	Failed	CALR:c.1099_1150del (p.Leu367Thrfs*46)	Not palpable	TF	267	5.4	0	N	N/A
H05150 (UA2)	PMF	14/12/2018	66.7	F	46XX	CALR:c.1099_1150del (p.Leu367Thrfs*46)	15cm via sonography	TF	1111	13.5	0	N	N/A
H01608 (UA3)	PMF	19/08/2014	30.3	M	46XY	CALR:c.1103_1136del 34 (p.Lys368Argfs*51)	Not palpable	145	883	7.6	0	N	N/A
H01244 (UA4)	PMF	11/06/2002	58.6	F	46XXdel(20)(q11 .2q13.3)	CALR:c.1099_1150del (p.Leu367Thrfs*46)	Palpable (was 6 cm 1 year earlier)	100	109	2.1	1	Y	10/01/2010
H04082 (UA5)	PMF	7/12/2010	54.5	F	46XX	CALR:c.1099_1150del (p.Leu367Thrfs*46)	13cm, palpable	111	403	23.6	4	N	N/A
H01346 (UA6)	PMF	28/04/2014	64.9	M	46XY	CALR:c.1099_1150del (p.Leu367Thrfs*46)	Not palpable	135	1019	12.2	0	N	N/A
9793	pET-MF	05/03/2001 (ET), 20/04/2016 (pET-MF)	25.8 (ET), 40.9 (pET-MF)	F	46XXt(9;22)(q22; q13)	CALR:c.1099_1150del (p.Leu367Thrfs*46)	No record	109	199	5.73	2	Y	06/2013 (discontinued after 4 months)
9799	PMF	19/11/2018	55.3	F	Failed	CALR:c.1099_1150del (p.Leu367Thrfs*46)	22cm via sonography	91	251	16.2	4	Y	19/02/2019
9800	pET-MF	08/1991 (ET), 17/04/2018 (pET-MF)	33.5 (ET), 60.2 (pET-MF)	F	46XX	CALR:c.1154_1155insT TGTC (p.Lys385Asnfs*47)	16cm via sonography	100	537	9.1	1	Y	21/02/2020

Dx, diagnosis; ET, essential thrombocythemia; Hb, haemoglobin; PB, peripheral blood; pET-MF, post-essential thrombocythemia myelofibrosis; PLT, platelet; PMF, primary myelofibrosis; TF, Transfusion dependent; WCC, white blood cell count.
