# Supplementary material for: Targeting human CALR‐mutated MPN progenitors with a neoepitope‐directed monoclonal antibody
Source: EMBO Rep. 2022 Feb 14;23(4):e52904. doi: 10.15252/embr.202152904 (PMC8982588; doi:10.15252/embr.202152904)
Supplement: Supplementary file 3 — Source Data for Expanded View [file EMBR-23-e52904-s004.zip › EV_Figure_Source_data/Fig_EV1_PDF_RAW_DATA.pdf]

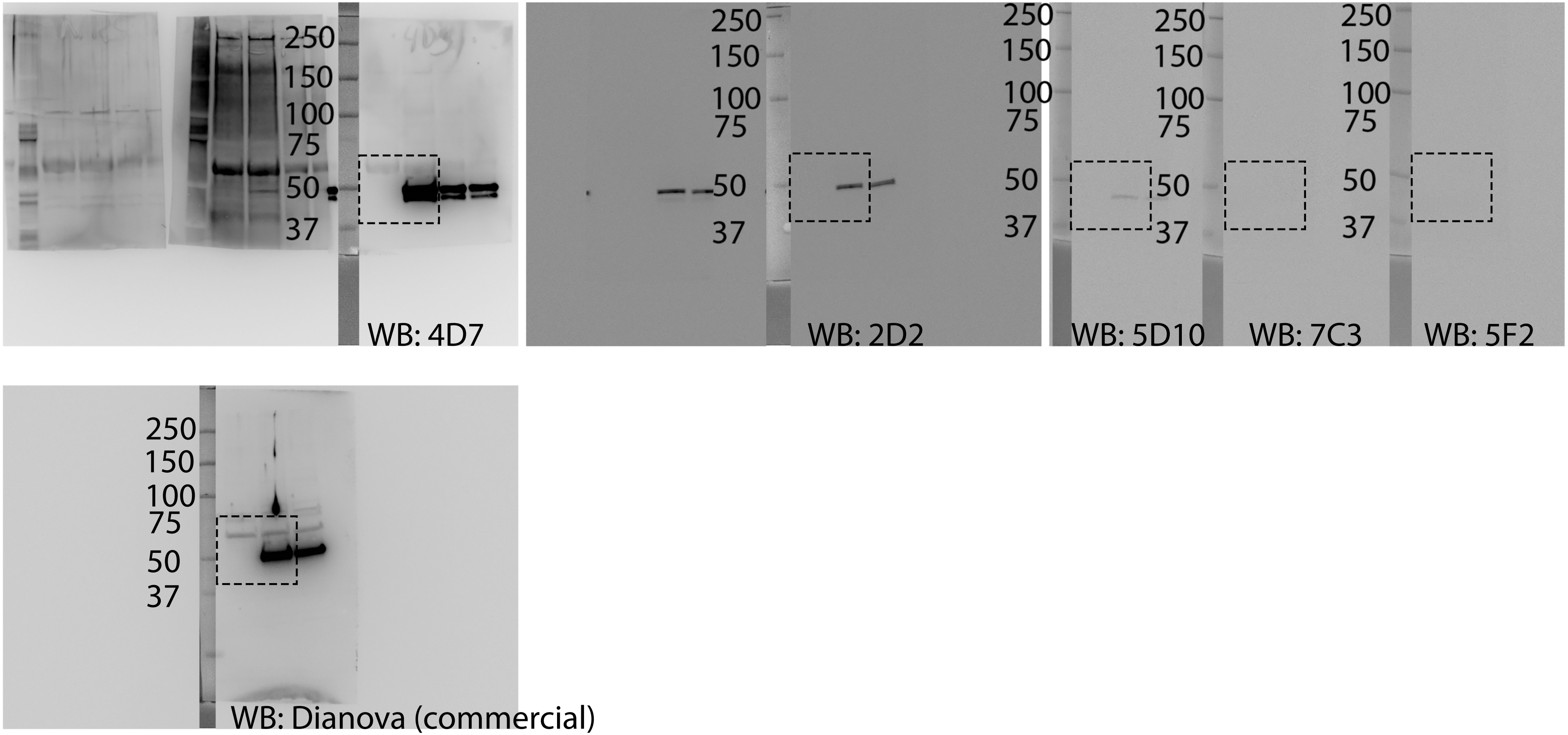

Above blots belong to Figure EV1 Panel A and placed in the same order as in panel
75kDa marker appears dim in all blots. Some blots represent the testing of several antibody clones which were not included in Figure.
