# Supplementary material for: Targeting human CALR‐mutated MPN progenitors with a neoepitope‐directed monoclonal antibody
Source: EMBO Rep. 2022 Feb 14;23(4):e52904. doi: 10.15252/embr.202152904 (PMC8982588; doi:10.15252/embr.202152904)
Supplement: Supplementary file 3 — Source Data for Expanded View [file EMBR-23-e52904-s004.zip › EV_Figure_Source_data/Fig_EV3_PDF_RAW_DATA.pdf]

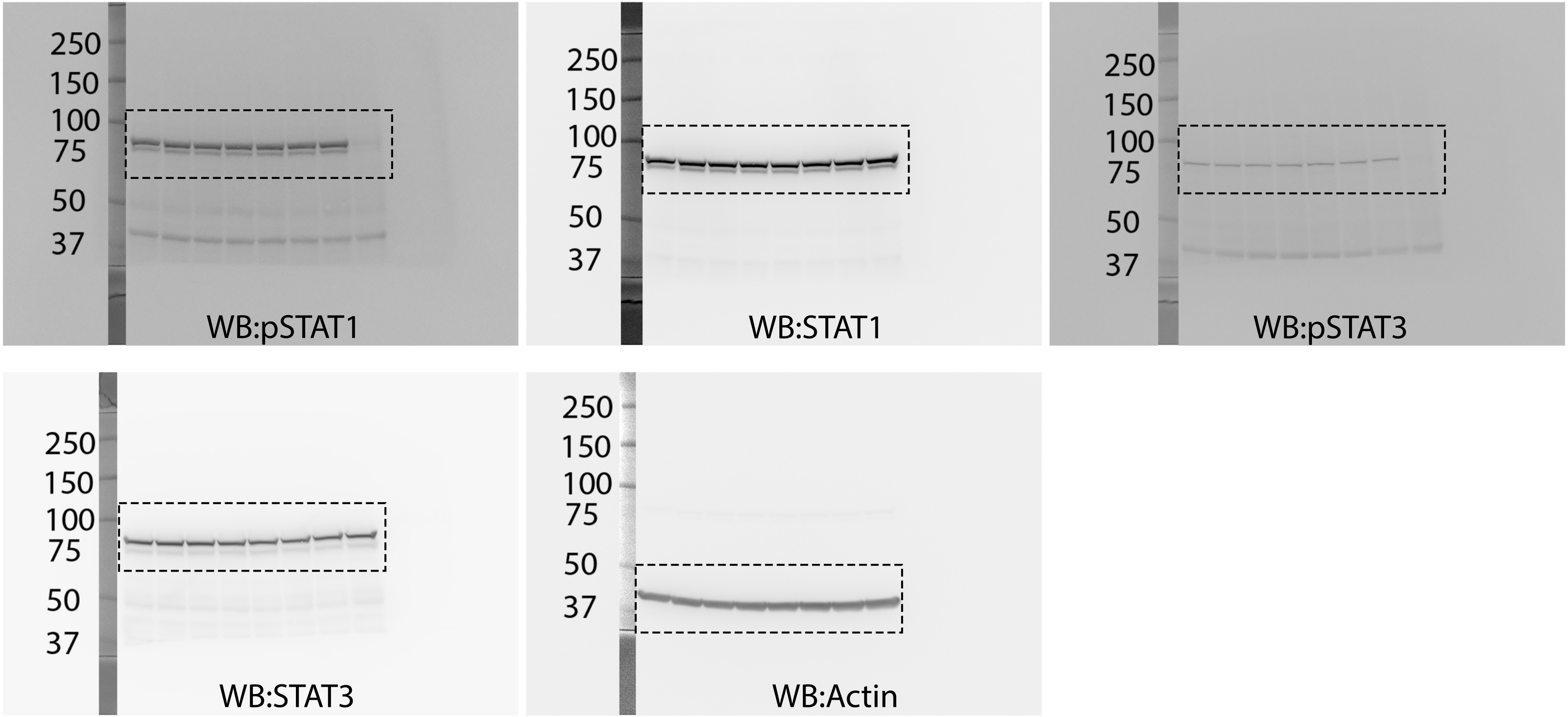

Above blots belong to Figure EV3 Panel A and placed in the same order as in panel
75kDa marker appears dim in all blots.

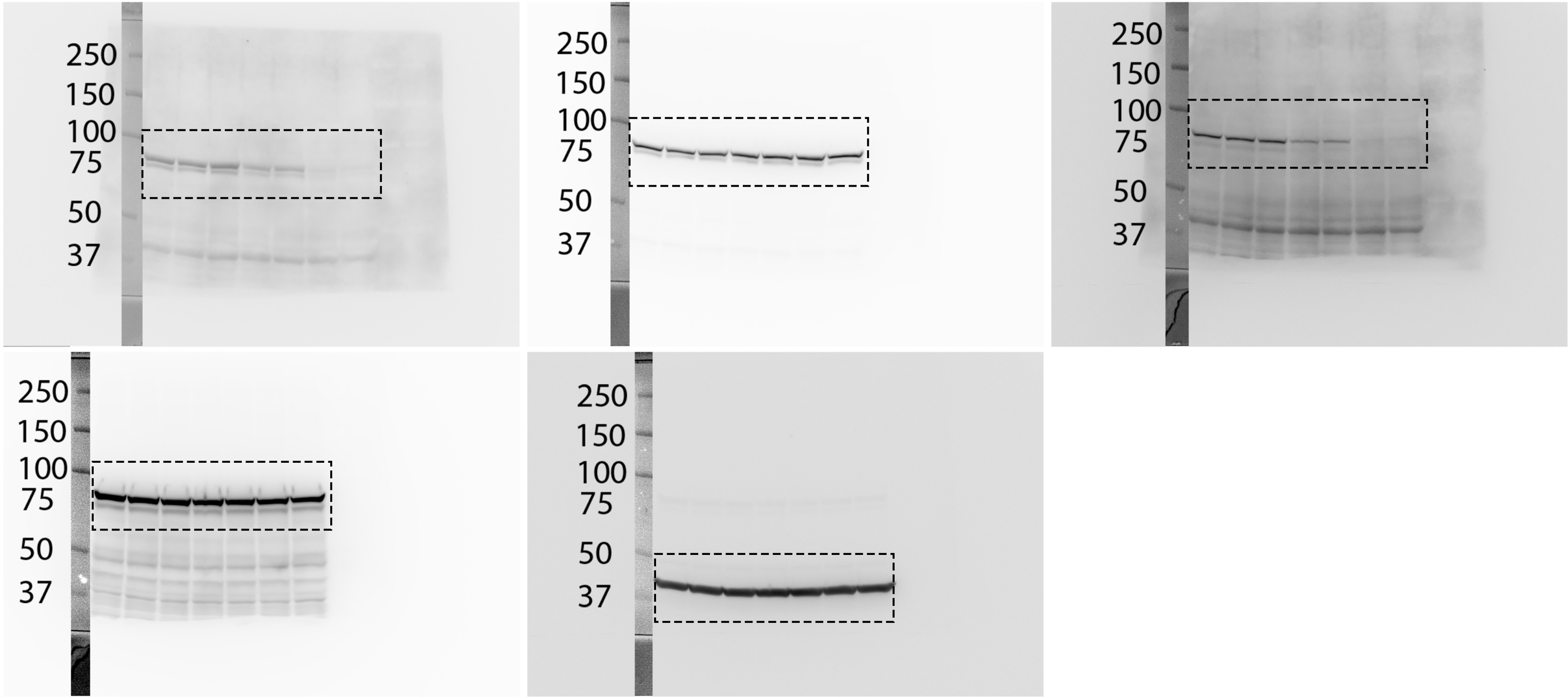

Above blots belong to Figure EV3 Panel B and placed in the same order as in panel
75kDa marker appears dim in all blots.

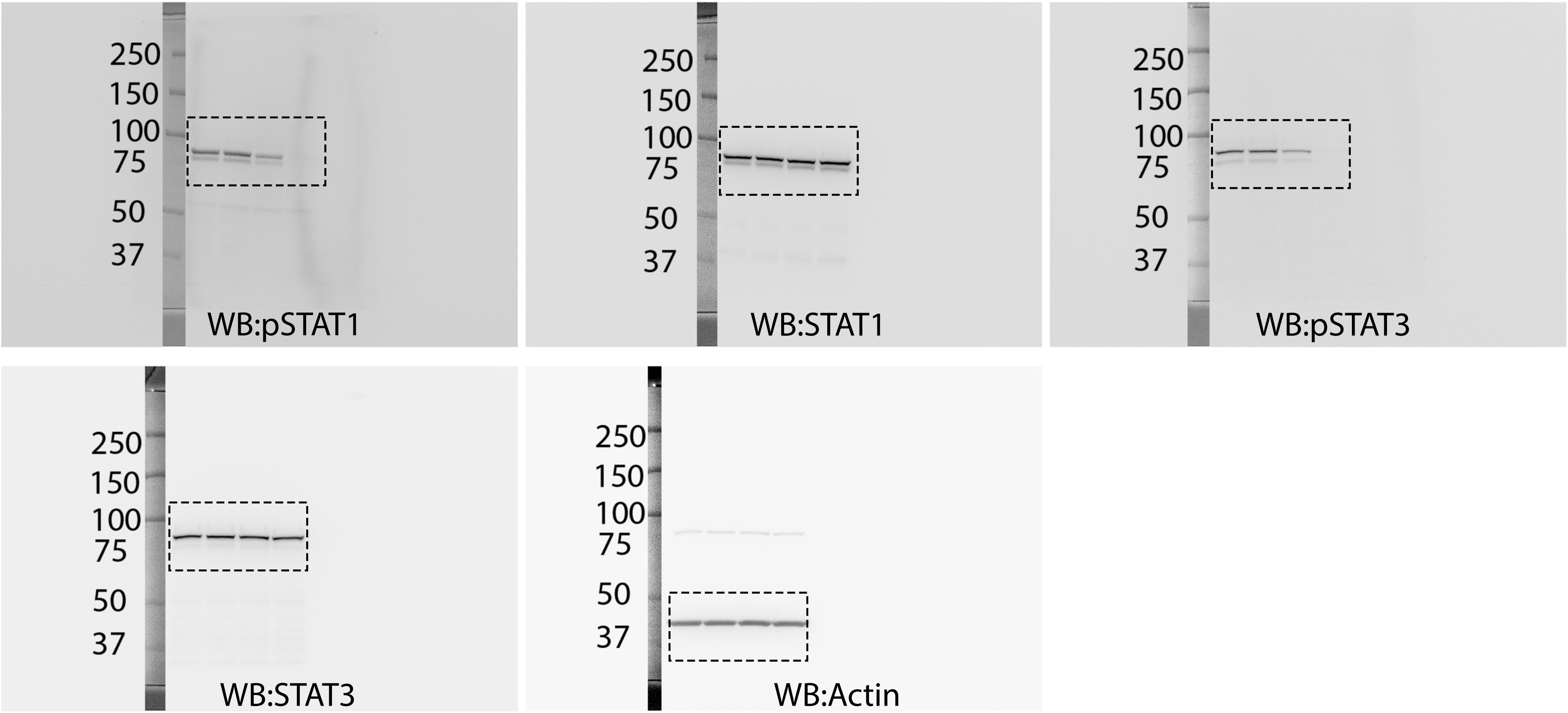

Above blots belong to Figure EV3 Panel C and placed in the same order as in panel
75kDa marker appears dim in all blots.

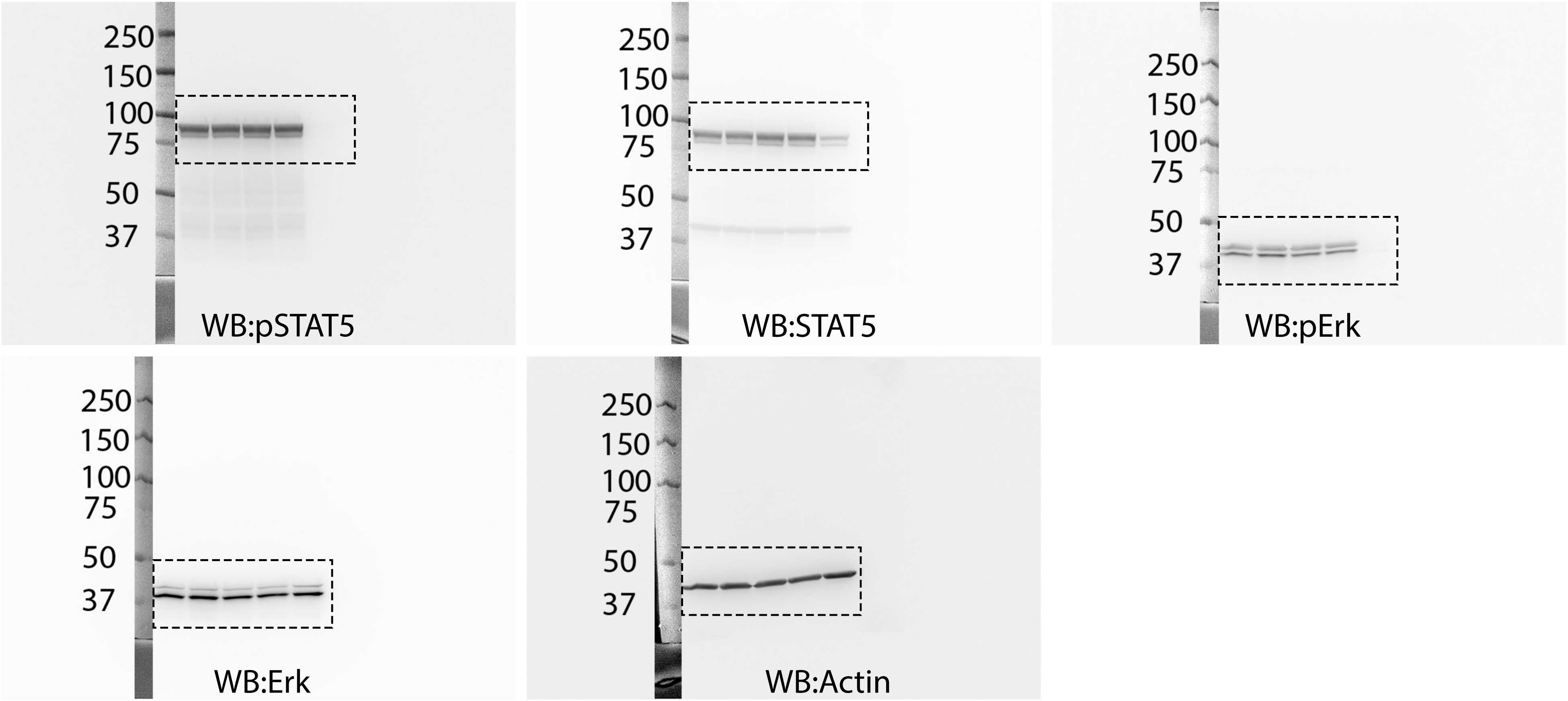

Above blots belong to Figure EV3 Panel D and placed in the same order as in panel
75kDa marker appears dim in all blots.

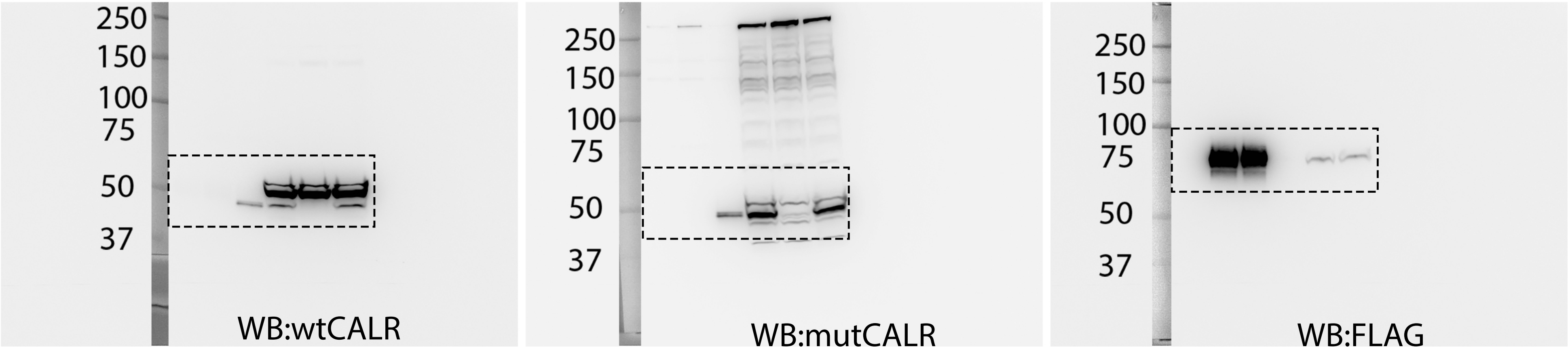

Above blots belong to Figure EV3 Panel F and placed in the same order as in panel
75kDa marker appears dim in all blots.

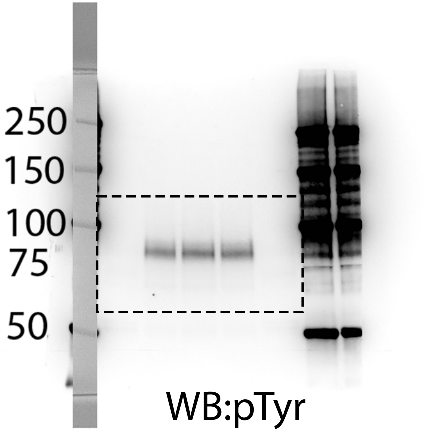

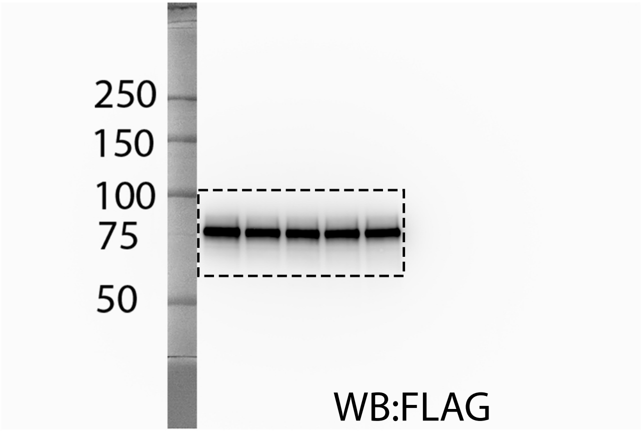

Above blots belong to Figure EV 3 Panel G and placed in the same order as in panel
On pTyr western blot protein markers can be seen in visible light and in chemiluminescence as anti-pTyr antibody nonspecifically bind to BioRad Kaleidoscope markers.
75kDa marker appears dim in all blots.
