# Supplementary material for: Targeting human CALR‐mutated MPN progenitors with a neoepitope‐directed monoclonal antibody
Source: EMBO Rep. 2022 Feb 14;23(4):e52904. doi: 10.15252/embr.202152904 (PMC8982588; doi:10.15252/embr.202152904)
Supplement: Supplementary file 3 — Source Data for Expanded View [file EMBR-23-e52904-s004.zip › EV_Figure_Source_data/FIG_EV_4A_PDF_RAW_DATA.pdf]

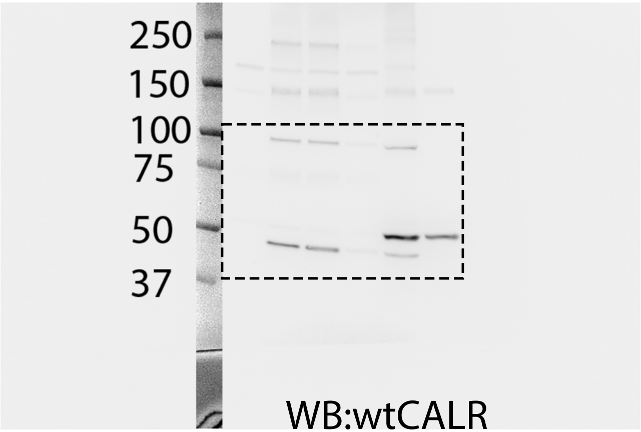

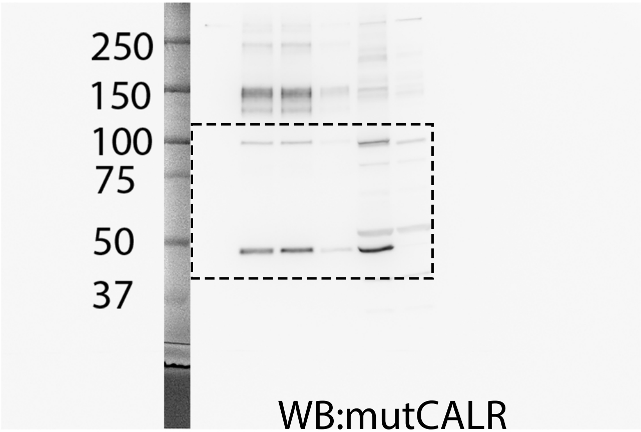

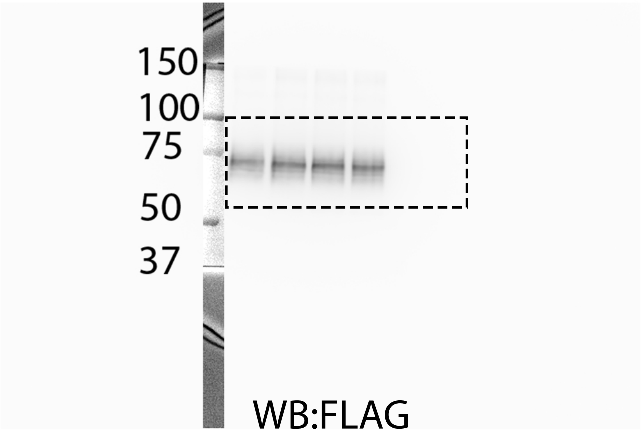

Above blots belong to Figure EV4 Panel A and placed in the same order as in panel
75kDa marker appears dim in all blots.
