# Supplementary material for: Targeting human CALR‐mutated MPN progenitors with a neoepitope‐directed monoclonal antibody
Source: EMBO Rep. 2022 Feb 14;23(4):e52904. doi: 10.15252/embr.202152904 (PMC8982588; doi:10.15252/embr.202152904)
Supplement: Supplementary file 4 — Source Data for Figure 2 [file EMBR-23-e52904-s002.pdf]

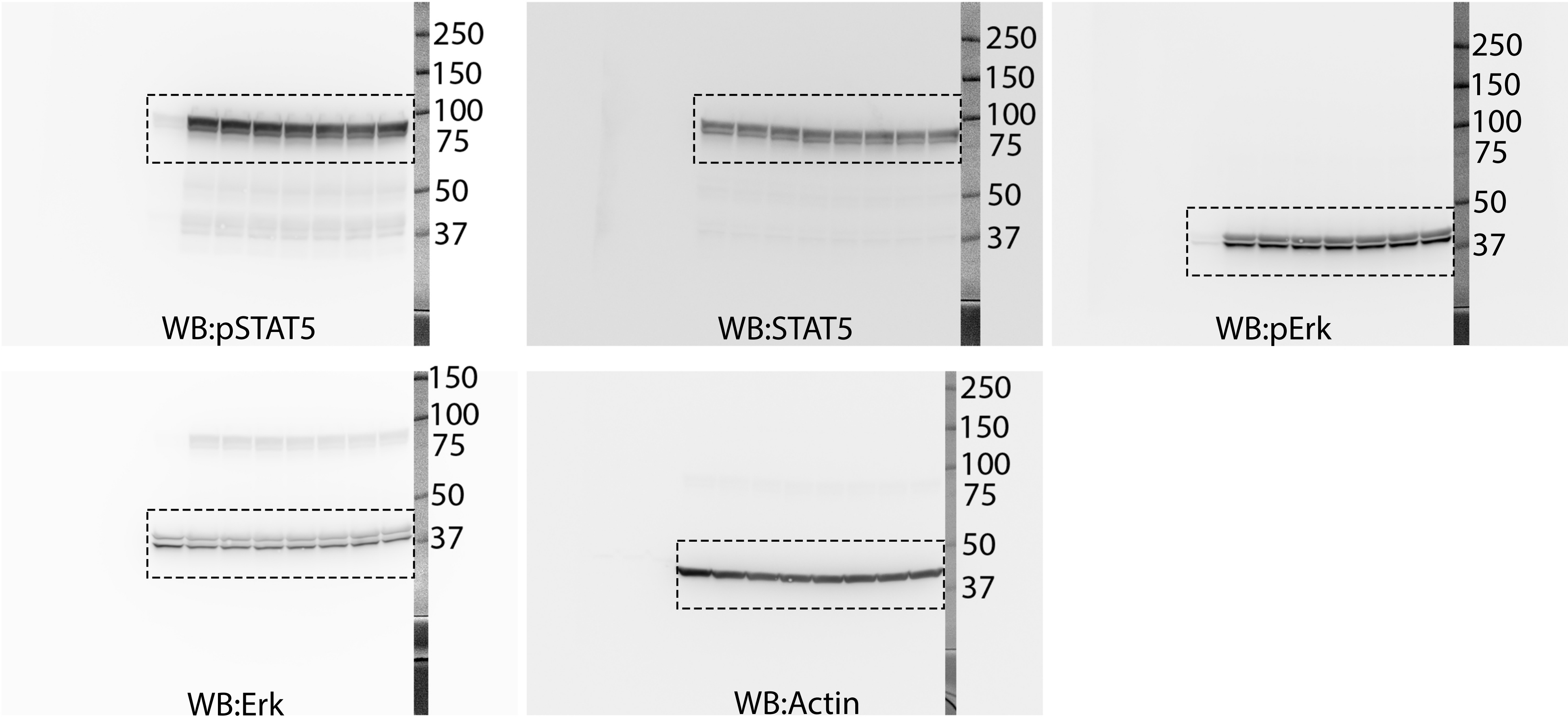

Above blots belong to Figure 2 Panel A and placed in the same order as in panel
Additionally, for Figure 2A all blots were flipped horizontally to allow direct
comparison with other panels. 75kDa marker appears dim in all blots.

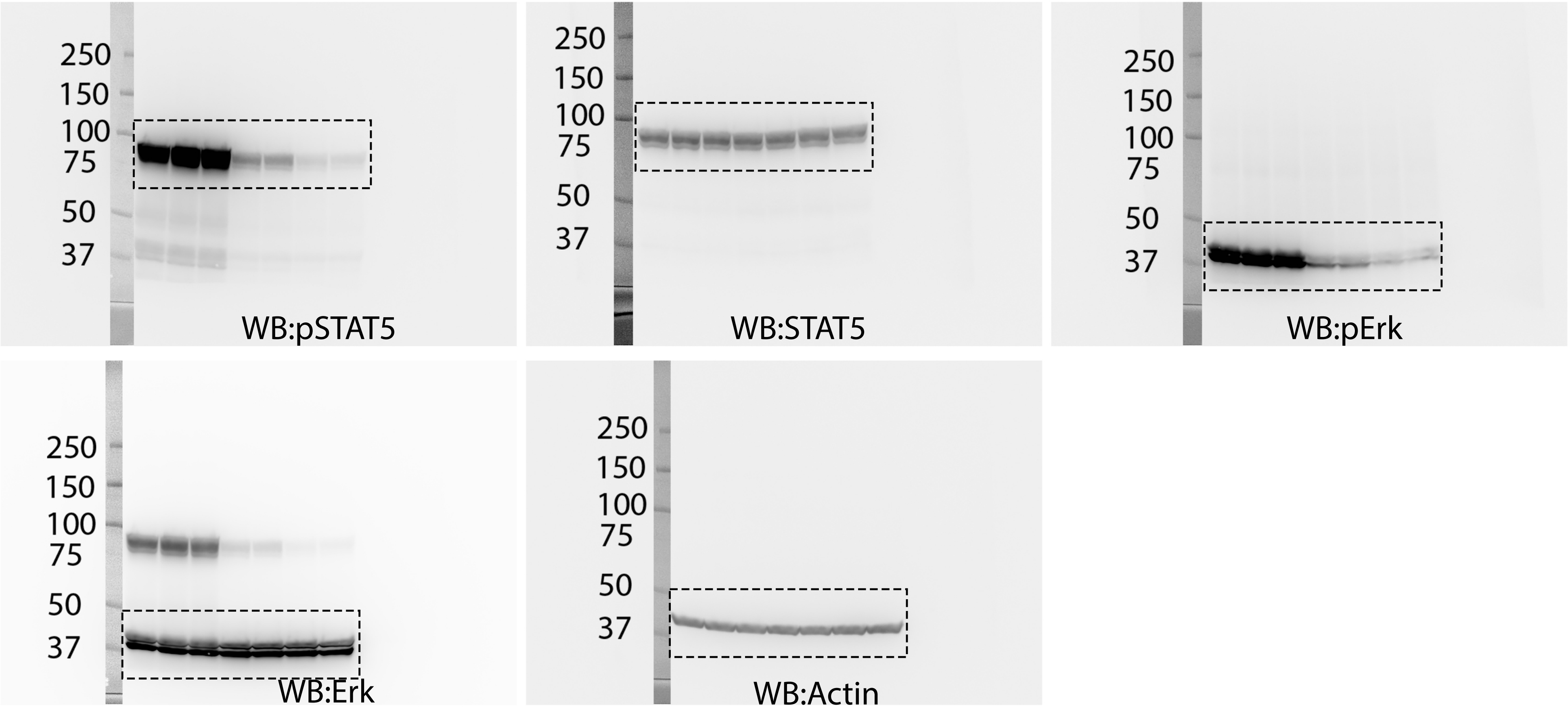

Above blots belong to Figure 2 Panel B and placed in the same order as in panel
75kDa marker appears dim in all blots.

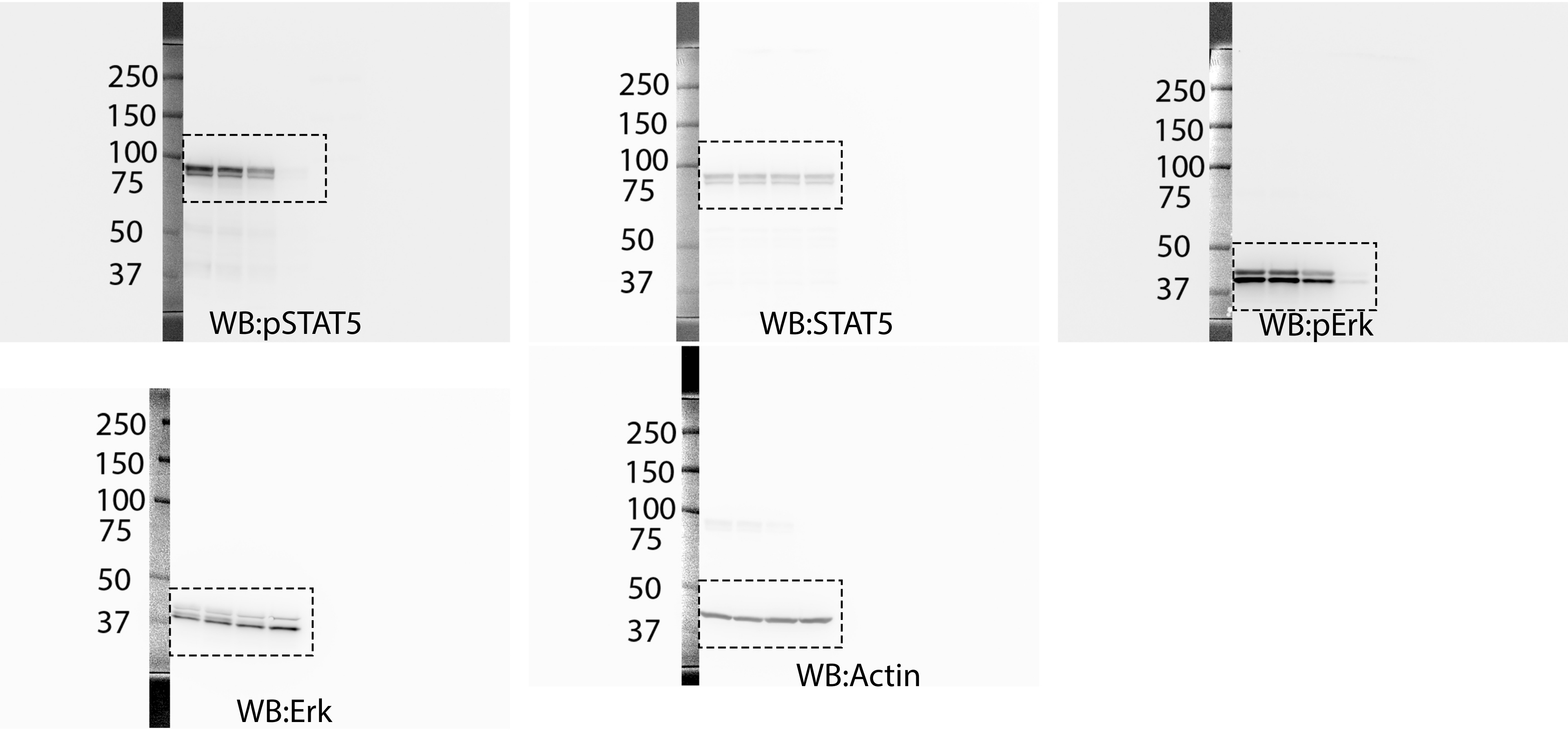

Above blots belong to Figure 2 Panel C and placed in the same order as in panel
75kDa marker appears dim in all blots.

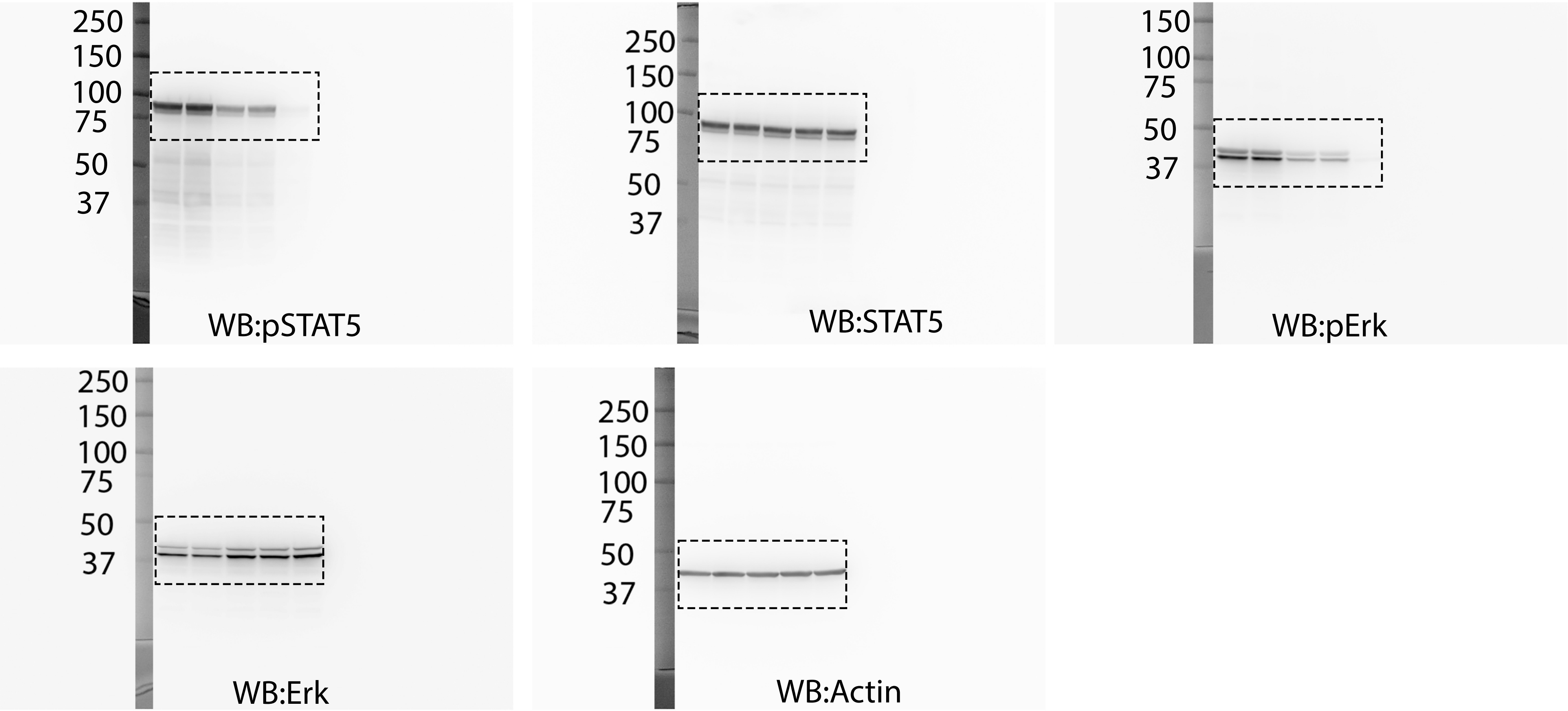

Above blots belong to Figure 2 Panel D and placed in the same order as in panel
75kDa marker appears dim in all blots.

Total caspase 3
TF1-TpoR, TF1 TpoR CALR^{del61}

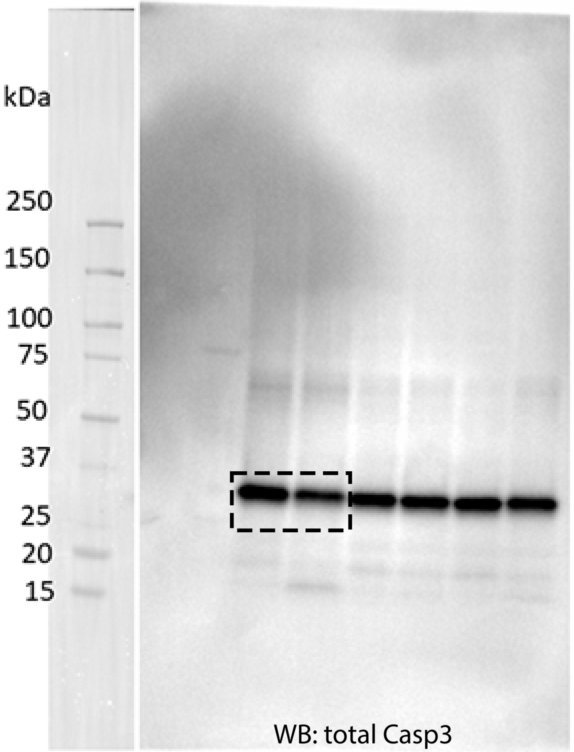

Cleaved caspase 3
TF1-TpoR, TF1 TpoR CALR^{del61}

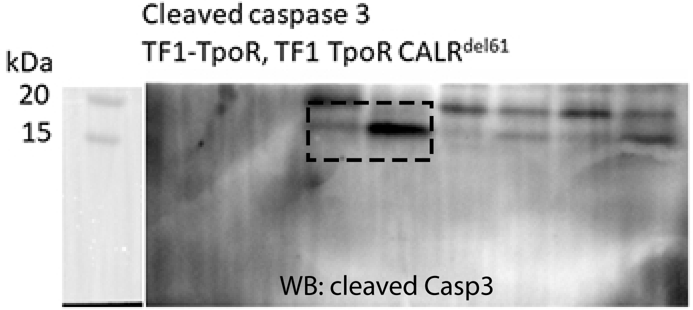

Actin
TF1-TpoR, TF1 TpoR CALR^{del61}

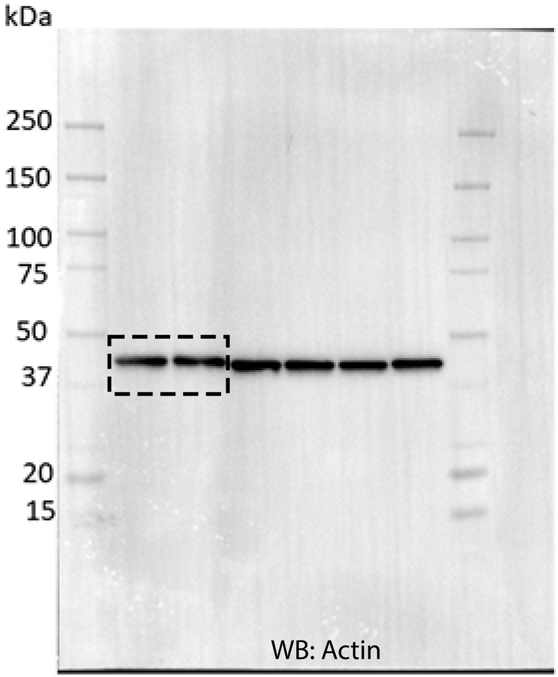

Above blots belong to Figure 2 Panel F left panel

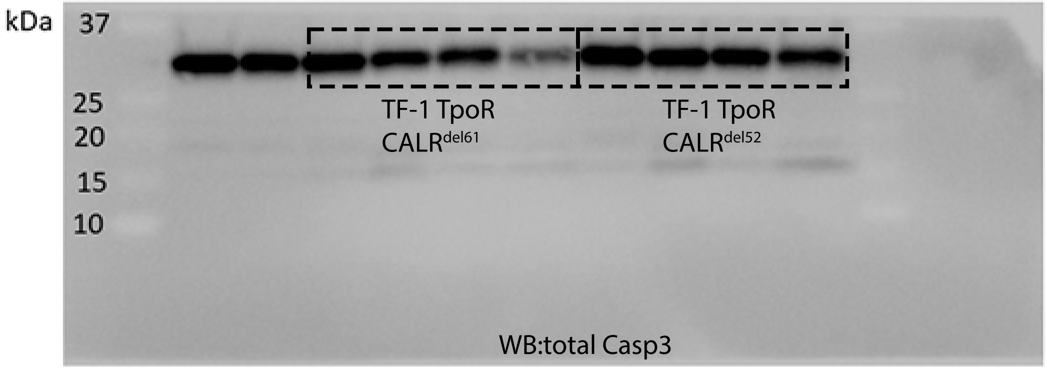

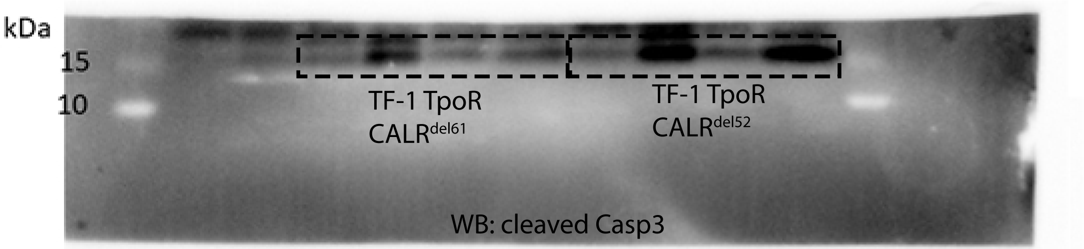

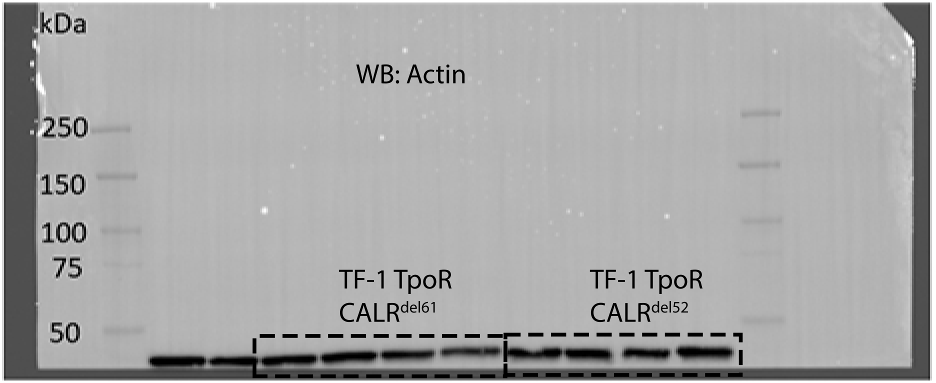

Above blots belong to Figure 2 Panel F middle and right panel

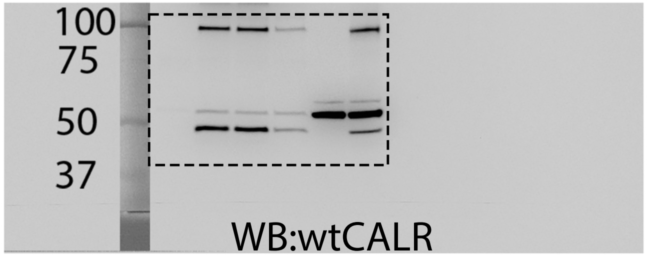

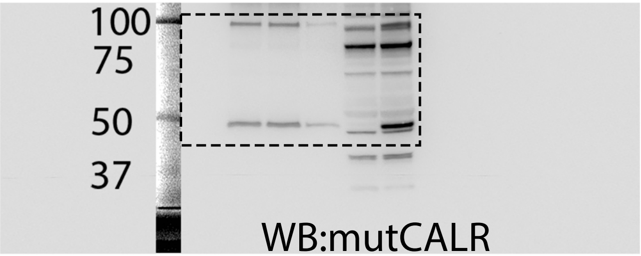

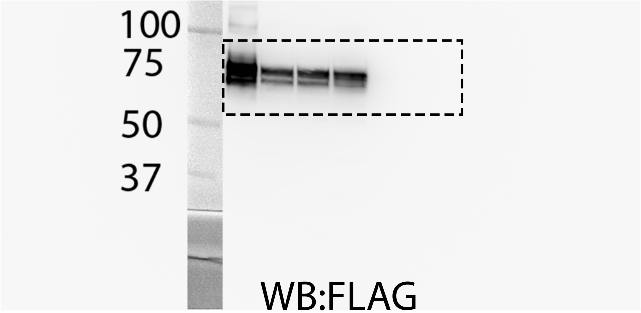

Above blots belong to Figure 2 Panel G and placed in the same order as in panel
75kDa marker appears dim in all blots.
