# Supplementary figures and images for: Targeting human CALR‐mutated MPN progenitors with a neoepitope‐directed monoclonal antibody
Source: EMBO Rep. 2022 Feb 14;23(4):e52904. doi: 10.15252/embr.202152904 (PMC8982588; doi:10.15252/embr.202152904)

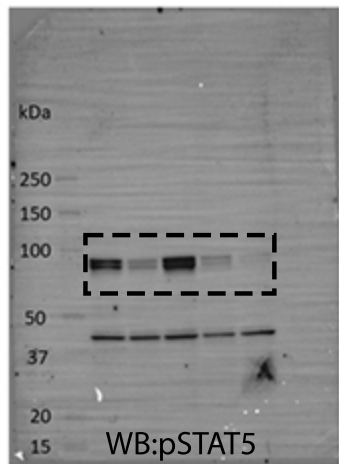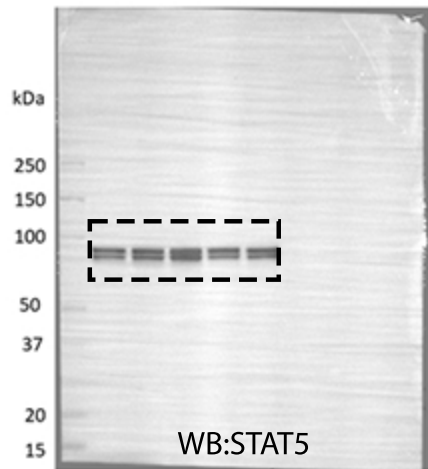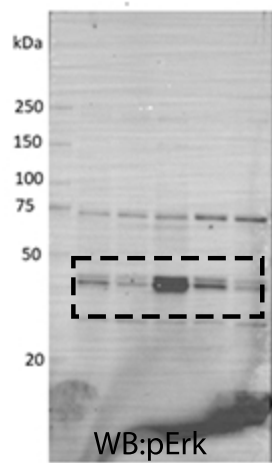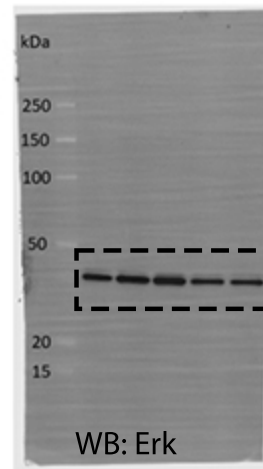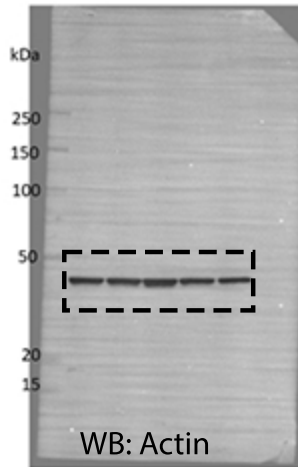

Above blots belong to Figure 4 Panel F and placed in the same order as in panel

Supplement: Supplementary file 5 — Source Data for Figure 4 [file EMBR-23-e52904-s003.pdf]
