# Supplementary material for: Targeting human CALR‐mutated MPN progenitors with a neoepitope‐directed monoclonal antibody
Source: EMBO Rep. 2022 Feb 14;23(4):e52904. doi: 10.15252/embr.202152904 (PMC8982588; doi:10.15252/embr.202152904)
Supplement: Supplementary file 6 [file EMBR-23-e52904-s005.pdf]

EMBO reports

Targeting human CALR mutated MPN progenitors with a neoepitope-directed monoclonal antibody

Denis Tvorogov, Chloe Thompson-Peach, Johannes Foßelteder, Mara Dottore, Frank Stomski, Suraiya Onnesha, Kelly Lim, Paul Moretti, Stuart Pitson, David Ross, Andreas Reinisch, Daniel Thomas, and Angel Lopez
DOI: 10.15252/embr.202152904

Corresponding author(s): Daniel Thomas (daniel.thomas@adelaide.edu.au) , Angel Lopez (angel.lopez@sa.gov.au)

Review Timeline:

Submission Date:	10th Jun 21
Editorial Decision:	5th Jul 21
Revision Received:	8th Nov 21
Editorial Decision:	8th Dec 21
Revision Received:	23rd Dec 21
Editorial Decision:	20th Jan 22
Revision Received:	23rd Jan 22
Accepted:	26th Jan 22

Editor: Achim Breiling

Transaction Report:

(Note: With the exception of the correction of typographical or spelling errors that could be a source of ambiguity, letters and reports are not edited. Depending on transfer agreements, referee reports obtained elsewhere may or may not be included in this compilation. Referee reports are anonymous unless the Referee chooses to sign their reports.)

1st Editorial Decision **5th Jul 2021**

Dear Dr. Thomas,

Thank you for the submission of your research manuscript to EMBO reports. We have now received the reports from the three referees that were asked to evaluate your study, which can be found at the end of this email.

As you will see, the referees think that these findings are of interest. However, they have several comments, concerns and suggestions, indicating that a major revision of the manuscript is necessary to allow publication of the study in EMBO reports. As the reports are below, and all their points need to be addressed, I will not detail them here.

During cross-commenting, the referee further stated:

Referee #1: At minimum, the authors need to provide mechanistic insight for the findings they report in cell lines and primary MPN samples. The absence of the appropriate controls in Figure 2 (a point also raised by Reviewer 3) is a very basic experiment. Additional ex vivo experiments to determine if 4D7 binds mutant CALR at the cell surface (especially in primary MPN cells) and that it inhibits/disrupts the binding interaction of mutant CALR and MPL (binding interaction studies could be done in cell lines) are also key requirements.

Referee #2:
I think that the mechanism of action is particularly challenging because the role and detection of extracellular mutated CALR remains very elusive in the literature. Therefore, they should show that they can detect CALR at the cell surface with this antibody or another one and should prove that they disrupt the interaction with their antibody by FACS or immunoprecipitation at least in cell lines.

Referee #3:
At a very minimum the evidence for binding of the antoibody to cell surface CALR mutant in cells that have MPL and cells that do not should be provided, and for the former evidence that inhibition of MPL phosphorylation, signaling should be clear, as well as the issue with stabilization of plasma free mutant CALR versus shedding. Evidence of this on primary cells and/or some in vivo data in KI mice would be ideal.

Given the constructive referee comments, we would like to invite you to revise your manuscript with the understanding that all referee concerns must be addressed in the revised manuscript or in the detailed point-by-point response. Acceptance of your manuscript will depend on a positive outcome of a second round of review. It is EMBO reports policy to allow a single round of revision only and acceptance of the manuscript will therefore depend on the completeness of your responses included in the next, final version of the manuscript.

Revised manuscripts should be submitted within three months of a request for revision. We are aware that many laboratories cannot function at full efficiency during the current COVID-19/SARS-CoV-2 pandemic, and we have therefore extended our 'scooping protection policy' to cover the period required for full revision. Please contact me to discuss the revision should you need additional time, and also if you see a paper with related content published elsewhere.

When submitting your revised manuscript, please also carefully review the instructions that follow below.

PLEASE NOTE THAT upon resubmission revised manuscripts are subjected to an initial quality control prior to exposition to re-review. Upon failure in the initial quality control, the manuscripts are sent back to the authors, which may lead to delays. Frequent reasons for such a failure are the lack of the data availability section (please see below) and the presence of statistics based on n=2 (the authors are then asked to present scatter plots or provide more data points).

When submitting your revised manuscript, we will require:

1) a .docx formatted version of the final manuscript text (including legends for main figures, EV figures and tables), but without the figures included. Please make sure that changes are highlighted to be clearly visible. Figure legends should be compiled at the end of the manuscript text.

2) individual production quality figure files as .eps, .tif, .jpg (one file per figure), of main figures and EV figures. Please upload these as separate, individual files upon re-submission.

The Expanded View format, which will be displayed in the main HTML of the paper in a collapsible format, has replaced the Supplementary information. You can submit up to 5 images as Expanded View. Please follow the nomenclature Figure EV1, Figure EV2 etc. The figure legend for these should be included in the main manuscript document file in a section called Expanded View Figure Legends after the main Figure Legends section. Additional Supplementary material should be supplied as a single pdf file labeled Appendix. The Appendix should have page numbers and needs to include a table of content on the first page (with page numbers) and legends for all content. Please follow the nomenclature Appendix Figure Sx, Appendix Table

Sx etc. throughout the text, and also label the figures and tables according to this nomenclature.

For more details, please refer to our guide to authors:
http://www.embopress.org/page/journal/14693178/authorguide#manuscriptpreparation

See also our guide for figure preparation:
http://wol-prod-cdn.literatumonline.com/pb-assets/embo-site/EMBOPress_Figure_Guidelines_061115-1561436025777.pdf

3) a .docx formatted letter INCLUDING the reviewers' reports and your detailed point-by-point responses to their comments. As part of the EMBO Press transparent editorial process, the point-by-point response is part of the Review Process File (RPF), which will be published alongside your paper.

4) a complete author checklist, which you can download from our author guidelines (https://www.embopress.org/page/journal/14693178/authorguide). Please insert page numbers in the checklist to indicate where the requested information can be found in the manuscript. The completed author checklist will also be part of the RPF.

Please also follow our guidelines for the use of living organisms, and the respective reporting guidelines:
http://www.embopress.org/page/journal/14693178/authorguide#livingorganisms

5) that primary datasets produced in this study (e.g. RNA-seq, ChIP-seq, structural and array data) are deposited in an appropriate public database. If no primary datasets have been deposited, please also state this a dedicated section (e.g. 'No primary datasets have been generated and deposited'), see below.

See also: http://embor.embopress.org/authorguide#datadeposition

Please remember to provide a reviewer password if the datasets are not yet public.

The accession numbers and database should be listed in a formal "Data Availability " section (placed after Materials & Methods) that follows the model below. This is now mandatory (like the COI statement). Please note that the Data Availability Section is restricted to new primary data that are part of this study.

Data availability

The datasets produced in this study are available in the following databases:

- RNA-Seq data: Gene Expression Omnibus GSE46843 (https://www.ncbi.nlm.nih.gov/geo/query/acc.cgi?acc=GSE46843)
- [data type]: [name of the resource] [accession number/identifier/doi] ([URL or identifiers.org/DATABASE:ACCESSION])

*** Note - All links should resolve to a page where the data can be accessed. ***

Moreover, I have these editorial requests:

6) We strongly encourage the publication of original source data with the aim of making primary data more accessible and transparent to the reader. The source data will be published in a separate source data file online along with the accepted manuscript and will be linked to the relevant figure. If you would like to use this opportunity, please submit the source data (for example scans of entire gels or blots, data points of graphs in an excel sheet, additional images, etc.) of your key experiments together with the revised manuscript. If you want to provide source data, please include size markers for scans of entire gels, label the scans with figure and panel number, and send one PDF file per figure.

7) Our journal encourages inclusion of *data citations in the reference list* to directly cite datasets that were re-used and obtained from public databases. Data citations in the article text are distinct from normal bibliographical citations and should directly link to the database records from which the data can be accessed. In the main text, data citations are formatted as follows: "Data ref: Smith et al, 2001" or "Data ref: NCBI Sequence Read Archive PRJNA342805, 2017". In the Reference list, data citations must be labeled with "[DATASET]". A data reference must provide the database name, accession number/identifiers and a resolvable link to the landing page from which the data can be accessed at the end of the reference. Further instructions are available at: http://www.embopress.org/page/journal/14693178/authorguide#referencesformat

8) Regarding data quantification and statistics, can you please specify, where applicable, the number "n" for how many independent experiments (biological replicates) were performed, the bars and error bars (e.g. SEM, SD) and the test used to calculate p-values in the respective figure legends. Please provide statistical testing where applicable, and also add a paragraph detailing this to the methods section. See:
http://www.embopress.org/page/journal/14693178/authorguide#statisticalanalysis

9) Please also note our new reference format:

http://www.embopress.org/page/journal/14693178/authorguide#referencesformat

10) For microscopic images, please add scale bars of similar style and thickness to all the microscopic images, using clearly visible black or white bars (depending on the background). Please place these in the lower right corner of the images. Please do not write on or near the bars in the image but define the size in the respective figure legend.

11) Please add a conflict-of-interest statement to the manuscript, up to five key words to the title page and order the manuscript sections like this:
Title page - Abstract - Introduction - Results - Discussion - Materials and Methods -Data availability section - Acknowledgements - Author contributions - Conflict of interest statement - References - Figure legends - Expanded View Figure legends.

Finally, please note that all corresponding and co-corresponding authors are required to supply an ORCID ID for their name upon submission of a revised manuscript. Please find instructions on how to link the ORCID ID to the account in our manuscript tracking system in our Author guidelines: http://www.embopress.org/page/journal/14693178/authorguide#authorshipguidelines

I look forward to seeing a revised version of your manuscript when it is ready. Please let me know if you have questions or comments regarding the revision.

Yours sincerely

Achim Breiling
Editor
EMBO Reports

Referee #1:

In an original research article to EMBO Reports, Tvorogov et al. report their findings on the development and testing of a mutant CALR specific monoclonal antibody (4D7). They present studies testing 4D7 in cell lines and primary MPN samples which they believe suggest therapeutic potential for this antibody in the treatment of CALR-mutant myeloid blood cancers.
Overall, this is a novel and interesting study.
However, there are substantial deficiencies in the work presented in terms of understanding the mechanism by which 4D7 has activity in the cell lines and MPN samples. The entire discussion of how 4D7 inhibits cell growth is not backed up by data to support the proposed mechanism of action. Specifically, the following are lacking:
1. The authors to do not demonstrate that 4D7 binds to mutant CALR on the cell surface when it is complexed to MPL e.g. using flow cytometry, immunofluorescence, microscopy or any imaging modality
2. The authors suggest that 4D7 binds the mutant CALR-MPL complex & disrupts it but they do not show any data to support this e.g. using co-localization assays such as BRET
3. The authors do not show that MPL phosphorylation is diminished / inhibited in the presence of treatment with 4D7
Other comments:
1. Several controls are lacking in Figure 2 e.g. TF-1 TpoR CALR-del52; TF-1 TpoR CALR-WT; TF-1 TpoR- JAK2V617F
2. It would also be informative to mutate the epitope to which 4D7 is proposed to bind and show that cell lines expressing this form of mutant CALR are resistant to 4D7
3. All assays are ex vivo. Studies in mutant CALR knockin mice harboring the human mutant CALR C-terminus or in patient-derived xenograft mutant CALR models would significantly improve the case for 4D7 as an antibody with therapeutic potential
Comments in the discussion
On page 12, the authors state:
"Physiologically, wild type CALR protein is normally present as a ligand for the "don't eat me" CD47 signal protecting cells from phagocytosis (Liu et al., 2020; Majeti, 2011; Majeti et al, 2009). It will be intriguing to test anti-CALR monoclonal antibody efficacy in the setting of anti-CD47 blocking therapies as a complementary strategy".
I would draw the author's attention to a publication from 2016 entitled "Calreticulin mutation does not contribute to disease progression in essential thrombocythemia by inhibiting phagocytosis" (PMID: 27185380) and suggest they modify this sentence of the discussion accordingly.

Referee #2:

Myeloproliferative neoplasms are clonal hematological diseases that lead to overproduction of myeloid cells. They are due to mutations in molecules that activate the JAK2/STAT pathway. CALR mutations (CALRmut) are one of these mutations that lead to a protein with a new C-terminal tail and loss of endoplasmic reticulum retrieval motif. These mutants bind to the MPL receptor at the cell surface and activate it. However, their mechanism of action remains incompletely understood and targeted therapy does not exist either.
In this study, the authors have generated a monoclonal antibody against the mutated tail of CALRmut and have investigated its

role in targeting CALRmut cell lines and primary cells from patients. They showed that this strategy selectively targets the mutated cells in contrast to normal cells. Their study indicates that this targeting is mainly due to inhibition of cell signaling mediated by CALRmut associated with an increased in CALRmut release in the medium probably due to the disruption of the CALRmut/MPL complex by the antibody.My main points concern the mechanism of action of the antibody on the MPL/CALRmut complex.

Overall, this is a very original study and valuable work that is well conducted with state-of-the-art techniques. The interest also resides from the experiments performed using primary cells that increases the relevance to the pathology.

Major:
My main points concern the mechanism of action of the antibody on the MPL/CALRmut complex. The authors suggest that their antibody could induce the dissociation of the CALRmut/MPL complex.
First of all, I wondered if the monoclonal mouse antibody against CALRmut from dianova may have similar effect since it also recognizes the mutant form.
Second, it is possible to detect the cell surface CALRmut by flow cytometry with the 4D7 antibody used in indirect labeling?
Third, it would be important to show that 4D7 can displace the MPL/CALRmut complex at the cell surface by performing a co-immunoprecipitation of cell surface MPL/CALRmut in the presence or not of 4D7. Alternatively, authors could also show that 4D7 can inhibit the expression of CALRmut at the cell surface by using another antibody directed against N-ter CALR.

The authors have attempted to identify an increase in apoptosis of TF1-MPL-CALRmut cell lines in the presence of 4D7 by indicating the % of cells in sub-G0. However, in view of the figure EVF2B, the indicated sub-G0 is not correct and rather seems to represent dead cells. The authors might want to check other markers such as annexin V and/or cleaved caspase 3.

Last but not least, the authors should cite the original papers in their introduction instead of general reviews.
For instance, authors should cite Klampfl, NEJM, 2013 and Nangalia, NEJM, 2013 for CALR mutations discovery. Then for the CALRmut-mediated MPL interaction and activation mechanism (Araki et al., Blood, 2016, Chachoua et al., Blood, 2016, Marty et al., Blood, 2016, Elf et al., Cancer discovery, 2016).... Etc...
Araki et al., Leukemia 2019 could also be discussed in term of CALR multimerization and effect on the antibody 4D7.

Minor:

The authors should provide the % of CD41+ cells they obtained in their megakaryocytic liquid conditions and provide examples of flow cytometry analysis with Ig and 4D7.
Did the authors test the 4D7 effect in the presence of TPO?

Referee #3:

Tvogorov and colleagues developed an antibody against the mutant C-terminus of calreticulin. They show that this antibody is able to inhibit CALR mutant dependent proliferation and activation of the TpoR in cell lines and certain primary cells. There is no in vivo testing and major questions remain about the actual mechanism of action of this new antibody, which should be probed in vivo as well. Certain experiments are over-interpreted and others go in many directions not relevant to the main issue of how, where and by which mechanism does this new antibody target the CALR mutant-TPOR complex. The authors should also be cautious about important side effects such antibody may have in vivo as extracellular calreticulin is known to bind to cell surface in vivo - which could lead to killing of healthy cells in vivo, if an immune mechanism is at play. At this stage the data is interesting but too early to transmit any other message aside from the isolation of an antibody to a novel epitope that in vitro appears to inhibit proliferation. Important matters should be addressed, namely mechanism and in vivo effects and conclusions should only be drawn from experimental proof without over -interpretation.

The following comments should be mandatory for publication:
1) The authors show (although there are some very misleading problems in Figure 1, see below) that 4D7 is a monoclonal rat antibody that binds with high affinity to the proximal region of the CALR del61 mutant sequence. Later, in Fig. 1D, they seem to show that 4D7 enhances the levels of CALR in the conditioned media. In my opinion, additional experiments are required in order to state that 4D7 has an effect on CALR secretion (see title of paragraph on page 7). It could simply be that 4D7 stabilizes the CALR mutant protein in the medium, increasing its half-life. Others have shown that CALR del52 is quite an unstable protein 1. While the claim is that shedding of CALR del52 occurs from the surface of the cells, there is no corresponding decrease of CALR deL52 in the cell extracts. Two mandatory questions are whether i) the antibody stabilizes extracellular CALR? ii) the amount of CALR at the cell surface is or not decreased after antibody treatment. These are critical questions to answer to demonstrate the mechanisms of action of this antibody. An alternative view is that the antibody acts by either sequestrating extracellular CALR or by binding to surface CALR - without inducing shedding - and thereby disrupting activation of the TpoR. These questions must be answered.
2) There are several important problems with Figure 1, Panel A that need to fixed:
The mutant sequences (in red) have a lot of Glutamates (E) that should in fact by Arginines (R) ! (MEMEE... should be

MRMRR...); The blue "C-term" underline and Orange "N-term" underlines should be reversed. (In panel B the colors are correct); The red lines connecting the injected peptide sequence to the ins5 mutant sequence should be adjusted, so that the "MMRTK" of the peptide connects to the "MMRTK" of the ins5 sequence; Same goes for the end of the peptide, the red line should point to the "PARPRTS" in the ins5 sequence; Either show CALR del61 sequence next to del52 or state that the immunogenic peptide corresponds to CALR del61 sequence.

3) In Figure 2, the authors show that 4D7 is capable of inhibiting the growth of TpoR-expressing CALR del61 mutated TF-1 cells, but not cells expressing TpoR alone or SET2 JAK2 V617F cells. A couple of concerns here: i) Why only test the effect of 4D7 on TF-1 TpoR CALR del61? I understand that del61 is the same mutation as found in the MARIMO cell-line but as the authors point themselves out: the CALR mutation in the MARIMO cells is not (or no longer) the proliferation driving mutation of those cells. While the del61 mutation closely resembles the del52 mutation of CALR, it lacks 3 amino-acids at the very proximal end of the mutant sequence compared to del52. Morevoer, the del52 mutation is one of two most prevalent form of mutations found in patients (the other being the ins5 mutation). These observations repeated and confirmed using the TF-1 TpoR CALR del52 mutant; ii) The JAK2 V617F mutant cells are SET2 and not TF-1. These two cell-lines are quite different. In order to conclude from a direct comparison, the authors should show TF-1 cells transduced to express JAK2 V617 and show that these cells also resist to 4D7 treatment. Later the authors nicely show that the STAT5 and ERK signaling are impaired in the TF-1 TpoR CALR del61 cells. It would be also of interest to see the levels of pSTAT1 and pSTAT3 as a comparison. Indeed, TpoR is known for signaling also through STAT3 and also a bit through STAT1.

4) A critical question is whether this antibody exerts its effect through disruption of the CALR-TpoR interaction, therefore preventing the differentiation bias, or does it induce some form of cytotoxicity to the CALR mutant cells? The authors mention trypan blue exclusion of dead cells in their experiment strategy but do not show any numbers.

5) Have the authors tested whether 4D7 can be used to assess the presence/absence of mutant CALR at the cell surface through FACS? This would be a good indicator that the antibody can access CALR at the cell surface. Even though 4D7 may disrupt the interaction of CALR with TpoR a fraction should still be bound to the cell surface and allow for detection through FACS

6) The authors performed co-culture studies with TF-1 TpoR and TF-1 TpoR CALR del61 to assess a paracrine effect of CALR mutant. In this study, similar to Araki 2016, the authors simply measure the proliferation of TF-1 TpoR versus TF-1 TpoR CALR del61 present in the same culture. As expected, TF-1 TpoR CALR del61 cells have a strong competitive advantage and proliferate at the expense of TF-1 TpoR cells not expressing the CALR mutant (Fig EV1D). From this, the authors conclude that CALR mutants do not exert a paracrine effect. This is clearly an over-interpretation. The fact that CALR expressing cells have a stronger proliferation - especially in the same culture flask- does not show an absence of paracrine effect of mutant CALRThe authors should revise this part or provide robust additional data to prove their assumptions. Although this is not the main subject of this paper, this is an obvious misinterpretation of data.

7) Perhaps the strong results in this paper reside with experiments performed on patient-derived cells. The patient-derived cells, the authors show that the 4D7 antibody has a clear efficacy to inhibit the proliferation and differentiation of CALR mutated hematopoietic cells in vitro. Once more: the majority of the patient derived cells have the del52 CALR mutation; this contrasts with the TF-1 cell-line experiments that were expressing the CALR del61 mutant.

8) It is not very clear what Figure 5, panel B brings as novel point. Also, it is known and previously published that Brefeldin A (Pecquet et al. 2019) prevents the secretion of CALR. Can the authors explain more clearly the significance of the panel. In addition, some groups have shown that not only CALR mutant interact with TpoR to activate the JAK-STAT pathway, the mutant also disrupts the calcium homeostasis of megakaryocytes (Di Buduo et al. Blood 2018, 132, 1782. Experiments exploring these questions would have been probably more impactful.

9) In the last section, the author suggest that they are able to target the "fragile CALR-TpoR-glycosylation complex". This appears to be contradicting the fact that their antibody targets the mutant c-terminus. How do they explain that their antibody targets the tail and would still be able to disrupt the glycosylation binding?

10) The last two sentences of the last paragraph of the Discussion are simply wrong: Wild-type CALR acts as a "eat me" signal at the surface of stressed cells, and is the ligand of LRP1R that is present on the surface of macrophages. CALR is not the ligand to CD47. CD47 is a ubiquitously expressed protein and acts as a constitutive "Don't eat me" signal at the cell surface. CD47 binds the SIRPα on the surface of macrophage to convey that signal.

11) A number of key references are missing or not correctly cited. The data on requirement of the N-domain of CALR mutants and of the tail for TPOR activation are in Chachoua et al. Blood. 2016;127:1325-1335; interaction and traffic are in Masubuchi et al. Leukemia. 2020 Feb;34(2):499-509. also shown the requirement for oligomerization of mutant CALR is in Araki et al. Leukemia. 2019 Jan;33(1):122-131.

12) The authors should discuss how the cleavage of the last portion of the C-tail (including the epitope) recently reported at ASH impacts the binding of the antibody.

In conclusion, the manuscript offers encouraging in vitro data of the capacity of the 4D7 monoclonal rat antibody to prevent the proliferation and differentiation of CALR mutated cells. It stops short of offering a mechanistic explanation of how the antibody

exerts this effect and the in vivo effects are not explored. Some claims of the manuscript are stretched and should be readjusted (see comments hereabove). There are some problems with the first panel of the first figure and two sentences at the end of the discussion that absolutely need to be corrected before considering further this manuscript. The major weakness beyond the lack of mechanism remains absence of any in vivo data, and this should be addressed as it would help also the establishment of mechanism.

We thank the reviewers for their helpful comments and are particularly pleased with the editor's comments that "the findings are of interest" and reviewer #1 "overall, this is a novel and interesting study." We appreciate reviewer #2's comments that "this is a very original study and valuable work with state-of-the-art techniques" and "experiments performed using primary cells increases relevance "and reviewer #3's comment that the data is "interesting"

We submit here a point-by-point response to the comments.

Reviewer #1

Overall, this is a novel and interesting study. The entire discussion of how 4D7 inhibits cell growth is not backed up by data to support the proposed mechanism of action. Specifically, the following are lacking: (1)The authors to do not demonstrate that 4D7 binds to mutant CALR on the cell surface when it is complexed to MPL e.g. using flow cytometry, immunofluorescence, microscopy or any imaging modality.

Thankyou for this important point. By conjugating 4D7 to phycoerythrin we have now confirmed strong binding to the cell surface of mutant CALR cells by flow cytometry (new **Fig 1D**) without permeabilization, that is not present in endogenous CALR wild type cells or isotype IgG-PE control (**Fig EV1E**).

2. The authors suggest that 4D7 binds the mutant CALR-MPL complex & disrupts it but they do not show any data to support this e.g. using co-localization assays such as BRET.

Using non-reducing conditions and thrombopoietin receptor immunoprecipitation we have confirmed that 4 hour treatment with 4D7 diminishes both mutant CALR dimers and monomers bound to TpoR (new **Figures 2F** and **EV4A**). We have updated our model (new **Fig 2G**) and discussion to reflect this.

3. The authors do not show that MPL phosphorylation is diminished / inhibited in the presence of treatment with 4D7.

This is an important point. We have now confirmed that TpoR phosphorylation is constitutively active in the presence of mutant CALR and this blocked by specifically by 4D7 but not by IgG or PBS (new **Fig EV3F**). These data confirm previous reports that mutant CALR is able to dimerize and phosphorylate TpoR in the absence of TPO and is consistent with our diminished downstream pSTAT1, pSTAT3, pSTAT5 and pERK signalling data showing inhibition by 4D7 (**Fig2 A,B,C** and **EV3 A, B, C**).

4. Several controls are lacking in Figure 2 e.g. TF-1 TpoR CALR-del52; TF-1 TpoR CALR-WT; TF-1 TpoR- JAK2V617F.

We have added all these important controls. We performed a number of new experiments and added proliferation data for $CALR^{WT}$, cytokine-dependent TF-1 $CALR^{del61}$ and TF-1 $CALR^{del52}$, and $PTPN11^{E76K}$ (recurrent mutation in myelofibrosis)(new **Fig EV2 A, B, C, F**). MARIMO and SET2 controls are also shown, as previously (**Fig EV2D** and **E**). Additionally, the biologically significant TF-1 TpoR $CALR^{del52}$ TPO-independent cell line was also generated (new **Fig 1G, Fig 2C, Fig EV3C**). All experiments underscore the specificity of 4D7 for mutant CALR only and only in cells where both mutant CALR and TpoR are co-expressed.

2. It would also be informative to mutate the epitope to which 4D7 is proposed to bind and show that cell lines expressing this form of mutant CALR are resistant to 4D7.

Proper determination of the binding epitope requires a 3-dimensional crystal structure of mutant CALR protein in complex with the mAb (and possibly also in complex with extracellular portion of TpoR), which is outside the scope of this short report. To this end, we have partially defined the epitope to likely encompass at least an 11 amino acid sequence comprising CMMRTKMRMRR (**Fig 1B**), based on peptide binding assays compared to full length neoepitope peptide. However, a peptide binding assay may not reflect the actual structure present in cells with mutant CALR protein dimers complexed to TpoR.

We attempted to further map the epitope by mutating the positively charged arginine and lysine within this N-terminal sequence to alanine. However, this mutant form of CALR did not confer factor-independence in our hands, even when expressed in combination with TpoR (see figure below), suggesting that these residues are perhaps critical for TPO-independent cell growth and thus biological activity of 4D7 cannot be properly assessed. Further work is required to elucidate the role of this putative epitope in 4D7 binding with more extensive mutagenesis studies.

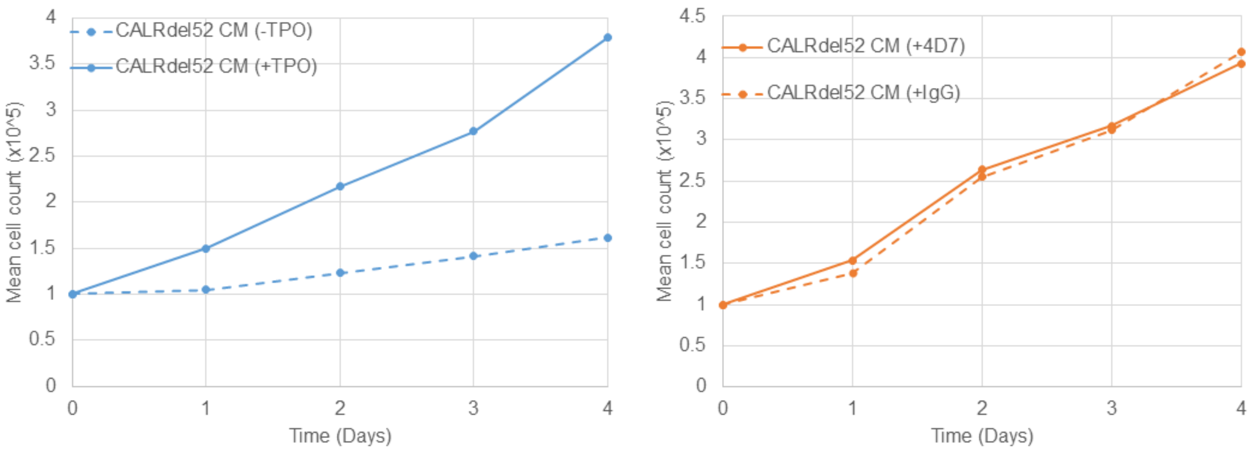

3. All assays are ex vivo. Studies in mutant CALR knockin mice harboring the human mutant CALR C-terminus or in patient-derived xenograft mutant CALR models would significantly improve the case for 4D7 as an antibody with therapeutic potential.

Agreed. Whilst a knock-in model would be beneficial, our data have shown that the human TPO receptor is necessary for the pathogenic effect of the CALR mutation. Therefore, it would be necessary to generate a mouse model that not only expresses mutant CALR, but also the human TPO receptor.

Instead, we have now included 3 independent xenograft in vivo experiments accompanied by extensive pharmacokinetic data for single intraperitoneal administration of 4D7 in NSG mice (new **Fig 5 A to G**). All three models show a decrease in growth of mutant CALR driven myeloproliferation in a human cell-line model suggesting the antibody may have efficacy in slowing disease progression.

In detail, we developed two distinct xenograft models, a bone marrow engraftment model, which measures mutant *CALR* dependent proliferation in the bone marrow microenvironment, and a chloroma model, which mimics extramedullary infiltration of mutant *CALR* leukemia. In the bone marrow engraftment model (new **Fig 5A**), 4D7 treatment (12.5 mg/kg twice weekly via intraperitoneal injection starting day 7) showed excellent pharmacokinetic profile, achieving more than 100 µg/mL 48 hours post injection (**Fig 5B**) and lowered peripheral blood engraftment of human CD33 myeloid cells at 3 weeks (0.04 vs 19.8 %CD33 4D7 vs IgG, *P* = 0.001) (new **Fig 5C**) and significantly prolonged survival (log-rank hazard ratio 0.24, *P* = 0.003) (new **Fig 5D**). In the chloroma model, 4D7 treatment significantly slowed tumor growth at 21 days post engraftment (353 vs 3317 mm^3 mean tumor volume, 4D7 vs IgG, *P* = 0.03) (new **Fig 5E**) and prolonged survival (hazard ratio

0.19, *P* = 0.026) (new **Fig 5F**). Strikingly, mutant CALR cells induced to be resistant to 100 nM ruxolitinib also showed a survival advantage after treatment with 4D7 (12.5 mg/kg twice weekly beginning at day 7) in bone marrow engraftment model (hazard ratio 0.26, *P* = 0.005) (new **Fig 5G**). Together, these results suggest an immunotherapeutic approach may have clinical utility *CALR*-driven myeloproliferative neoplasms and *CALR* mutant acute myeloid leukemia, as well as activity in *CALR* mutant patients that develop resistance/persistence to ruxolitinib.

Page 12, I would draw the author's attention to a publication from 2016 entitled "Calreticulin mutation does not contribute to disease progression in essential thrombocythemia by inhibiting phagocytosis" (PMID: 27185380) and suggest they modify this sentence of the discussion accordingly.

This has been modified and the appropriate reference has been included.

General comments by reviewer #1. At minimum, the authors need to provide mechanistic insight for the findings they report in cell lines and primary MPN samples. The absence of the appropriate controls in Figure 2 (a point also raised by Reviewer 3) is a very basic experiment. Additional ex vivo experiments to determine if 4D7 binds mutant CALR at the cell surface (especially in primary MPN cells) and that it inhibits/disrupts the binding interaction of mutant CALR and MPL (binding interaction studies could be done in cell lines) are also key requirements.

We believe all of these controls and mechanistic experiments have been performed as outlined in detail above.

Reviewer #2

My main points concern the mechanism of action of the antibody on the MPL/CALRmut complex. The authors suggest that their antibody could induce the dissociation of the CALRmut/MPL complex. First of all, I wondered if the monoclonal mouse antibody against CALRmut from dianova may have similar effect since it also recognizes the mutant form.

We tried to use commercially obtained Dianova mouse monoclonal IG2a antibody (Clone CAL2). This preparation contains sodium azide 0.05% and the exact concentration is not stated in the accompanying datasheet. It is difficult to compare selective inhibition without an azide-free sterile preparation and exact concentration to compare with our dose-response for 4D7, a rat monoclonal.

Second, it is possible to detect the cell surface CALRmut by flow cytometry with the 4D7 antibody used in indirect labeling?

We agree this is a critical experiment. By conjugating 4D7 to phycoerythrin we have now confirmed strong binding to the cell surface of mutant CALR cells by flow cytometry (new **Fig 1D**) without permeabilization, that is not present in CALR wildtype cells or isotype IgG-PE control (new **Fig EV1E**). This data is consistent with 4D7 disrupting mutant CALR protein dimers bound to TpoR localized predominantly at the cell surface, suggesting that the antibody is able to bind and modulate cell surface protein on CALR mutant cells.

Third, it would be important to show that 4D7 can displace the MPL/CALRmut complex at the cell surface by performing a co-immunoprecipitation of cell surface MPL/CALRmut in the presence or not of 4D7.

Excellent suggestion. We have done this experiment as shown in new **Fig 2F** with extra controls in reducing conditions in **Fig EV3E** and **Fig EV4A**. As outlined above, we used non-reducing conditions and thrombopoietin receptor immunoprecipitation to confirm that treatment with 4D7 disrupts the binding of mutant CALR monomers and disulfide-linked dimers bound to TpoR. We have updated our model and discussion to reflect this.

The authors have attempted to identify an increase in apoptosis of TF1-MPL-CALRmut cell lines in the presence of 4D7 by indicating the % of cells in sub-G0. However, in view of the figure EVF2B, the indicated sub-G0 is not correct and rather seems to represent dead cells. The authors might want to check other markers such as annexin V and/or cleaved caspase 3.

Thank you for this suggestion. We have added a caspase 3 cleavage Western blot (new **Fig 2E**) to clarify this which indeed shows apoptosis beginning within 24 hours of 4D7 in 10% serum of TPO-independent TF-1 TpoR $CALR^{del\ 61}$ and TF-1 TpoR $CALR^{del52}$ cells. Additionally, various other studies have used the sub-G0 phase of the cell cycle to show apoptosis (PMID: 21196218, PMID: 24029497, PMID: 33171654, PMID: 31351461) and as such, these data have been left in (**Fig 2D**, **Fig EV3D**).

Last but not least, the authors should cite the original papers in their introduction instead of general reviews.

All of these references have been added in the revised introduction.

The authors should provide the % of CD41+ cells they obtained in their megakaryocytic liquid conditions and provide examples of flow cytometry analysis with Ig and 4D7.

This has been added to new **Fig EV4C**

Did the authors test the 4D7 effect in the presence of TPO?

Yes, we see partial reversal but not complete reversal, suggesting TpoR in the presence of mutant CALR may have a distinct binding configuration, in keeping with recent reports (PMID: 34010413). These findings have not been included in the current manuscript as it suggests an entirely new and exciting direction of research.

General comments from Referee #2: I think that the mechanism of action is particularly challenging because the role and detection of extracellular mutated CALR remains very elusive in the literature. Therefore, they should show that they can detect CALR at the cell surface with this antibody or another one and should prove that they disrupt the interaction with their antibody by FACS or immunoprecipitation at least in cell lines.

We agree that the nature of the mutant CALR is not clear and as a result, this has led to great difficulty in understanding the mechanism of action of 4D7. We believe we have sufficiently addressed these critical points.

Reviewer #3

Tvogorov and colleagues developed an antibody against the mutant C-terminus of calreticulin. They show that this antibody is able to inhibit CALR mutant dependent proliferation and activation of the TpoR in cell lines and certain primary cells. There is no in vivo testing and major questions remain about the actual mechanism of action of this new antibody, which should be probed in vivo as well.

We have added 3 independent in vivo experiments (new **Fig 5 A-G**) and additional mechanistic data that support a mutation-specific mechanism of action interfering with the TpoR-CALR complex at the cell surface (new **Figs 1D, 2F, 2G, EV1E, EV3E, EV3F, EV4A**).

Certain experiments are over-interpreted and others go in many directions not relevant to the main issue of how, where and by which mechanism does this new antibody target the CALR mutant-TpoR complex.

We agree our primary submission was not able to clarify the mechanism of action despite interesting biological observations. We have therefore revised our abstract and conclusions to reflect the new data we now have to support both efficacy and mechanism.

The authors should also be cautious about important side effects such antibody may have in vivo as extracellular calreticulin is known to bind to cell surface in vivo - which could lead to killing of healthy cells in vivo, if an immune mechanism is at play.

We agree. We have included new pharmacokinetic data and 3 different in vivo experiments in NSG mice using human CALR-driven myeloproliferation. Mice tolerated the treatments well at high dose (12.5 mg/kg) with serum concentrations exceeding 100 µg/mL at 48 hours post-administration. We have revised our discussion to include a statement about potential toxicity.

At this stage the data is interesting but too early to transmit any other message aside from the isolation of an antibody to a novel epitope that in vitro appears to inhibit proliferation. Important matters should be addressed, namely mechanism and in vivo effects and conclusions should only be drawn from experimental proof without over -interpretation.

We have modified our conclusions in the abstract and discussion to reflect our revised data. We agree that it is premature to conclude that such an approach would be effective in the clinic without further testing.

In my opinion, additional experiments are required in order to state that 4D7 has an effect on CALR secretion (see title of paragraph on page 7). It could simply be that 4D7 stabilizes the CALR mutant protein in the medium, increasing its half-life. Others have shown that CALR del52 is quite an unstable protein.

This is an important consideration. We have performed experiments using 4D7 vs IgG in total cell extracts and TpoR IP in non-reducing conditions, using an anti-mutant CALR antibody (Dianova) and anti-wild type CALR antibody (Cell Signalling) (new **Fig 2F, EV4A**). Importantly, we do not see any cleavage of CALR protein bound to TpoR after immunoprecipitation or in total cell lysates after 4D7 treatment.

We do see eventual cleavage of CALR in conditioned media with a decrease noted after 4D7 exposure for 12 hours, in keeping with recent ASH abstract Kihari et al #716, 2020. This cleavage product was observed only with anti-wild type CALR antibody, see reubuttal figure below. We do not believe that CALR cleavage is likely to be the major mechanism of action of 4D7 because we do not see this phenomenon in TpoR IP or lysates.

While the claim is that shedding of CALR del52 occurs from the surface of the cells, there is no corresponding decrease of CALR deL52 in the cell extracts. Two mandatory questions are whether i) the antibody stabilizes extracellular CALR? ii) the amount of CALR at the cell surface is or not decreased after antibody treatment. These are critical questions to answer to demonstrate the mechanisms of action of this antibody.

We appreciate the insightful comments. As already mentioned, we now have convincing data that shows that (i) bound mutant CALR dimers and monomers associated with TpoR are reduced after 4D7 treatment (new **Fig 2F**) resulting in (ii) decreased TpoR phosphorylation (**Fig EV3F**) and (iii) decreased STAT1, STAT3 and STAT5 phosphorylation (new **Fig EV3 A, B, C**). Furthermore, our data suggests the CALR protein as monomers and

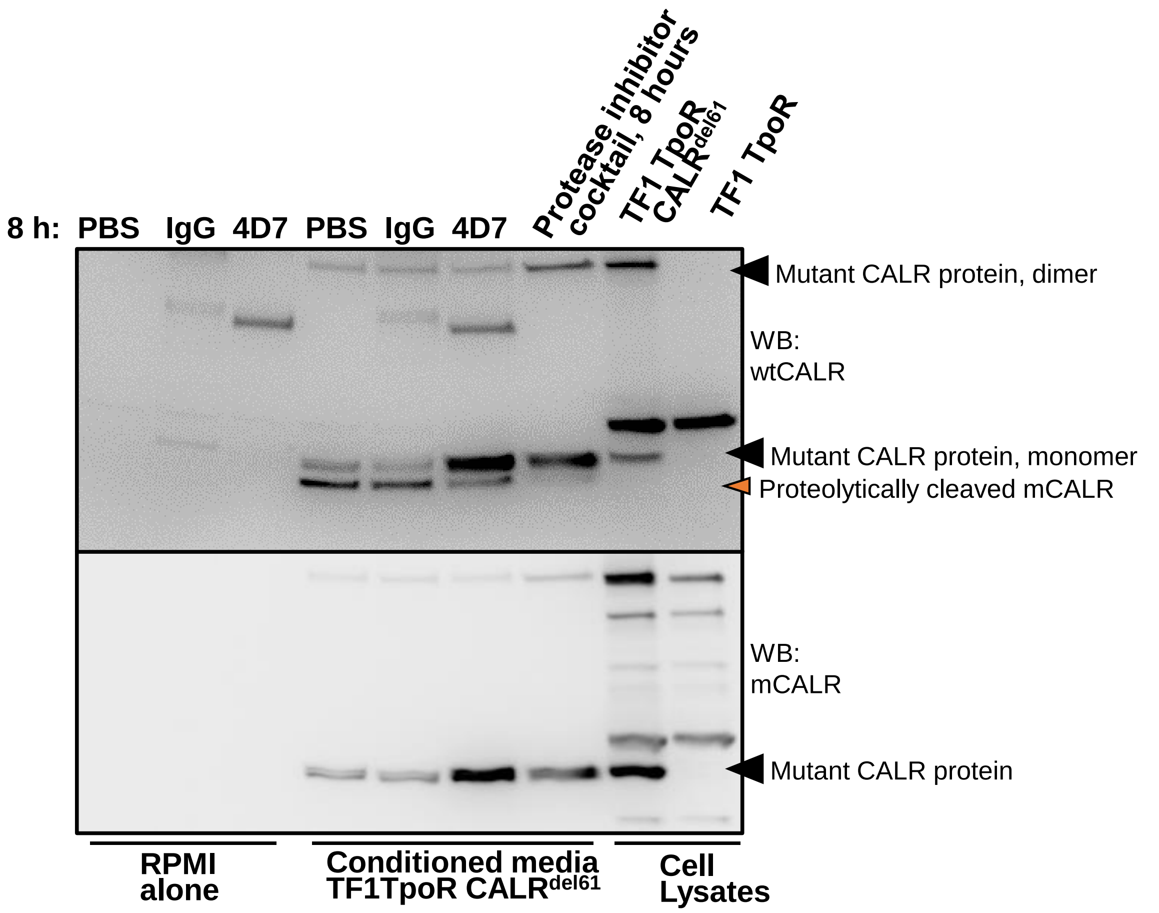

4D7 prevents cleavage of mutant CALR protein in condition media over 8 hours, detectable only by anti-wild type CALR antibody (Cell Signalling). Conditioned media from TF-1 cells expressing mutant CALR^{del61} was incubated for 8 hours in presence of PBS control, 20 µg/mL IgG control or 20 µg/mL 4D7 mAb. A cleavage product only detectable by Western blot with anti-wild type CALR antibody (Cell Signalling) was noted after 8 hours that was not present in cell lysates, suggesting proteolytic cleavage occurs only outside cell. Cleavage product was blocked by protease inhibitor cocktail, consistent with the action of a protease. Both panels are non-reducing gels. 4D7 decreased the amount of cleaved monomer, leading to an increase in total monomer protein evident. RPMI alone did not show any mutant CALR protein. Mutant CALR is not observed in

disulfide linked dimers bound to TpoR present in non-reducing conditions. Regarding stabilization of extracellular by 4D7, we now have convincing data that this is indeed occurring in conditioned media but unlikely to be responsible for TpoR signalling. As shown in the figure above with appropriate controls, we observed a cleavage fragment of CALR in

conditioned media but only after an extended period (8 hours). This cleavage product was prevented by protease inhibitors and diminished by 4D7 co-incubation. This cleavage was not present in cell lysates or TpoR immunoprecipitates, suggesting it is unrelated to the principal action of 4D7 on the cell surface. For this reason we have not included the data on cleavage in the main manuscript.

Our findings suggest that the amount of mutant CALR at the cell surface at any one time, non-covalently bound to TpoR, is probably much less than the total mutant CALR protein produced and trafficked through the rough endoplasmic reticulum plus any internalized TpoR/CALR from activation signaling complex because we see a minor decrease in total mutant CALR protein in lysates after 4D7 exposure (new **Fig 2F**) but we see a significant reduction of the CALR bound to TpoR.

An alternative view is that the antibody acts by either sequestrating extracellular CALR or by binding to surface CALR - without inducing shedding - and thereby disrupting activation of the TpoR. These questions must be answered.

As outline in rebuttal to reviewers #1 and #2, we agree that “shedding” is an oversimplification. Rather, there is clearly a disruption CALR/TpoR complex (new **Fig 2F**) that is normally required to for constitutive TpoR signaling in the absence of TPO. Thus, there is a blockage of TpoR/CALR internalization with decreased JAK and STAT phosphorylation and TpoR phosphorylation, and in the absence of internalization, excess monomeric CALR protein non-covalently bound to TpoR likely passively diffuses into the supernatant, rather than internalized. We have modified our model (new **Fig 2G**) and discussion to reflect this.

There are several important problems with Figure 1, Panel A that need to fixed: The mutant sequences (in red) have a lot of Glutamates (E) that should in fact by Arginines (R) ! (MEMEE... should be MRMRR...); The blue "C-term" underline and Orange "N-term" underlines should be reversed. (In panel B the colors are correct); The red lines connecting the injected peptide sequence to the ins5 mutant sequence should be adjusted, so that the "MMRTK" of the peptide connects to the "MMRTK" of the ins5 sequence; Same goes for the end of the peptide, the red line should point to the "PARPRTS" in the ins5 sequence; Either show CALR del61 sequence next to del52 or state that the immunogenic peptide corresponds to CALR del61 sequence.

We thank the reviewer for careful checking. We made an error when building the original schematic figure. This has all been corrected.

In Figure 2, the authors show that 4D7 is capable of inhibiting the growth of TpoR-expressing CALR del61 mutated TF-1 cells, but not cells expressing TpoR alone or SET2 JAK2 V617F cells. A couple of concerns here: i) Why only test the effect of 4D7 on TF-1 TpoR CALR del61? I understand that del61 is the same mutation as found in the MARIMO cell-line but as the authors point themselves out: the CALR mutation in the MARIMO cells is not (or no longer) the proliferation driving mutation of those cells. While the del61 mutation closely resembles the del52 mutation of CALR, it lacks 3 amino-acids at the very proximal end of the mutant sequence compared to del52. Morevoer, the del52 mutation is one of two most prevalent form of mutations found in patients (the other being the ins5 mutation).

Thank you for critical comments. We have added all these important controls. We performed a number of new experiments and added proliferation data for TF-1 TpoR $CALR^{del52}$, $CALR^{WT}$ and PTPN11 (recurrent mutation in myelofibrosis). All experiments underscore the specificity of 4D7 for mutant CALR only (new **Fig 1G, Fig EV2 A, B, C, E**) as well as additional primary samples with $JAK2^{V617F}$ mutations, which did not show response (new **Fig 3B**).

The JAK2 V617F mutant cells are SET2 and not TF-1. These two cell-lines are quite different. In order to conclude from a direct comparison, the authors should show TF-1 cells transduced to express JAK2 V617 and show that these cells also resist to 4D7 treatment.

We have performed experiments in primary patient MF CD34 cells with high VAF $JAK2^{V617F}$ which do not show response to 4D7. We have also shown no response to SET2 cells which are JAK2 mutation dependent. Several attempts were made to generate cytokine independent TF-1 JAK2V617F, however this was not successful. As an alternative, we have utilized cytokine independent TF-1 $PTPN11^{E76K}$ cells (a recurrent mutation in myelofibrosis), which also showed no response to 4D7 (**Fig EV2F**).

Later the authors nicely show that the STAT5 and ERK signaling are impaired in the TF-1 TpoR CALR del61 cells. It would be also of interest to see the levels of pSTAT1 and pSTAT3 as a comparison. Indeed, TpoR is known for signaling also through STAT3 and also a bit through STAT1.

These extra blots are provided in new **Fig EV3 A, B** and **C**.

4) A critical question is whether this antibody exerts its effect through disruption of the CALR-TpoR interaction, therefore preventing the differentiation bias, or does it induce some form of cytotoxicity to the CALR mutant cells? The authors mention trypan blue exclusion of dead cells in their experiment strategy but do not show any numbers.

We have shown disruption of CALR/TpoR interaction in new **Fig 2F** as stated. All proliferation curves show exact numbers of trypan negative cells. Our proliferation data demonstrates that 4D7 is a slow acting antibody and several days are required to see an effect. Due to the slower nature of this, minimal numbers of trypan blue positive cells are observed.

5) Have the authors tested whether 4D7 can be used to assess the presence/absence of mutant CALR at the cell surface through FACS? This would be a good indicator that the antibody can access CALR at the cell surface. Even though 4D7 may disrupt the interaction of CALR with TpoR a fraction should still be bound to the cell surface and allow for detection through FACS.

See comments to Reviewer #1. By conjugating 4D7 to PE we have now confirmed strong binding to the cell surface of mutant CALR cells by flow cytometry at 4 ug/ml (new **Fig 1D**) without permeabilization. Binding does not occur on endogenous CALR wild type cells or isotype IgG-PE control at the same concentration (**Fig EV1E**).

6) The authors performed co-culture studies with TF-1 TpoR and TF-1 TpoR CALR del61 to assess a paracrine effect of CALR mutant. In this study, similar to Araki 2016, the authors simply measure the proliferation of TF-1 TpoR versus TF-1 TpoR CALR del61 present in the same culture. As expected, TF-1 TpoR CALR del61 cells have a strong competitive advantage and proliferate at the expense of TF-1 TpoR cells not expressing the CALR mutant (Fig EV1D). From this, the authors conclude that CALR mutants do not exert a paracrine effect. This is clearly an over-interpretation. The fact that CALR expressing cells have a stronger proliferation - especially in the same culture flask- does not show an absence of paracrine effect of mutant CALR. The authors should revise this part or provide robust additional data to prove their assumptions. Although this is not the main subject of this paper, this is an obvious misinterpretation of data.

Agreed. We have modified our conclusion in this section and performed further comparison curves using a horizontal co-culture analysis with a semi-permeable membrane (0.75 micron) which allows protein but not cells to transfer between the chambers. This is reflected in new **Fig EV1D**. What is striking is that the TF-1 CALR^{del61} cells, although factor-independent in the absence of TPO, cannot provide a proliferative advantage to wild type TF-1 cells expressing in TpoR. Furthermore, in the presence of the same concentrations of TPO, the mutant CALR cells still show a slight proliferative advantage.

7) Perhaps the strong results in this paper reside with experiments performed on patient-derived cells. The patient-derived cells, the authors show that the 4D7 antibody has a clear efficacy to inhibit the proliferation and differentiation of CALR mutated hematopoietic cells in vitro. Once more: the majority of the patient derived cells have the del52 CALR mutation; this contrasts with the TF-1 cell-line experiments that were expressing the CALR del61 mutant.

We have engineered TF-1 TpoR CALR^{del52} cells and show similar results with this more common mutation in new **Fig 1G,** new **Fig 2C,** new **EV3C**, new **EV4A**.

8) It is not very clear what Figure 5, panel B brings as novel point. Also, it is known and previously published that Brefeldin A (Pecquet et al. 2019) prevents the secretion of CALR. Can the authors explain more clearly the significance of the panel.

We have removed this control and agree it has been shown by other groups. We have added the reference to make this point.

In addition, some groups have shown that not only CALR mutant interact with TpoR to activate the JAK-STAT pathway, the mutant also disrupts the calcium homeostasis of megakaryocytes (Di Buduo et al. Blood 2018, 132, 1782. Experiments exploring these questions would have been probably more impactful.

We have performed some preliminary Ca++ homeostasis experiments using HEK cells transfected with CALR^{del52} vs wildtype and analysed using the fura-2 technique (PMID 9278242). These did show some preliminary differences shown below, however we felt that these preliminary findings were beyond the scope of the present study.

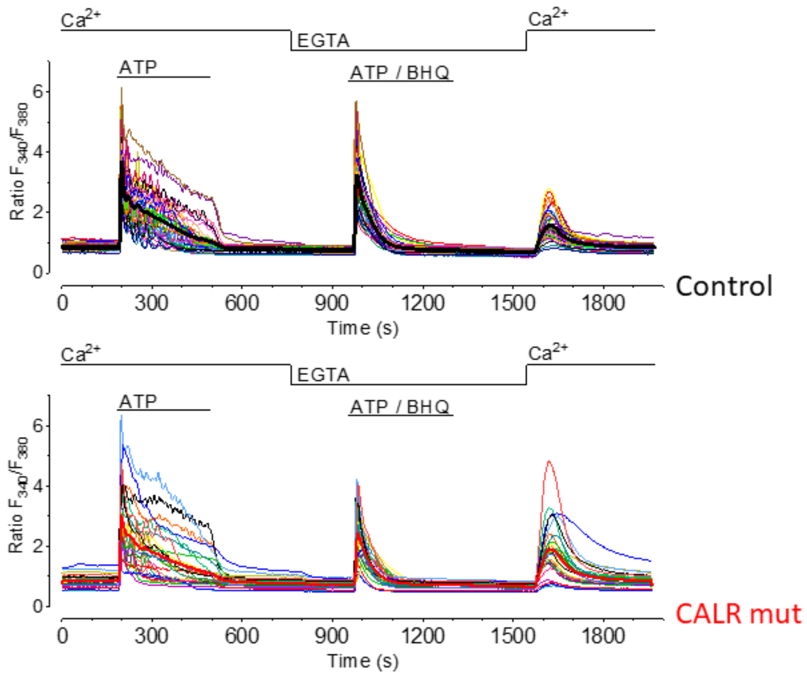

***Figure 1**: Investigation of the impact of CALR mutations in the cellular Ca2+ homeostasis of HEK293 cells using the fura-2 technique.*

9) In the last section, the author suggest that they are able to target the "fragile CALR-TpoR-glycosylation complex". This appears to be contradicting the fact that their antibody targets the mutant c-terminus. How do they explain that their antibody targets the tail and would still be able to disrupt the glycosylation binding?

We have corrected the wording in discussion and more clearly state our revised model based on new mechanistic data.

10) The last two sentences of the last paragraph of the Discussion are simply wrong: Wild-type CALR acts as a "eat me" signal at the surface of stressed cells, and is the ligand of LRP1R that is present on the surface of macrophages. CALR is not the ligand to CD47. CD47 is a ubiquitously expressed protein and acts as a constitutive "Don't eat me" signal at the cell surface. CD47 binds the SIRPα on the surface of macrophage to convey that signal.

We apologise and have corrected this sentence.

11) A number of key references are missing or not correctly cited. The data on requirement of the N-domain of CALR mutants and of the tail for TPOR activation are in Chachoua et al. Blood. 2016;127:1325-1335; interaction and traffic are in Masubuchi et al. Leukemia. 2020 Feb;34(2):499-509. also shown the requirement for oligomerization of mutant CALR is in Araki et al. Leukemia. 2019 Jan;33(1):122-131.

All of these references have been added.

12) The authors should discuss how the cleavage of the last portion of the C-tail (including the epitope) recently reported at ASH impacts the binding of the antibody.

On repeated experiments, we did not observe cleavage of CALR protein, in cell lysates or bound to TpoR (new **Fig 2F**). After 8 hours in conditioned media, eventual proteolytic cleavage of mutant CALR was observed which was partially inhibited by 4D7 (see figure above). However, as stated, we are not confident that this phenomenon is directly related to the TPO-independent proliferation induced by mutant CALR, or the biological inhibition of proliferation by 4D7.

In conclusion, the manuscript offers encouraging in vitro data of the capacity of the 4D7 monoclonal rat antibody to prevent the proliferation and differentiation of CALR mutated

cells. It stops short of offering a mechanistic explanation of how the antibody exerts this effect and the in vivo effects are not explored. Some claims of the manuscript are stretched and should be readjusted (see comments hereabove). There are some problems with the first panel of the first figure and two sentences at the end of the discussion that absolutely need to be corrected before considering further this manuscript. The major weakness beyond the lack of mechanism remains absence of any in vivo data, and this should be addressed as it would help also the establishment of mechanism.

We hope that we have sufficiently addressed the cell surface binding and mechanism with evidence for in vivo efficacy. All other comments have been addressed above in detail.

Overall comments from Referee #3: At a very minimum the evidence for binding of the antibody to cell surface CALR mutant in cells that have MPL and cells that do not should be provided, and for the former evidence that inhibition of MPL phosphorylation, signaling should be clear, as well as the issue with stabilization of plasma free mutant CALR versus shedding. Evidence of this on primary cells and/or some in vivo data in KI mice would be ideal.

We trust that we have provided sufficient new data to show signaling and mechanistic insights into the action of 4D7.

1st Revision - Editorial Decision 8th Dec 2021

Dear Dr. Thomas,

Thank you for the submission of your revised manuscript to our editorial offices. I have now received the reports from the three referees that were asked to re-evaluate your study, you will find below. As you will see, all three referees have remaining concerns and/or suggestion to improve the manuscript I ask you to address in a final revised version of the manuscript. Please also provide a point-by-point-response addressing these points.

During cross-commenting, referee #3 also stated:
'What is lacking is to show the effects of the antibody on PMF CD34+ cells in presence of TPO and whether it restores a response to TPO to TF1 after antibody treatment. It is important because if there is a normal response to TPO it means that the treatment will be efficient in selectively targeting the clone.

A minor point is that the results on immunodeficient mice are now presented like they have transplanted PMF CD34 cells. But in fact they are tracking TF1 cells.'

Please address also these points in the final revised manuscript.

Moreover, I have these editorial requests:

- Please provide an abstract written in present tense and remove the data from the abstract.

- Please make sure that the number "n" for how many independent experiments were performed, their nature (biological versus technical replicates), the bars and error bars (e.g. SEM, SD) and the test used to calculate p-values is indicated in the respective figure legends (main and EV figures), and that statistical testing has been done where applicable. Please avoid phrases like 'independent experiment', but clearly state if these were biological or technical replicates. Please add complete statistical testing to all diagrams. Please also indicate (e.g. with n.s.) if testing was performed, but the differences are not significant. Presently, many diagrams show no or only partial statistics.

- Please add scale bars of similar style and thickness to all the microscopic images, using clearly visible black or white bars (depending on the background). Please place these in the lower right corner of the images. Please do not write on or near the bars in the image but define the size in the respective figure legend. Please do not indicate the magnification in the images.

- As they are significantly cropped, please provide the source data for the Western blots shown in the manuscript (main and EV figures). The source data will be published in separate source data files online along with the accepted manuscript and will be linked to the relevant figures. Please submit scans of entire gels or blots together with the final revised manuscript. Please include size markers for scans of entire gels, label the scans with figure and panel number, and upload one PDF file per figure.

- There is a figure EV5 uploaded, but not called out in the text and no legend is provided. Moreover, this seems to be a table. Please upload this appropriately (as Table EV1?) and add callouts and a legend to the manuscript text (after the EV figure legends).

- Please add a formal 'Data Availability' section to the manuscript (after the methods), also if no large datasets have been submitted to a public database. Please state there 'No large primary datasets have been generated and deposited'. Further, please indicate this clearly in the author checklist.

- Please enter all the funding information also into our submission system and make sure this is complete and similar to the one mentioned in the acknowledgements of the manuscript text file.

- Finally, please find attached a word file of the manuscript text (provided by our publisher) with a few changes and queries we ask you to include in your final manuscript text. Please provide your final manuscript file with track changes, in order that we can see any modifications done.

In addition, I would need from you:
- a short, two-sentence summary of the manuscript (not more than 35 words).
- two to four short bullet points highlighting the key findings of your study.
- a schematic summary figure (in jpeg or tiff format with the exact width of 550 pixels and a height of not more than 400 pixels) that can be used as a visual synopsis on our website.

I look forward to seeing the final revised version of your manuscript when it is ready. Please let me know if you have questions regarding the revision.

Please use this link to submit your revision: https://embor.msubmit.net/cgi-bin/main.plex

Yours sincerely,

Achim Breiling
Editor
EMBO Reports

Referee #1:

In general, the manuscript is much improved and the authors have clearly benefited from the corrections and suggestions provided by the Reviewers.

However, some issues still remain:

Major Points

Point #1
Figure 1D, EV1E are both missing the CALR-WT control.
The authors are comparing ectopic over-expression of mutant CALR to endogenous expression of CALR in TF1 cells, which is not a fair comparison. Furthermore, data shown in subsequent figures (e.g. Figure EV3E) suggests parental TF1 cells do not express CALR. TF-1 cells are erythroid lineage and CALR is most highly expressed in megakaryocytic lineage cells, so this is not surprising. Figure EV2A shows TF1-CALRWT cells so these could be used as controls for surface mutant CALR expression.

Furthermore, the authors do not provide any data demonstrating that 4D7 binds to the cell surface of primary CALR-mutant MPN samples. This should be added.

Minor Points:

Point #1
In Figure 2F - the monomer detected using the wtCALR antibody is < 50kD - if the authors are detecting the same protein (namely mutant CALR) with both antibodies (i.e. wtCALR and mCALR) shouldn't the monomer size be the same in the wtCALR and mCALR panels?
In Figure EV4A the monomer sizes for wtCALR and mCALR seems to be the same - panels 2F and EV4A appear to show the same thing - why not just show one of these?

Point #2
In the reply to review the authors state: "Whilst a knock-in model would be beneficial, our data have shown that the human TPO receptor is necessary for the pathogenic effect of the CALR mutation. Therefore, it would be necessary to generate a mouse model that not only expresses mutant CALR, but also the human TPO receptor"

This statement is incorrect. A mutant CALR knockin mouse expressing endogenous mouse Mpl and the human mutant CALR C-terminus has been published and results in a disease phenotype identical to human CALR-mutant myeloproliferative neoplasm (MPN) - PMID: 29282219.

It is questionable how disease-relevant the models presented by the authors in the re-submission are. Injecting cell lines into immunocompromised mice is quite far from human MPN, particularly given the fact that multiple knockin and transgenic mutant CALR models have been published (including the one listed above). However, given the primary MPN sample ex vivo data, together with the improvements in the mechanistic data, I am willing to overlook the deficiencies of the in vivo models. However, the limitations of the in vivo models used in the manuscript should be acknowledged by the authors.

Point #3
It is striking how many factual inaccuracies the authors had in their original submission and again there are inaccuracies in this re-submission. This dampens enthusiasm for the manuscript. Another inaccuracy is contained in the discussion:

In the discussion the authors state that mutant CALR Is present on the cell surface and reference papers by Elf et al from 2016 and 2018. However, neither of these papers demonstrate that mutant CALR is present on the cell surface.

The correct references for this point are listed below and these should be corrected.

Pecquet C, Chachoua I, Roy A, et al. Calreticulin mutants as oncogenic rogue chaperones for TpoR and traffic-defective pathogenic TpoR mutants. Blood. 2019; 133(25):2669-2681.

Balligand T, Achouri Y, Pecquet C, et al. Knock-in of murine Calr del52 induces essential thrombocythemia with slow-rising

dominance in mice and reveals key role of Calr exon 9 in cardiac development. Leukemia 2020 Feb;34(2):510-521.

Finally, none of the changes in the manuscript are highlighted/tracked and the actual figures are not labelled making in unnecessarily laborious for the Reviewer to understand how the manuscript has been altered and where new data is located.

Referee #2:

Please, find attached my review of the revised manuscript entitled " Selective targeting of human CALR+ myelofibrosis with a neoepitope-directed monoclonal antibody" by Tvorogo et al., and numbered # EMBOR-2021-52904V2.

Myeloproliferative neoplasms are clonal hematological diseases that lead to overproduction of myeloid cells. They are due to mutations in molecules that activate the JAK2/STAT pathway. CALR mutations (CALRmut) are one of these mutations that lead to a protein with a new C-terminal tail and loss of endoplasmic reticulum retrieval motif. These mutants bind to the MPL receptor at the cell surface and activate it. However, their mechanism of action remains incompletely understood and targeted therapy does not exist either.

In this study, the authors have generated a monoclonal antibody against the mutated tail of CALRmut and have investigated its role in targeting CALRmut cell lines and primary cells from patients. They showed that this strategy efficiently and selectively targets the mutated cells in contrast to normal cells. In the revised manuscript, the authors have added important experiments to decipher the mechanism of action of this antibody. First, they show that this antibody can bind specifically to the CALRmut in cell lines by flow cytometry. Second, they provide evidence that the targeting by the antibody is mainly due to the disruption of the CALRmut/MPL interaction leading to the inhibition of cell signaling and apoptosis associated with an increased in CALRmut release in the medium. Third, they provide some in vivo studies showing the efficacy of the antibody after injection of CALRmut overexpressing cell lines. Even if these latter experiments are not really the gold standard to study the impact of the antibody on the disease development, it has the merit to provide some data on the antitumorigenic effect of the antibody on CALRmut cells.

Overall, this is a very original study and valuable work that is well conducted with state-of-the-art techniques. I think it would be important for MPN community.
I have still some minor comments to improve the text (introduction, title, abstract, results section). My concerns are detailed in the letter to the authors.

After these minor revisions, I think that this work can be of interest for the EMBO Reports audience.

Minor:
I think that the authors focus too much on myelofibrosis in the title, introduction and abstract. In fact, the CALR mutations are recurrently mutated in both essential thrombocythemia and myelofibrosis accounting for 30% of myeloproliferative neoplasms (MPN). Thus, the antibody against CALRmut could be an efficient targeted therapy in both diseases. The authors should rewrite a bit the abstract and the introduction (first paragraph) considering also the presence of the CALR mutation in essential thrombocythemia rather than only in myelofibrosis.
Moreover, the title is overstated since the authors did not show that the antibody targets the myelofibrosis, which is a disease characterized by specific hematological features and bone marrow fibrosis. Their work shows that the neoepitope-directed monoclonal antibody targets the CALRmut cells in the context of myeloproliferative neoplasms. I think that the authors should consider to change the title. I propose: "Selective targeting of human CALR mutated cells with a neoepitope-directed monoclonal antibody in myeloproliferative neoplasms".

In the introduction:
"whether TpoR activation occurs before or after cell surface exposure and whether or not it is accessible to an extracellularly-acting therapeutic is not clear (How et al, 2019)". This sentence is imprecise since the full activation of signaling by CALR mut requires cell surface localization of TpoR (Pecquet et al. blood, 2029).

The figure 1D is very interesting but I think that the conclusion should be downmodulated. Indeed, the antibody also binds to the wild type TF-1 TpoR even if it is at a lesser extent than in the mutant CALR-expressing cells.

Concerning the figure EV, the authors should verify their correct insertion in the text:
I think the EV2E is probably the EV3E page 6 second paragraph

----

Referee #3:

The revised version of the manuscript by Tvorogov et al. presents additional evidence and answers nicely to some of the points raised in the previous revision round.

Although the points are now stronger, there are still a number of issues that should be addressed. These are now apparent as the previous comments addressed major interpretation issues linked to stabilization versus shedding and other major experimental matters. The current issues are:

1. In the Introduction an emphasis is put on myelofibrosis, but the CALR mutations are also quite prevalent in essential thrombocythemia, a disease with a much better prognosis. The reader that is not specialist might understand that CALR mutants are specific to myelofibrosis, and this is not factually correct. In other words CALR mutants are targets of both ET and myelofibrosis and it may be that factors other than CALR mutants may explain the severe evolution of myelofibrosis.

2. At page 4, in discussion and in Figure 2G the authors make a number of statements that are factually incorrect. First, they state that the antibody is directed against an unstructured part of the C-terminus of the CALR mutants. In reality the authors do not explore the structural aspects of the mutant CALR and they cannot conclude it is unstructured based on their data or the fact that they obtained an antibody. In fact the region will be published soon by several groups to be structured. Second, they make the statement that the disulfide bonding explains activation of MPL TPOR, again they do not have these data and it is not clear how their extracellular antibody would prevent disulfide bond formation, which if real it occurs in the ER. Their statement regarding disulfide linked dimers of CALR relies on western blotting in denaturing conditions, thus not reproducing the actual structure of native CALR mutant. No conclusion can be drawn from such experiments and the cartoon in Figure 2G is misleading. In fact, it was shown that cysteines of the mutant tail were not required for activation of the receptor (Elf et al., 2016) and data that will be published soon will further demonstrate that C-terminal cysteines are neither involved in activation nor in oligomerization of CALR mutant. Authors also suggest that the 4D7 antibody prevents disulfide bond formation. Even if such species existed, this is wrong as their antibody simply reduces the co-immunoprecipitation of both monomeric and dimeric CALR mutants.

3. Caution should be exerted with respect to the interpretation of the shedding experiments as the levels of TPOR in cell lines that are transduced are excessive and endocytosis machinery mechanisms maybe saturated. In primary cells it is likely that with the lower levels of TPOR at the surface, almost all complexes are rapidly endocytosed after exposure at the surface and activation. Experiments of shedding on primary cells would be required to make this point.

4. Page 6 and Fig. 2F, how can the authors explain that addition of 4D7 reduces the disulfide linked dimer form of mutant CALR? If the antibody prevents disulfide bonding at the surface, this maybe a postlysis event, therefore artifactual. If the disulfide bond is prevented for intracellular forms, then how does 4D7 arrive in the ER? Did authors test a mutant lacking the very last residues that exhibit no Cys residues?

5. Overall, the mechanism of action of the 4D7 antibody should be validated in primary cells or, best, in vivo. Can the authors prove that in vivo, or in primary cells, the antibody is able to shed CALR mutants?

What is lacking is to show the effects of the antibody on PMF CD34+ cells in presence of TPO and whether it restores a response to TPO to TF1 after antibody treatment. It is important because if there is a normal response to TPO it means that the treatment will be efficient in selectively targeting the clone.

A minor point is that the results on immunodeficient mice are now presented like they have transplanted PMF CD34 cells. But in fact they are tracking TF1 cells.

We thank the reviewers for their helpful comments and are pleased to present a final manuscript for consideration of publication in *EMBO Reports* with further revisions. We have addressed all noted editorial requests. We sincerely apologise for not highlighting our changes in the previous revision. This was not included as we believed the changes and improvements to our submission were so substantial, it would make it difficult for the reviewers to compare. We are pleased with the reviewers' comments that "In general, the manuscript is much improved" and "additional evidence and answers nicely to some of the points raised in the previous revision round". We are pleased with comments from Reviewer #2, "Overall, this is a very original study, well conducted, with state-of-the-art techniques. I think it would be important for MPN community."
We submit here a point-by-point response to the comments.

Overall comments:

'What is lacking is to show the effects of the antibody on PMF CD34+ cells in presence of TPO and whether it restores a response to TPO. It is important because if there is a normal response to TPO it means that the treatment will be efficient in selectively targeting the clone.

We agree this is a critical point with therapeutic implications. We have performed rescue experiments using TF-1 TpoR $CALR^{del61}$ cells. We see partial rescue by TPO in the presence of 4D7 antibody (new **Fig EV4B**). This suggests that TpoR is in a different conformation when mutant CALR was previously bound and is inefficient at responding to TPO, consistent with a recent report in the setting of amegakaryocytic thrombocytopenia (PMID 34010413). We also performed rescue experiments in our megakaryocyte colony assays with primary CD34+ $CALR^{del52}$ samples. Consistently, we see partial but not full rescue in megakaryocyte colonies in the presence of 4D7 plus TPO, consistent with our cell line data (new **Fig EV4C**).

A minor point is that the results on immunodeficient mice are now presented like they have transplanted PMF CD34 cells. But in fact they are tracking TF1 cells.

Correct, we only used TF-1 cells. This has been emphasized in both the results and legend.

Reviewer #1

Figure 1D, EV1E are both missing the CALR-WT control. The authors are comparing ectopic over-expression of mutant CALR to endogenous expression of CALR in TF1 cells, which is not a fair comparison.

Agree and have now performed additional experiments with $CALR^{WT}$ (**Fig 1D and EV1E**).

Furthermore, data shown in subsequent figures (e.g. Figure EV3E) suggests parental TF-1 cells do not express CALR. TF-1 cells are erythroid lineage and CALR is most highly expressed in megakaryocytic lineage cells, so this is not surprising. Figure EV2A shows TF1-CALRWT cells so these could be used as controls for surface mutant CALR expression.
We apologise for the confusion. We clearly see endogenous wildtype CALR protein (a ubiquitous scaffold protein) expressed in TF-1 cell lysate (**Fig 2G**, right panels and **Fig EV4A** right panels) when we use anti-wtCALR Cell Signalling antibody as a band just above 50 kDa. The panels on the left of **Fig 2G** and **Fig EV4A** are TpoR immunoprecipitates, which do not have detectable wild type CALR as expected. The panels on the right are whole cell lysates with an arrow now clearly labelled “wtCALR” and “mCALR” for clarification.

Furthermore, the authors do not provide any data demonstrating that 4D7 binds to the cell surface of primary CALR-mutant MPN samples.
As the reviewers clearly state, multiple publications have shown cell surface expression of mutant CALR. We can clearly show binding of 4D7 to TF-1 cells over-expressing mutant CALR and TpoR (modified **Fig. 1D**). TpoR is not abundantly expressed on primary haematopoietic cells and its cell surface expression appears to be tightly regulated (Wilms et al, Science, 2020, PMID 32029621), as correctly pointed out by reviewer #3 (comment #4). Nevertheless, in primary myelofibrosis CALR+ cells, we clearly show inhibition of downstream signalling in $CALR^{del52}$ samples but not $JAK2^{V617F}$ samples (new **Fig 2D** and new **Fig EV3D**), indicating that 4D7 must interact with cell surface TpoR and clearly disrupts TPO-independent megakaryocyte formation from primary progenitors (Fig 3 A-H).

In Figure 2F - the monomer detected using the wtCALR antibody is < 50kD - if the authors are detecting the same protein (namely mutant CALR) with both antibodies (i.e. wtCALR and mCALR) shouldn't the monomer size be the same in the wtCALR and mCALR panels?
We apologise for not labelling our non-reducing Western blots with more detail. As demonstrated in previous publications (PMID 31462733), $CALR^{del52}$ is actually smaller in size (< 50 kDa) than wildtype CALR (above 50 kDa) due to the size of the deletion. wtCALR and mutCALR can be clearly distinguished using anti-wildtype CALR antibody (Cell Signalling # (#12238)) or mutant specific antibodies (CAL2, Dianova).

In Figure EV4A the monomer sizes for wtCALR and mCALR seems to be the same - panels 2F and EV4A appear to show the same thing - why not just show one of these?
As stated above and from our improved figures (modified **Fig 2G** and **EV4A**) complete with labelled arrows, $CALR^{WT}$ and $CALR^{del52}$ do not run at the same size on non-reducing or reducing gels. This is consistent with a number of publications (PMID 26608331, PMID

33821991, PMID 24325359, etc). Both **Fig 2G** and **Fig EV4A** are of value as they both demonstrate disruption of CALR dimers and CALR monomers bound to TpoR after 4D7 treatment. Also, additional mutant forms of CALR in experiments was requested by one of the reviewers in previous round of revisions. Consistent data showing the same mechanism of action across different CALR mutations in patients with MPN is important for demonstrating generality and underscores the utility of an immunotherapeutic approach.

In the reply to review the authors state: "Whilst a knock-in model would be beneficial, our data have shown that the human TPO receptor is necessary for the pathogenic effect of the CALR mutation. Therefore, it would be necessary to generate a mouse model that not only expresses mutant CALR, but also the human TPO receptor". This statement is incorrect. A mutant CALR knockin mouse expressing endogenous mouse Mpl and the human mutant CALR C-terminus has been published and results in a disease phenotype identical to human CALR-mutant myeloproliferative neoplasm (MPN) - PMID: 29282219. It is questionable how disease-relevant the models presented by the authors in the re-submission are. Injecting cell lines into immunocompromised mice is quite far from human MPN, particularly given the fact that multiple knockin and transgenic mutant CALR models have been published (including the one listed above). However, given the primary MPN sample ex vivo data, together with the improvements in the mechanistic data, I am willing to overlook the deficiencies of the in vivo models. However, the limitations of the in vivo models used in the manuscript should be acknowledged by the authors.
We appreciate these comments and the useful reference. We have included a sentence in the discussion that further experiments in relevant mouse models, especially heterozygous models, should be performed. It is conceivable that our antibody indirectly interferes with non-homologous residues in the human vs mouse TpoR that interact with mutant CALR. We agree our model is not entirely disease relevant. Nevertheless, we believe it is very informative for clinical development. It tells us that the complementarity-determining region of the antibody 4D7 does not interact negatively with healthy mammalian tissue and does not cause toxicity at concentrations > 5 x in vitro effective concentrations. Secondly, it tells us that the pharmacokinetic profile observed for 4D7 is similar to expected IgG2a antibodies and as such, is sufficient to result in decreased tumour burden, prolongation of survival or decreased bone marrow engraftment of tumours that are aggressively driven by mutant CALR.

It is striking how many factual inaccuracies the authors had in their original submission and again there are inaccuracies in this re-submission. This dampens enthusiasm for the manuscript. Another inaccuracy is contained in the discussion: In the discussion the authors

state that mutant CALR Is present on the cell surface and reference papers by Elf et al from 2016 and 2018. However, neither of these papers demonstrate that mutant CALR is present on the cell surface. The correct references for this point are listed.
We apologise for these inaccuracies. This is a rapidly expanding field, and we are endeavouring to keep up with the literature. We thank you for these references and have made corrections.

Finally, none of the changes in the manuscript are highlighted/tracked and the actual figures are not labelled making in unnecessarily laborious for the Reviewer to understand how the manuscript has been altered and where new data is located.
We sincerely apologise for not marking changes and for the confusion this caused. We added a lot of new data in our first revision as well as substantial textual changes. In our 2nd revision we have now highlighted/tracked all new figures and text and labelled the actual figures. We note that guidelines for authors specifically states “do not label figures” in the *EMBO Reports* website.

Referee #2:
Authors focus too much on myelofibrosis in the title, introduction and abstract. …I propose: "Selective targeting of human CALR mutated cells with a neoepitope-directed monoclonal antibody in myeloproliferative neoplasms".
We have changed the title in accordance with this recommendation, remaining within character limitations.

Introduction: "whether TpoR activation occurs before or after cell surface exposure and whether or not it is accessible to an extracellularly-acting therapeutic is not clear (How et al, 2019)". This sentence is imprecise since the full activation of signaling by CALR mut requires cell surface localization of TpoR (Pecquet et al. blood, 2029).
We have added this reference and reworded this statement to emphasise the importance of cell surface localization.

The figure 1D is very interesting but I think that the conclusion should be downmodulated. Indeed, the antibody also binds to the wild type TF-1 TpoR even if it is at a lesser extent than in the mutant CALR-expressing cells.
We have revised our comments in the results section and added extra controls. We see more than a log MFI difference between antibody binding TF-1 CALR^{del61} vs CALR^{WT}.

Concerning the figure EV, the authors should verify their correct insertion in the text: I think the EV2E is probably the EV3E page 6 second paragraph

Correct. This has now been labelled clearly.

Referee #3

1. In the Introduction an emphasis is put on myelofibrosis, but the CALR mutations are also quite prevalent in essential thrombocythemia, a disease with a much better prognosis.

The title and introduction now also reflect the importance of CALR as a mutation in both essential thrombocythemia and myelofibrosis.

2. At page 4, in discussion and in Figure 2G the authors make a number of statements that are factually incorrect. First, they state that the antibody is directed against an unstructured part of the C-terminus of the CALR mutants. In reality the authors do not explore the structural aspects of the mutant CALR and they cannot conclude it is unstructured based on their data or the fact that they obtained an antibody. In fact the region will be published soon by several groups to be structured.

Thank you for this explanatory piece of information. We agree we do not know the structure of the neoepitope based on available data and we have removed this wording.

3. Second, they make the statement that the disulfide bonding explains activation of MPL TPOR, again they do not have these data and it is not clear how their extracellular antibody would prevent disulfide bond formation, which if real it occurs in the ER. Their statement regarding disulfide linked dimers of CALR relies on western blotting in denaturing conditions, thus not reproducing the actual structure of native CALR mutant. No conclusion can be drawn from such experiments and the cartoon in Figure 2G is misleading. In fact, it was shown that cysteines of the mutant tail were not required for activation of the receptor (Elf et al., 2016) and data that will be published soon will further demonstrate that C-terminal cysteines are neither involved in activation nor in oligomerization of CALR mutant. Authors also suggest that the 4D7 antibody prevents disulfide bond formation. Even if such species existed, this is wrong as their antibody simply reduces the co-immunoprecipitation of both monomeric and dimeric CALR mutants.

Thank you for critical data. Agreed, with do not have definitive proof that the cysteines are required for dimerization. These experiments would require further constructs to be engineered and then tested. We used non-reducing conditions to clearly distinguish CALR from immunoglobulin in an IP, as both run at a similar size. To further avoid IgG artefacts, we used covalently bound anti-FLAG IgG from Sigma as described in the Materials and Methods. We have removed reference to disulfide-mediated dimerization in text and model.

4. Caution should be exerted with respect to the interpretation of the shedding experiments as the levels of TPOR in cell lines that are transduced are excessive and endocytosis machinery mechanisms maybe saturated. In primary cells it is likely that with the lower levels of TPOR at the surface, almost all complexes are rapidly endocytosed after exposure at the surface and activation. Experiments of shedding on primary cells would be required to make this point.

As described in previous rebuttal we have already removed all mention of shedding in the manuscript and focus on TpoR/mutCALR complex.

5. Page 6 and Fig. 2F, how can the authors explain that addition of 4D7 reduces the disulfide linked dimer form of mutant CALR? If the antibody prevents disulfide bonding at the surface, this maybe a postlysis event, therefore artifactual. If the disulfide bond is prevented for intracellular forms, then how does 4D7 arrive in the ER? Did authors test a mutant lacking the very last residues that exhibit no Cys residues?

See comments above. We do not have definitive proof that CALR consists of disulfide linked dimers. We used 50 mM iodoacetamide to block artefactual disulfide linkage post-lysis (as described in the Materials and Methods). We are convinced CALR consists of at least a dimeric form as others have published (PMID 33821991).

6. Overall, the mechanism of action of the 4D7 antibody should be validated in primary cells or, best, in vivo. Can the authors prove that in vivo, or in primary cells, the antibody is able to shed CALR mutants?

We have performed signalling experiments in primary cells which convincingly show 4D7 can downregulate STAT and ERK phosphorylation (new **Fig 2D** and new **Fig EV3D**). We have abandoned shedding as a mechanism of action from the previous round of review. The amount of sample required for immunoprecipitation experiments with primary cells is not feasible (> 1L blood).

7. What is lacking is to show the effects of the antibody on PMF CD34+ cells in presence of TPO and whether it restores a response to TPO to TF1 after antibody treatment. It is important because if there is a normal response to TPO it means that the treatment will be efficient in selectively targeting the clone.

This is an excellent suggestion. See response to overall comments at the beginning.

A minor point is that the results on immunodeficient mice are now presented like they have transplanted PMF CD34 cells. But in fact they are tracking TF1 cells.

Agreed and this has been emphasized in the text.

2nd Revision - Editorial Decision 20th Jan 2022

Dear Dr. Thomas,

Thank you for the submission of your further revised manuscript to our editorial offices. We are nearly there. I have now received the reports from the three referees that were asked to re-evaluate the manuscript, which I include below for your information. As you can see, the referees now support publication of your study in EMBO reports. Referee #1 has some final comment, I ask you to address in a really final version of the manuscript

Moreover, I have these editorial requests:

- Please provide the source data with more information. For each file, please make sure that all the blots are labelled (indicating what protein was detected), that they are grouped together panel-wise and that it is indicated to which panel they belong, that the region shown in the final figure is marked by a box, and that the sizes of the marker bands are indicated throughout.

- The synopsis image provided has not the right format. This should be in jpeg or tiff format with the exact width of 550 pixels and a height of not more than 400 pixels. Please provide such an image.

Finally, we updated our journal's competing interests policy in January 2022 and request authors to consider both actual and perceived competing interests. Please review the policy https://www.embopress.org/competing-interests and update your competing interests if necessary.

I look forward to seeing the final revised version of your manuscript when it is ready. Please let me know if you have questions regarding the revision.

Yours sincerely,

Achim Breiling
Editor
EMBO Reports

Referee #1:

The manuscript is improved with the revisions.

The authors are unable to show that 4D7 binds to the cell surface of CALR-mutant primary MPN cells. They show indirect evidence that suggests it might e.g. inhibition of JAK-STAT signaling in CALR-mutant primary MF cells treated with 4D7 (figure 2D) (not seen in JAK2-mutant primary MF cells treated with 4D7, Fig EV3D) and partial rescue of growth of CALR-mutant primary MPN cells treated with 4D7, with the addition of TPO.

Minor comments:

1. Please clarify what type of primary MF cells are shown in the data in Figure 2D and Fig EV3D (e.g. peripheral blood, unfractionated bone marrow, CD34+-enriched bone marrow, megakaryocyte-enriched bone marrow?)

2. The reference by Kepp et al. should be removed - this paper does not show any data indicating mutant CALR is expressed on the cell surface in primary CALR-mutant MPN cells - the manuscript is about secreted mutant CALR and contains no primary data.

3. Figure EV1E does not show CALR-WT cells stained with 4D7 antibody; Figure 1D does however show CALR-WT cells stained with 4D7 antibody (this is adequate).

Referee #2:

Please, find attached my review of the revised manuscript entitled " Selective targeting of human CALR+ myelofibrosis with a neoepitope-directed monoclonal antibody" by Tvorogov et al., and numbered # EMBOR-2021-52904V3.

Myeloproliferative neoplasms are clonal hematological diseases that lead to overproduction of myeloid cells. They are due to mutations in molecules that activate the JAK2/STAT pathway. CALR mutations (CALRmut) are one of these mutations that lead to a protein with a new C-terminal tail and loss of endoplasmic reticulum retrieval motif. These mutants bind to the MPL receptor at the cell surface and activate it. However, their mechanism of action remains incompletely understood and targeted therapy

does not exist either.

In this study, the authors have generated a monoclonal antibody against the mutated tail of CALRmut and have investigated its role in targeting CALRmut cell lines and primary cells from patients. They showed that this strategy efficiently and selectively targets the mutated cells in contrast to normal cells.
In the successive revised manuscript, the authors have addressed important points to decipher the mechanism of action of this antibody. They have considered the text changes suggested by the reviewers, which makes the manuscript more accurate, particularly concerning the previous works in the domain. I still suggest to change "stem cells" into "progenitors" when the authors mention CD34+ progenitors.
In my opinion, this is a study of great importance for MPN community and patients care. Moreover, I think that this work deserves to be published in the EMBO Reports.

Referee #3:

The authors answered the concerns and did the best to clarify all issues raised during the review.

The authors have addressed all minor editorial requests.

Dr. Daniel Thomas
The University of Adelaide
Adelaide Medical School
North Terrace
Adelaide, South Australia 5001
Australia

Dear Dr. Thomas,

I am very pleased to accept your manuscript for publication in the next available issue of EMBO reports. Thank you for your contribution to our journal.

At the end of this email I include important information about how to proceed. Please ensure that you take the time to read the information and complete and return the necessary forms to allow us to publish your manuscript as quickly as possible.

As part of the EMBO publication's Transparent Editorial Process, EMBO reports publishes online a Review Process File to accompany accepted manuscripts. As you are aware, this File will be published in conjunction with your paper and will include the referee reports, your point-by-point response and all pertinent correspondence relating to the manuscript.

If you do NOT want this File to be published, please inform the editorial office within 2 days, if you have not done so already, otherwise the File will be published by default [contact: emboreports@embo.org]. If you do opt out, the Review Process File link will point to the following statement: "No Review Process File is available with this article, as the authors have chosen not to make the review process public in this case."

Should you be planning a Press Release on your article, please get in contact with emboreports@wiley.com as early as possible, in order to coordinate publication and release dates.

Thank you again for your contribution to EMBO reports and congratulations on a successful publication. Please consider us again in the future for your most exciting work.

Yours sincerely,

Achim Breiling
Editor
EMBO Reports

****

THINGS TO DO NOW:

You will receive proofs by e-mail approximately 2-3 weeks after all relevant files have been sent to our Production Office; you should return your corrections within 2 days of receiving the proofs.

Please inform us if there is likely to be any difficulty in reaching you at the above address at that time. Failure to meet our deadlines may result in a delay of publication, or publication without your corrections.

All further communications concerning your paper should quote reference number EMBOR-2021-52904V4 and be addressed to emboreports@wiley.com.

Should you be planning a Press Release on your article, please get in contact with emboreports@wiley.com as early as possible, in order to coordinate publication and release dates.

EMBO PRESS

YOU MUST COMPLETE ALL CELLS WITH A PINK BACKGROUND ↓

PLEASE NOTE THAT THIS CHECKLIST WILL BE PUBLISHED ALONGSIDE YOUR PAPER

Corresponding Author Name: Daniel Thomas
Journal Submitted to: Embo Reports
Manuscript Number: EMBOR-2021-52904

USEFUL LINKS FOR COMPLETING THIS FORM

http://www.antibodypedia.com
http://1degreebio.org
http://www.equator-network.org/reporting-guidelines/improving-bioscience-research-repor
http://grants.nih.gov/grants/olaw/olaw.htm
http://www.mrc.ac.uk/Ourresearch/Ethicsresearchguidance/Useofanimals/index.htm
http://ClinicalTrials.gov
http://www.consort-statement.org
http://www.consort-statement.org/checklists/view/32-consort/66-title
http://www.equator-network.org/reporting-guidelines/reporting-recommendations-for-tum
http://datadryad.org
http://figshare.com
http://www.ncbi.nlm.nih.gov/gap
http://www.ebi.ac.uk/ega
http://biomodels.net/
http://biomodels.net/miriam/
http://jjj.biochem.sun.ac.za
https://osp.od.nih.gov/biosafety-biosecurity-and-emerging-biotechnology/
http://www.selectagents.gov/

Reporting Checklist For Life Sciences Articles (Rev. June 2017)

This checklist is used to ensure good reporting standards and to improve the reproducibility of published results. These guidelines are consistent with the Principles and Guidelines for Reporting Preclinical Research issued by the NIH in 2014. Please follow the journal's authorship guidelines in preparing your manuscript.

A- Figures
1. Data
The data shown in figures should satisfy the following conditions:

- the data were obtained and processed according to the field's best practice and are presented to reflect the results of the experiments in an accurate and unbiased manner.
- figure panels include only data points, measurements or observations that can be compared to each other in a scientifically meaningful way.
- graphs include clearly labeled error bars for independent experiments and sample sizes. Unless justified, error bars should not be shown for technical replicates.
- if n< 5, the individual data points from each experiment should be plotted and any statistical test employed should be justified
- Source Data should be included to report the data underlying graphs. Please follow the guidelines set out in the author ship guidelines on Data Presentation.

2. Captions
Each figure caption should contain the following information, for each panel where they are relevant:

- a specification of the experimental system investigated (eg cell line, species name).
- the assay(s) and method(s) used to carry out the reported observations and measurements
- an explicit mention of the biological and chemical entity(ies) that are being measured.
- an explicit mention of the biological and chemical entity(ies) that are altered/varied/perturbed in a controlled manner.
- the exact sample size (n) for each experimental group/condition, given as a number, not a range;
- a description of the sample collection allowing the reader to understand whether the samples represent technical or biological replicates (including how many animals, litters, cultures, etc.).
- a statement of how many times the experiment shown was independently replicated in the laboratory.
- definitions of statistical methods and measures:
  - common tests, such as t-test (please specify whether paired vs. unpaired), simple χ2 tests, Wilcoxon and Mann-Whitney tests, can be unambiguously identified by name only, but more complex techniques should be described in the methods section;
  - are tests one-sided or two-sided?
  - are there adjustments for multiple comparisons?
  - exact statistical test results, e.g., P values = x but not P values < x;
  - definition of 'center values' as median or average;
  - definition of error bars as s.d. or s.e.m.

Any descriptions too long for the figure legend should be included in the methods section and/or with the source data.

In the pink boxes below, please ensure that the answers to the following questions are reported in the manuscript itself. Every question should be answered. If the question is not relevant to your research, please write NA (non applicable). We encourage you to include a specific subsection in the methods section for statistics, reagents, animal models and human subjects.

B- Statistics and general methods

Please fill out these boxes ↓ (Do not worry if you cannot see all your text once you press return)

1.a. How was the sample size chosen to ensure adequate power to detect a pre-specified effect size?	Patient samples were limited by our tissue bank and a relatively rare disease. Nonetheless, we were able to obtain statistically significant differences between CALR mutant and CALR wild type patient samples with sufficient numbers (n = 7) to achieve a power of 0.8 with an alpha value of 0.05. Mouse numbers for in vivo experiments were based on a power of 0.8 to detect a clinically meaningful decrease in tumour burden and mouse survival > 30%.
1.b. For animal studies, include a statement about sample size estimate even if no statistical methods were used.	Using the principle of reduction, we chose to use the least number of mice to achieve an alpha value of 0.05 and power of 0.8 to detect a clinically meaningful difference in survival > 40%, from 35 days to 60 days (an increase of 40%). According to our statistical analysis the approx. lower critical value for median survival is 24 days with an upper critical value of 52 days. An increased in median survival by a minimum of 40% will be of considerable clinical significance and will provide strong justification for a phase 1 clinical trial with a humanised version of 4D7 anti-mutant CALR antibody in patients with end stage CALR mutation positive myelofibrosis. Using the same rationale, a sample size wqas chosen to be sufficient to detect a decrease in tumour size in the chloroma mode. The mean tumour size in the IgG control at day 28 is anticipated to be 1125 mm3, based on past experience. We have a high probability of observing efficacy of our monoclonal antibody with a relatively few number of cages if a genuine biological effect was indeed present. A decrease in tumour size of 30% at 28 days would be clinically significant observation that would also facilitate consideration of a phase I study in humans. Our consistent criteria for is at least one dimension of tumour size (length, breadth, or depth) greater than or equal to 30 mm, or tumour
2. Describe inclusion/exclusion criteria if samples or animals were excluded from the analysis. Were the criteria pre-established?	Only 4 - 6week old healthy NSG male or female mice were used in our study.
3. Were any steps taken to minimize the effects of subjective bias when allocating animals/samples to treatment (e.g. randomization procedure)? If yes, please describe.	Treatments were administered by independent operators. No differences in age or mouse size were used to allocate treatment groups.
For animal studies, include a statement about randomization even if no randomization was used.	There was no randomization in our experiments given overall small numbers.
4.a. Were any steps taken to minimize the effects of subjective bias during group allocation or/and when assessing results (e.g. blinding of the investigator)? If yes please describe.	Yes, mice were allocated by a team of investigators to avoid subjective bias from one operator. There was no blinding in the first two in vivo experiments.
4.b. For animal studies, include a statement about blinding even if no blinding was done	No blinding was done, partially as we were using an in-house reagent.
5. For every figure, are statistical tests justified as appropriate?	Yes, each figure legend has sufficient information under the label data "data information"
Do the data meet the assumptions of the tests (e.g., normal distribution)? Describe any methods used to assess it.	Yes, all experimental data was assumed to have a normal distribution except as described for non-parametric populations.

Is there an estimate of variation within each group of data?	Yes. Error bars, SEMs and standard deviation shown as appropriate.
Is the variance similar between the groups that are being statistically compared?	Yes

C- Reagents

6. To show that antibodies were profiled for use in the system under study (assay and species), provide a citation, catalog number and/or clone number, supplementary information or reference to an antibody validation profile. e.g., Antibodypedia (see link list at top right), 1DegreeBio (see link list at top right).	Primary antibodies against pERK (#9101), pSTAT5 (#9359), ERK (#4695), Actin (#4970), Caspase 3 (#14220), wildtype CALR (#12238) were purchased from Cell Signalling. Primary antibodies against pSTAT1 (#612233), pSTAT3 (#612357), STAT1 (#610186), STAT3 (#610190), STAT5 (#610192) and Anti-phosphotyrosine 4G10 (#610012) were purchased from BD Biosciences. Mutant CALR monoclonal antibody CAL2 was purchased from Dianova (Hamburg, Germany). Immunoprecipitation of TpoR was performed in standard NP40 lysis buffer with additional 50 mM iodoacetamide to avoid any de novo disulfide bond formation post lysis. To reduce IgG background during immunoprecipitation anti-FLAG conjugated to magnetic beads (Sigma, #M8823) were used. For this same reason, anti-FLAG-HRP conjugated antibodies (Sigma, #A8592) were used in western blotting. IgG2a PE isotype control (ThermoFisher #12-4321-42)
7. Identify the source of cell lines and report if they were recently authenticated (e.g., by STR profiling) and tested for mycoplasma contamination.	All cell lines were authenticated by STR (Lopez lab, Thomas Lab, Leukemia Research Group) and tested for mycoplasma

* for all hyperlinks, please see the table at the top right of the document

D- Animal Models

8. Report species, strain, gender, age of animals and genetic modification status where applicable. Please detail housing and husbandry conditions and the source of animals.	4-6 week old NSG mice were used. Both males and females were utilised for this study. Mice were housed in Bioresources Facility at the South Australian Health and Medical Research Institutes BioResources Facility and were sourced from JAX labs.
9. For experiments involving live vertebrates, include a statement of compliance with ethical regulations and identify the committee(s) approving the experiments.	All animal experiments were approved by the South Australian Health and Medical Research Institutes ethics committee, ethics number SAM-21-018. All experiments were conducted in compliance with the ethical regulations and protocols approved as part of this application.
10. We recommend consulting the ARRIVE guidelines (see link list at top right) (PLoS Biol. 8(6), e1000412, 2010) to ensure that other relevant aspects of animal studies are adequately reported. See author guidelines, under 'Reporting Guidelines'. See also: NIH (see link list at top right) and MRC (see link list at top right) recommendations. Please confirm compliance.	We are compliant with ARRIVE guidelines.

E- Human Subjects

11. Identify the committee(s) approving the study protocol.	Umbilical cord blood (UCB) was collected with written consent from full-term deliveries at the Lyell McEwin Hospital (Adelaide, South Australia) or the Department of Obstetrics and Gynecology, Medical University of Graz (Austria) with institutional review board approval (IRB approval: 31-322 ex 18/19; HREC/20/WCHN/65).
12. Include a statement confirming that informed consent was obtained from all subjects and that the experiments conformed to the principles set out in the WMA Declaration of Helsinki and the Department of Health and Human Services Belmont Report.	Informed consent is obtained from all patients giving samples to the South Australian Cancer Research Biobank.
13. For publication of patient photos, include a statement confirming that consent to publish was obtained.	N/A
14. Report any restrictions on the availability (and/or on the use) of human data or samples.	All samples are de-identified in our paper and to our investigators.
15. Report the clinical trial registration number (at ClinicalTrials.gov or equivalent), where applicable.	N/A
16. For phase II and III randomized controlled trials, please refer to the CONSORT flow diagram (see link list at top right) and submit the CONSORT checklist (see link list at top right) with your submission. See author guidelines, under 'Reporting Guidelines'. Please confirm you have submitted this list.	N/A
17. For tumor marker prognostic studies, we recommend that you follow the REMARK reporting guidelines (see link list at top right). See author guidelines, under 'Reporting Guidelines'. Please confirm you have followed these guidelines.	N/A

F- Data Accessibility

18: Provide a "Data Availability" section at the end of the Materials & Methods, listing the accession codes for data generated in this study and deposited in a public database (e.g. RNA-Seq data: Gene Expression Omnibus GSE39462, Proteomics data: PRIDE PXD000208 etc.) Please refer to our author guidelines for 'Data Deposition'.<br><br>Data deposition in a public repository is mandatory for:<br>a. Protein, DNA and RNA sequences<br>b. Macromolecular structures<br>c. Crystallographic data for small molecules<br>d. Functional genomics data<br>e. Proteomics and molecular interactions	No large primary datasets have been generated and deposited
19. Deposition is strongly recommended for any datasets that are central and integral to the study; please consider the journal's data policy. If no structured public repository exists for a given data type, we encourage the provision of datasets in the manuscript as a Supplementary Document (see author guidelines under 'Expanded View' or in unstructured repositories such as Dryad (see link list at top right) or Figshare (see link list at top right).	No large primary datasets have been generated and deposited
20. Access to human clinical and genomic datasets should be provided with as few restrictions as possible while respecting ethical obligations to the patients and relevant medical and legal issues. If practically possible and compatible with the individual consent agreement used in the study, such data should be deposited in one of the major public access-controlled repositories such as dbGAP (see link list at top right) or EGA (see link list at top right).	No large primary datasets have been generated and deposited
21. Computational models that are central and integral to a study should be shared without restrictions and provided in a machine-readable form. The relevant accession numbers or links should be provided. When possible, standardized format (SBML, CellML) should be used instead of scripts (e.g. MATLAB). Authors are strongly encouraged to follow the MIRIAM guidelines (see link list at top right) and deposit their model in a public database such as Biomodels (see link list at top right) or JWS Online (see link list at top right). If computer source code is provided with the paper, it should be deposited in a public repository or included in supplementary information.	No computational models have been generated and deposited

G- Dual use research of concern

22. Could your study fall under dual use research restrictions? Please check biosecurity documents (see link list at top right) and list of select agents and toxins (APHIS/CDC) (see link list at top right). According to our biosecurity guidelines, provide a statement only if it could.	Not applicable. No biosecurity concerns.
